# Whole patient knowledge modeling of COVID-19 symptomatology reveals common molecular mechanisms

**DOI:** 10.3389/fmmed.2022.1035290

**Published:** 2023-01-04

**Authors:** Stephan Brock, David B. Jackson, Theodoros G. Soldatos, Klaus Hornischer, Anne Schäfer, Francesca Diella, Maximilian Y. Emmert, Simon P. Hoerstrup

**Affiliations:** ^1^ Molecular Health GmbH, Heidelberg, Germany; ^2^ SRH Hochschule, University of Applied Science, Heidelberg, Germany; ^3^ Institute for Regenerative Medicine, University of Zurich, Zurich, Switzerland; ^4^ Wyss Zurich, University of Zurich and ETH Zurich, Zurich, Switzerland; ^5^ Department of Cardiothoracic and Vascular Surgery, German Heart Institute Berlin, Berlin, Germany; ^6^ Department of Cardiovascular Surgery, Charité Universitätsmedizin Berlin, Berlin, Germany

**Keywords:** COVID-19, SARS-CoV-2, molecular mechanisms, evidence-based medicine, hypothesis generation, disease modelling

## Abstract

Infection with SARS-CoV-2 coronavirus causes systemic, multi-faceted COVID-19 disease. However, knowledge connecting its intricate clinical manifestations with molecular mechanisms remains fragmented. Deciphering the molecular basis of COVID-19 at the whole-patient level is paramount to the development of effective therapeutic approaches. With this goal in mind, we followed an iterative, expert-driven process to compile data published prior to and during the early stages of the pandemic into a comprehensive COVID-19 knowledge model. Recent updates to this model have also validated multiple earlier predictions, suggesting the importance of such knowledge frameworks in hypothesis generation and testing. Overall, our findings suggest that SARS-CoV-2 perturbs several specific mechanisms, unleashing a pathogenesis spectrum, ranging from “a perfect storm” triggered by acute hyper-inflammation, to accelerated aging in protracted “long COVID-19” syndromes. In this work, we shortly report on these findings that we share with the community *via* 1) a synopsis of key evidence associating COVID-19 symptoms and plausible mechanisms, with details presented within 2) the accompanying “COVID-19 Explorer” webserver, developed specifically for this purpose (found at https://covid19.molecularhealth.com). We anticipate that our model will continue to facilitate clinico-molecular insights across organ systems together with hypothesis generation for the testing of potential repurposing drug candidates, new pharmacological targets and clinically relevant biomarkers. Our work suggests that whole patient knowledge models of human disease can potentially expedite the development of new therapeutic strategies and support evidence-driven clinical hypothesis generation and decision making.

## 1 Introduction

**FIGURE 1:**
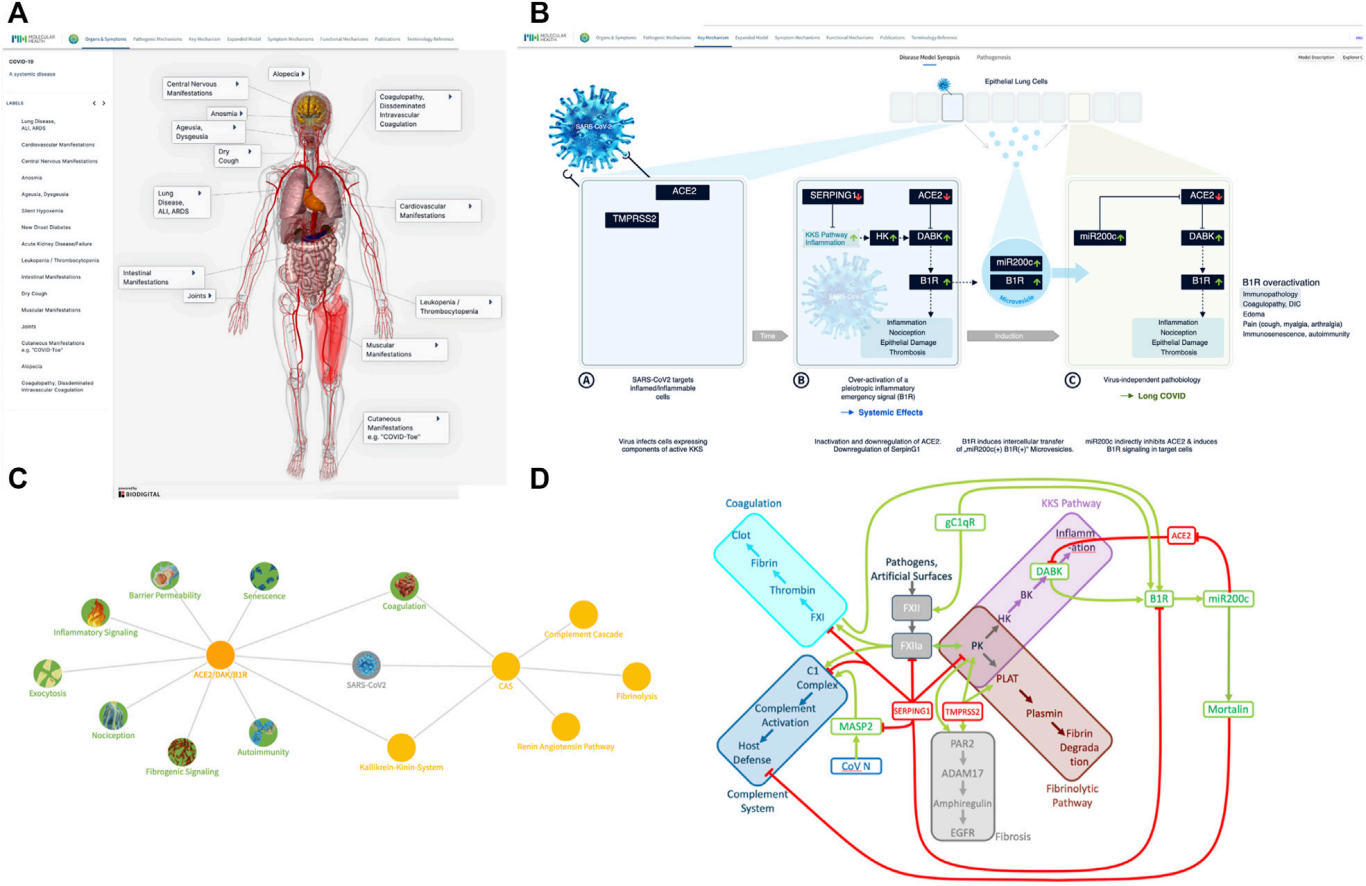
COVID-19 disease model—from symptomatology to molecular mechanisms. **(A)** The many faces of COVID-19—a systemic disease: COVID-19 comes with diverse clinical manifestations affecting a broad range of organ systems. Originally identified as respiratory disease (Huang et al., 2020) (pneumonia, acute respiratory distress syndrome (ARDS), Acute Lung Injury (ALI), shortness of breath, cough) with highly viscous mucus (Wu et al., 2020; Zhu et al., 2020), many more manifestations and affected organs were reported over time. These include neurologic manifestations (Mao R. et al., 2020; Ellul et al., 2020)and silent hypoxemia (Couzin-Frankel, 2020), musculoskeletal manifestations (Cipollaro et al., 2020), gastroenterological symptoms (Barrera et al., 2020; Mao Y. et al., 2020), cardiovascular manifestations (Kang et al., 2020; Zheng et al., 2020), endotheliitis (Varga et al., 2020), coagulopathy (Levi et al., 2020), new onset diabetes (Rubino et al., 2020), kidney injury (Selby et al., 2020), leukopenia (Ruan et al., 2020). Visualization was realized using the Biodigital 3D anatomy model (www.biodigital.com). **(B)** Synopsis of the basic COVID-19 Disease Model. **(B)** (sub-panel A) The virus targets host cells expressing ACE2 and TMPRSS2, active components of the KKS. **(B)** (sub-panel B) Within the cell the virus induces downregulation of SERPING1 and ACE2. SERPING1 downregulation induces activation of the CAS and KKS. ACE2 downregulation results in accumulation of DAKs. Excess DAKs activate B1R, triggering a constitutive activation and auto-amplification. Under normal conditions B1R activation triggers a fast, transient inflammatory emergency reaction inducing neutrophil and leukocyte recruitment and infiltration, opening of the epithelial/endothelial barriers for their transmigration, coagulation for local walling-off, fibrogenesis for wound healing, senescence as host defense mechanism and transient nociception. Constitutive activation of this system leads to excess inflammatory signaling, epithelial/endothelial barrier breakdown, induction of thrombosis, fibrosis, pain and other effects. **(B)** (sub-panel C) B1R signaling induces the formation of MVs bearing B1R and miR200c. These are transferred to target cells, inducing further expression of miR200c, which leads to ACE2 downregulation and formation of excess DAKs which signal *via* B1R. Thus, MV transfer *via* auto-activation and amplification of the B1R system may trigger the virus independent propagation of an inflammatory phenotype. **(C)** COVID-19 clinical phenotypes associated to eight key pathogenic mechanisms. Key mechanisms (green) and associated signs, symptoms and syndromes of COVID-19 (blue) induced by over-activation of the ACE2/DAK/B1R signaling axis. Members of the axis, as well as regulatory elements (e.g., miR200c, SIRT1, EZH2, etc.) converge at these mechanisms and complement each other to various degrees in inducing the corresponding phenotypes. **(D)** The key players of the seed model converge at the CAS, Contact Activation System. CAS constitutes a group of plasma proteins that are activated by FXII. FXII activates proinflammatory, procoagulant and host defense pathways. The initial step involves activation of PK, prekallikrein. PK reciprocally activates FXII. Inflammation results from the activation of the KKS, Kallikrein Kinin system resulting in the release of kinins, BK, bradykinin and KD, Kallidin, and the DAK, des-Arg-Kinins DABK and DAKD from high molecular weight kininogen (HK). Coagulation is driven by FXIIa‐mediated cleavage of FXI, which eventually leads to thrombin activation and clotting. FXII activation also leads to increased plasmin activation, which breaks down the fibrin clot. Finally, FXII can activate C1r and C1s of the complement pathway. SERPING1 is the key regulator of CAS activation. It is the main inhibitor of the FXIIa, the C1 complex and the conversion of HK to kinins. TMPRSS2 activates tissue kallikrein KLK2, PLAT and fibrinogenic PAR2, which can also be activated by KK. ACE2 metabolizes DAK, thus inhibiting DAK signaling. DAK activates and induces expression of B1R. gC1qR, a pathogen receptor and key activator of the CAS, induces the expression of B1R. B1R activation in turn induces miR200c, which directly targets and suppresses ACE2, constituting a feed-forward loop leading to the generation of excess DAK. miR200c induces Mortalin an inhibitor of the cytotoxic component of the complement cascade. Complement activator MASP2 is a target of the virus N protein. SERPING1 inhibits MASP2 proteolytic activity.

Beginning in early 2020, the COVID-19 pandemic has paralyzed the world, with over half a billion infections and >5 million deaths reported by mid-2022 (de Seabra Rodrigues Dias et al., 2022). A multiform systemic disease, COVID-19 presents a multitude of clinical phenotypes afflicting multiple organs and manifesting from mild symptomatology to critical illness (Figure 1A). However, the current knowledge landscape remains both disjointed and diverse, precluding a holistic understanding of COVID-19’s complex pathogenesis and associated “long COVID” syndromes. To address this challenge, global holistic clinico-molecular data-mining platforms capable of elaborating whole-patient mechanistic knowledge models are required, to expedite and provide more advanced disease comprehension in response to emergent global health emergencies such as the COVID-19 pandemic.

Recently, we utilized a precision medicine data and technology platform (Dataome), that has previously been validated in clinical decision support and in the elucidation of novel molecular mechanisms associated with drug efficacy and safety (Armaiz-Pena et al., 2013; Pradeep et al., 2015; Schell et al., 2016; Bohnert et al., 2017; Soldatos et al., 2018; Soldatos and Jackson, 2019; Schotland et al., 2021), to deliver a digital whole patient COVID-19 symptomatology model to the community (“*COVID-19 Explorer*”; at https://covid19.molecularhealth.com) (Brock et al., 2022). Here, we demonstrated that it is possible to structure and logically connect diverse clinical and molecular features of COVID-19 pathobiology by using digital health platforms like the Dataome technology platform. Dataome provided a knowledge and data-mining infrastructure that allowed us to rapidly initiate an iterative computer-augmented modeling approach, guided by disease modeling experts that allowed us to build a comprehensive digital model during the early phases of the pandemic (Brock et al., 2022).

In this work, we explore the utility and importance of the holistic patient-level knowledge model within the *COVID-19 Explorer* resource and examine its key features, particularly surrounding the molecular underpinnings of COVID-19 symptomatology. Characteristically, the model suggests that the multitude and complexity of observed, and seemingly disparate COVID-19 clinical phenotypes may be linked to the pleiotropic activity of eight core molecular mechanisms involved in the host response. Moreover, the model revealed functionally connected mechanisms across multiple organ systems and identified novel hypotheses for both viral dependent and independent disease mechanisms. Here, we discuss these molecular perspectives in detail, including the content of the causative pathogenic mechanisms underlying COVID-19 phenotypes, real world confirmatory observations, and examples that demonstrate some of the key analytical utilities (e.g., risk factors). Finally, we focus on the detection of potentially new (or unobvious) clinico-molecular insights across organ-systems and on the identification of potential pharmacologic targets against COVID-19 (Brock et al., 2022).

Our results show that structuring emergent molecular knowledge *via* a pan-symptomatic disease format, helped develop a comprehensive whole patient COVID-19 knowledge model that could expedite evidence-driven hypothesis generation and the discovery of novel clinico-molecular insights. Inspired by the effectiveness of this strategy, we propose that whole-patient knowledge modeling of systemic symptomatology for any disease, as opposed to traditional pathway-specific modeling, may provide important advantages in tackling current unmet medical needs and future health emergencies. Importantly, the whole-patient knowledge model is provided to the community in the form of both an open-source web-sever (found at https://covid19.molecularhealth.com), and a tabular COVID-19 “Cockpit” (Brock et al., 2022). Together they are aimed at enabling a variety of use-case scenarios useful to support translational and clinical researchers in hypothesis generation and the development of new diagnostic and therapeutic strategies.

## 2 Materials and methods

### 2.1 Knowledge capture, integration and modelling using the Dataome technology

Our studies were led by a small team of disease modelling experts working with the Dataome Technology Platform to capture, structure and logically connect diverse clinical and molecular features of COVID-19 pathobiology. Dataome consists of three primary modules that enable the constant 1) capture and curation 2) integration and connection and 3) analysis of, globally available data sources of clinical and molecular knowledge (Supplementary Material S1, Figure 1):

The *Dataome Capture* technology uses an ensemble of public/proprietary algorithms and resources to empower the global harvesting, quality assurance and integration of emergent clinical and molecular data. Curated and quality-controlled data is then integrated into the *Data Nucleus.*

The *Dataome Nucleus* encompasses data from >100 public, commercial and proprietary developed resources. Datasets span a broad range of content, size and formats—from more general, such as literature, biomedical ontologies or information on proteins and genes, drugs and their targets, interactors, bio-molecular pathways, and interactions. Other resources include data on millions of patients from diverse real-world data (RWD) databases. Moreover, Dataome contains structured information regarding therapeutic guidelines and variant classification, as well as curated datasets pertaining to clinical biomarker interpretation, pathway/interaction relationships, drug and clinical trial information.

The extensive data input contained within the *Nucleus* provides the evidence-base, required by the analytical technologies and AI-based tools contained within the *Dataome Analytics* part of the technology. These software tools also exist in specialized analytical pipelines that integrate bioinformatics, chemo-informatics, systems biology, clinical data science, and AI/machine learning (integrated analytics, feature engineering and powerful pre-trained models) based methodologies.

### 2.2 Knowledge modeling workflow

Using these components, the *Dataome Nucleus* was queried with 332 high confidence human protein interactors with the SARS-CoV-2 proteome^1^, to provide systematic expansion inclusive of associated pathways, interactors and regulatory elements yielding a massive interactome network mapping the majority of the entire human proteome. To optimize the specificity of analysis, we focused initial modeling on the molecular determinants of host cells that define them as viral targets and the immediate impact of viral infection on the host cell response. Next, we linked key molecular protagonists to COVID-19 symptomatology, outcome and severity associated risk-factors. These factors were mapped onto pathways, interactors and/or substrates and regulatory networks exploiting interaction databases and data from literature within the *Dataome Nucleus*. Pathways were defined through entities as canonical elements (ACE2, SERPING1) or *via* their substrates (TMPRSS2) and converged into a common pleiotropic signaling cascade, the contact activation system. The *Dataome Nucleus* was also screened for all factors and associated phenotypes linked to naturally occurring variants or functional studies involving pharmacological perturbations or knock-out models, with an initial focus on COVID-19 pulmonary phenotypes.

**FIGURE 2:**
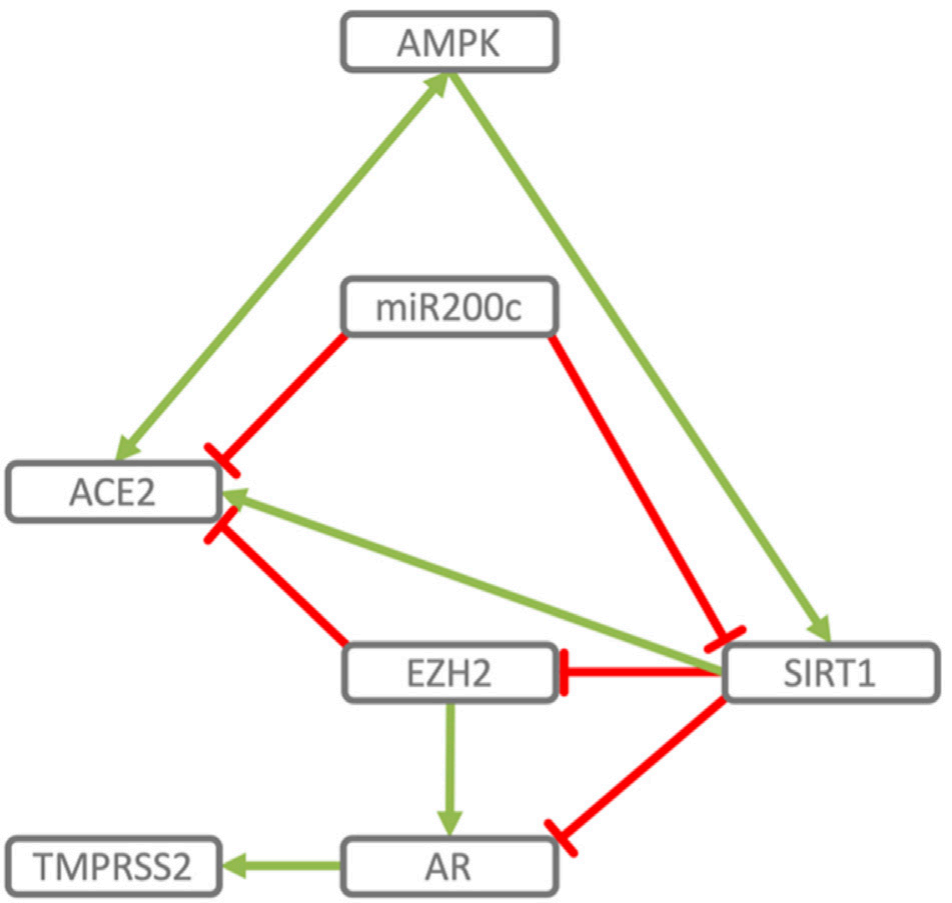
Reciprocal regulation of ACE2 and TMPRSS2. ACE2 and TMPRSS2 are reciprocally regulated by miR200c, SIRT1 and EZH2. SIRT1 induces ACE2 expression (Clarke et al., 2014; Yacoub et al., 2014; Moran et al., 2017; Shao et al., 2019) and indirectly represses TMPRSS2 by acting as co-repressor of the AR, androgen receptor (Dai et al., 2007) and by repressing EZH2 (Lu et al., 2011). EZH2 represses ACE2 (Li Y. et al., 2020) and, in an androgen receptor dependent manner, derepresses TMPRSS2 as well as its substrate KLK2 (Kim et al., 2018). miR200c directly represses ACE2 (Liu et al., 2017) and SIRT1 (Carlomosti et al., 2017), through which it may indirectly induce TMPRSS2. AMPK activation increases phosphorylation and stability of ACE2 (Zhang et al., 2018) and *via* SIRT1 induces expression of ACE2 (Clarke et al., 2014). In turn ACE2 activates AMPK (Murça et al., 2012).

Readouts from these *in silico* analyses were organized by experts into molecular models containing salient information for each phenotype. The resulting “base model” was then matched to molecular models of diseases that are symptomatically related to specific manifestations of COVID-19 (see Supplementary Material S1, Figure 2). At each stage of the disease/symptom modelling process, the extracted “premodels” were inspected and remodeled in PathVisio (Version: PathVisio 3.2.2). During the remodeling, relevant “premodel” references were attached to the respective objects in PathVisio and manually complemented as necessary. Entities and relationships associated with each mechanism/symptom were presented by a JavaScript-animated SVG image. Graphical renditions were produced *via* the PathVisio program, with data outputs in GPML and SVG format. The web application displaying these “submodels” is served by a Flask micro web framework. The application presents the SVG for a sub-model, animated by the d3.js JavaScript library. To project a most actual and comprehensive information, the collection of citations supporting the relations of each “submodel,” range from peer-reviewed articles, very recent conference content (e.g., abstracts) to *ad hoc* communications and is provided as auxiliary information *via* respective animation components.

COVID-19 explorer and data availability: The final comprehensive model, linking key molecular mechanisms to COVID-19 symptomatology and the related source data, is made publicly available *via* a web-based COVID-19 Explorer (http://covid19.molecularhealth.com)**.** For detailed information on data availability please see Supplementary Material.

## 3 Results

### 3.1 Insights and hypotheses from the COVID-19 knowledge model

The comprehensive and fully interactive COVID-19 disease model (aka the COVID-19 Explorer, accessible at: https://covid19.molecularhealth.com), provides a link between possible molecular disease mechanisms [aberrant contact activation system (CAS) and ACE2/DAK/B1R signaling] and eight core pathogenic processes: inflammatory signaling, coagulation, barrier permeability, senescence, autoimmunity, fibrogenic signaling, nociception and exocytosis. These mechanisms are in addition linked with respective symptoms, associated pathogenic pathways and affected organ-systems.

### 3.2 Host factors mediating SARS-CoV-2 infection

Analysis of the COVID-19 knowledge model revealed a converging molecular network, delineating host-factor responses to SARS-CoV-2 *via* the host proteins responsible for virus entry, together with downregulated components of Interferon Stimulated Genes (ISG’s) induced by virus infection. SARS-CoV-2 cell-entry depends on binding of the viral spike (S) protein to the cellular receptor ACE2 and S protein priming by the host cell serine protease TMPRSS2 (Hoffmann et al., 2020) (Figure1B). SARS-CoV-2 targets diverse cell types within the lung, all of which express ACE2 (Hamming et al., 2004; Jia et al., 2005; Xu et al., 2020). TMPRSS2 is highly expressed with broader tissue distribution, suggesting that ACE2 expression may be limiting in cellular susceptibility to infection (Sungnak et al., 2020). The induction of ISG expression is part of the early response to viral infections. Analysis of ISG expression in human airway epithelial cells reveals the ability of SARS-CoV to interfere with this process and avoid anti-viral host-response (Menachery et al., 2014). Induction of ISGs is delayed and occurs after peak titers, a behavior also confirmed for SARS-CoV-2 (Nienhold et al., 2020). While ISG expression is universally increased, ACE2 and SERPING1 are significantly downregulated (Menachery et al., 2014) (Figure 1B). As SERPING1 is one of the proteins with the highest connectivity in the SARS-CoV-1 and SARS-CoV-2 interactomes, it was proposed that SARS-CoV-2 infection directly causes deficiency in C1 esterase inhibitor (Thomson et al., 2020). Recently, clinical samples of COVID-19 patients revealed C1-Inhibitor as one of the most prominently downregulated genes with 80-fold decreased expression (Mast et al., 2021).

**FIGURE 3:**
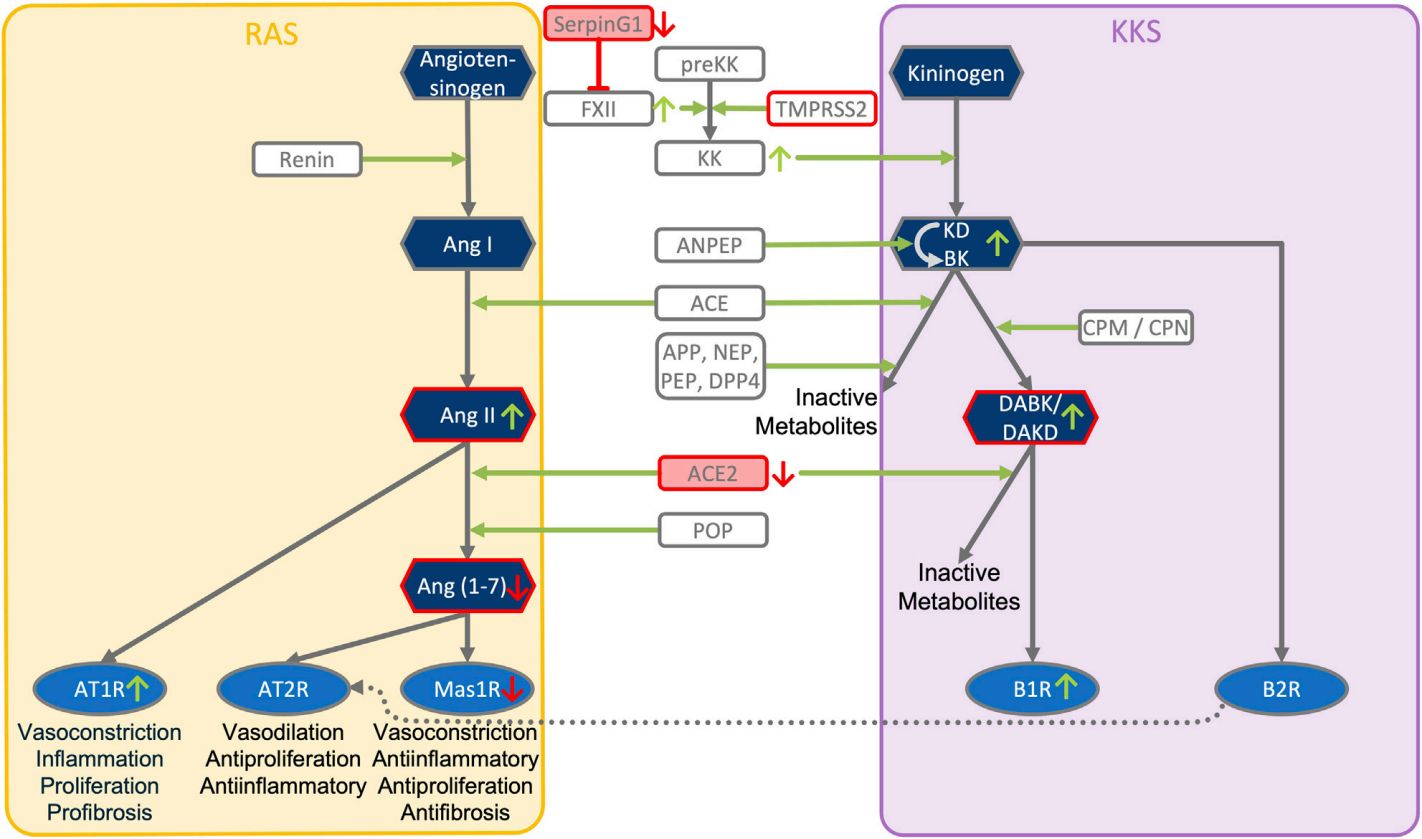
ACE2, SERPING1 and ACE2 converge at the crosstalk between KKS and RAS pathways. The KKS comprises the kininogens, the kallikreins, the kinins and the kinin receptors. KK, Kallikrein is generated from preKK, prekallikrein by factor XIIa during activation of CAS. FXIIa can be inhibited by SERPING1 of the complement system. Kinins are generated by cleavage of kininogens by kallikreins. Kinins are BK, bradykinin and Lys-BK (Kallidin, KD) which both activate B2R, and their active des-Arg metabolites, generated by CPM/N, des-Arg (Brock et al., 2022)-BK (DABK) and des-Arg (Hoffmann et al., 2020)-KD (DAKD), which activate B1R. In this figure, the RAS, renin-angiotensin system has been reduced to key components comprising the angiotensins (AngI, II, 1–7), the angiotensin receptors (AT1R, AT2R, Mas receptor) and the ACEs, angiotensin converting enzymes. KKS and RAS are mainly connected by ACE and ACE2. ACE (kininase II) inactivates the kinins and generates angiotensin II (AngII). ACE2 inactivates the B1R agonist DAK, des-Arg kinins and also metabolizes AngII into the Ang-one to seven agonist of AT2R and Mas oncogene. Systemic AngII conversion to Ang (1–7) depends on POP, Prolyloligopeptidase and is ACE2 independent. APP, Aminopeptidase P; NEP, Neprilysin; PEP, Prolylendopeptidase inactivate kinins. AT2R directly interacts with B2R through heterodimerization and AT2R overexpression increases KK activity. Simultaneous downregulation of SERPING1 and ACE2 leads to accumulation of excess DAK (DABK and DAKD).

**FIGURE 4:**
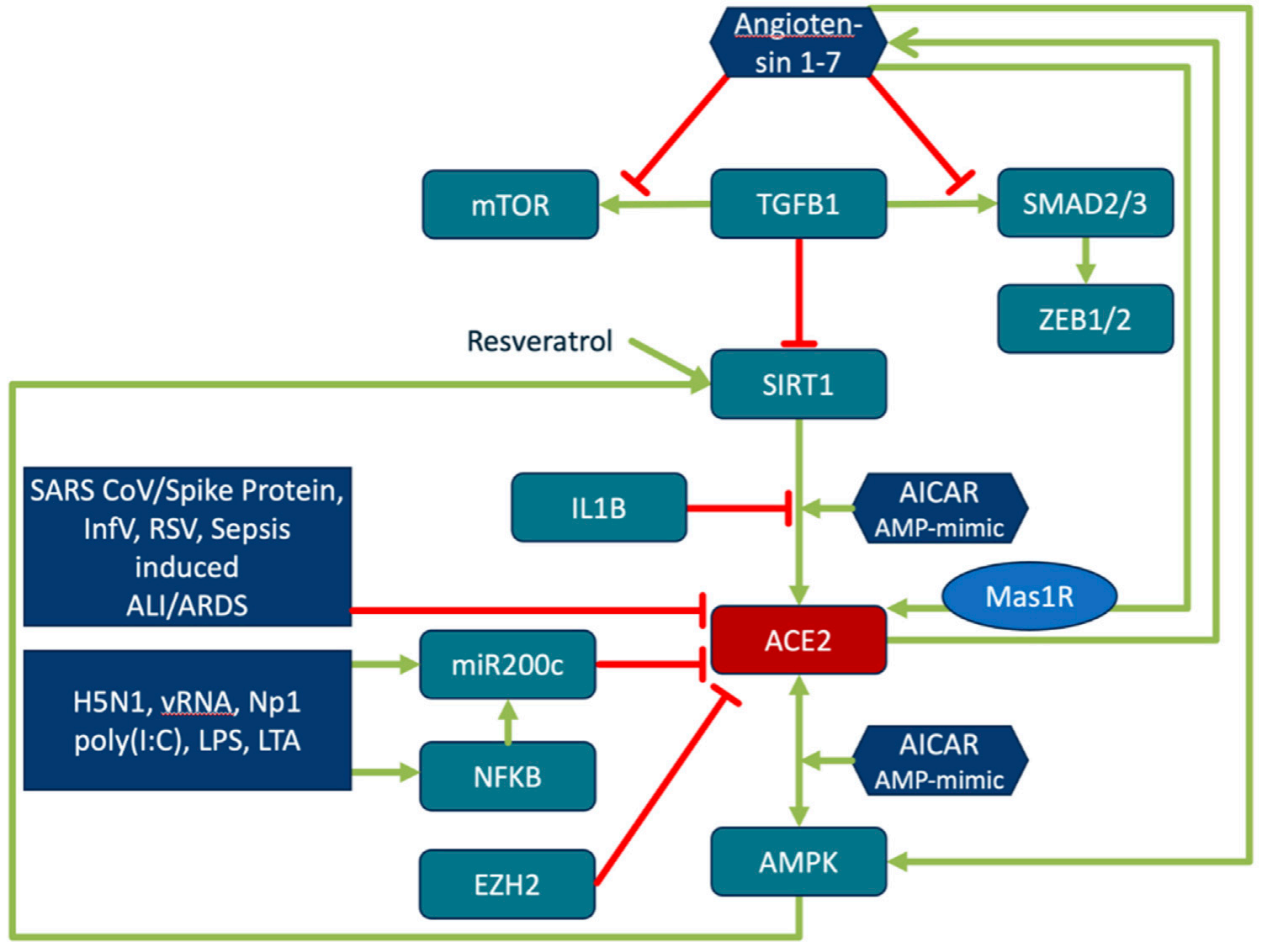
Regulation of ACE2 expression and its activity. Regulation of ACE2 is closely interwoven with energy sensing, inflammatory processes and ageing. TGFβ downregulates ACE2 expression in a SIRT1-dependent manner. SIRT1 activator, SRT1720, restores the ACE2 expression. ACE2/Ang (1–7)/MasR are decreased in pulmonary fibrosis and negatively correlate with TGFβ expression. Ang (1–7) directly inhibits TGFβ-induced phosphorylation of SMAD2 and SMAD3 and mTOR, and suppresses the expression of the downstream target genes of TGFβ/SMAD signaling (ZEB1, ZEB2, TWIST, and SNAIL1) (Shao et al., 2019). Activation of the energy status sensing AMPK by AMP mimic AICAR increases phosphorylation of ACE2 and SIRT1 dependent expression of ACE2. Phosphorylation of ACE2 enhances its stability and activity (Zhang et al., 2018). ACE2 in turn activates AMPK. Phosphorylation of AMPK is reduced in ACE2 knock-out, while Ang (1–7) activates AMPK. Resveratrol and Ang (1–7) increase ACE2 expression. SIRT1 antagonist and Mas antagonist block this effect (Liu Y. et al., 2019). SIRT1 binds to the ACE2 promoter. Binding is increased by AICAR treatment and decreased by IL1β (Clarke et al., 2014). ACE2 activation by SIRT1 leads to increased levels of Ang (1–7) (Yacoub et al., 2014). The compound Resveratrol induces SIRT1-dependent upregulation of ACE2 (Moran et al., 2017). The role of miR200c in this context is explained in the sections on miR200c function and the reciprocal regulation of ACE2 and TMPRSS2.

**FIGURE 5:**
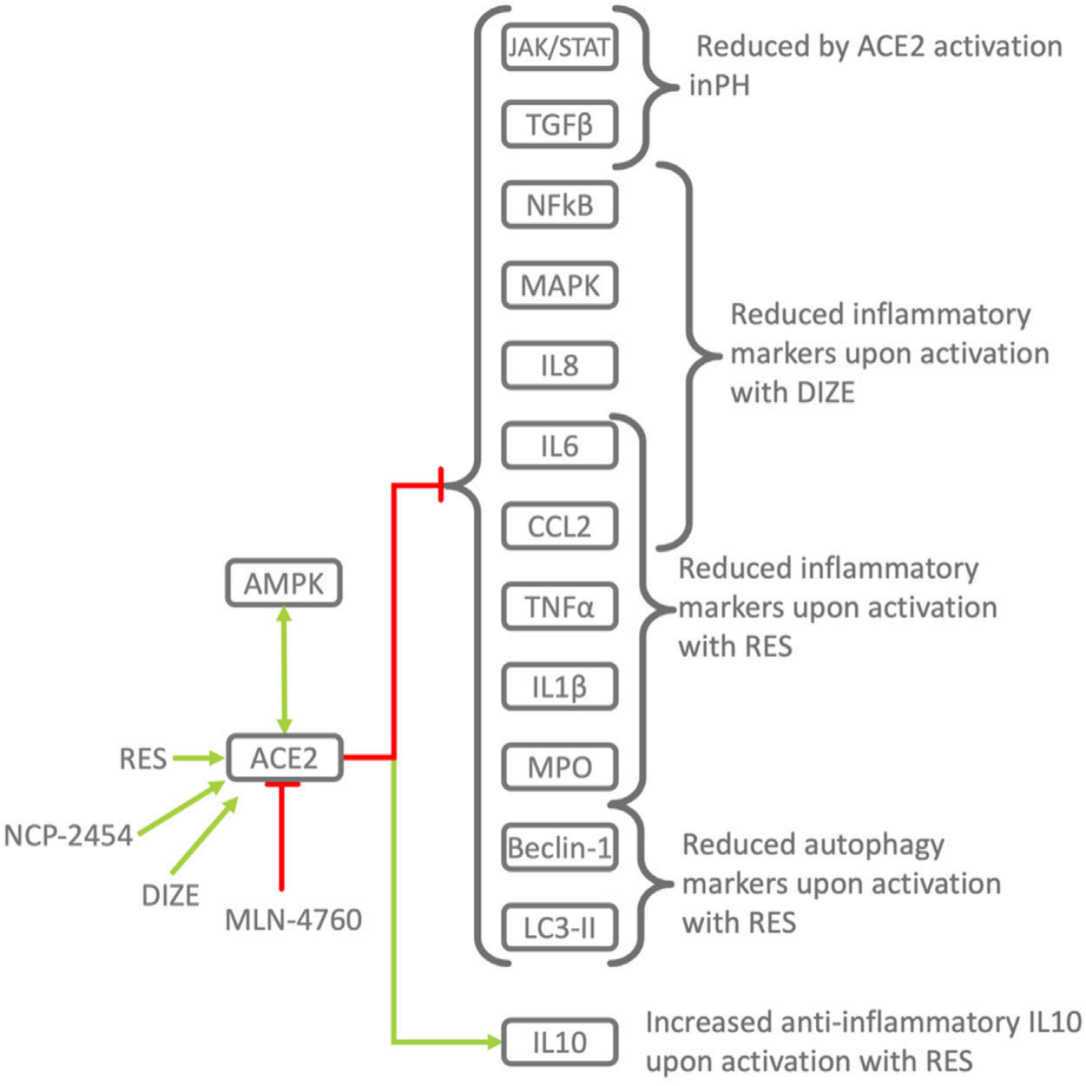
Pharmacological activation/inhibition of ACE2 demonstrates its protective role in inflammation. RES, Resorcinolnaphthalein activates ACE2 in a dose-dependent manner (Hernández Prada et al., 2008) and leads to decrease in pro-inflammatory TNFα, CCL2, IL6 and increase in anti-inflammatory IL10 improving endothelial dysfunction in PH (Li et al., 2012). Activation of ACE2 by RES alleviates the severity of ALI the lung, while ACE2 downregulation and pharmacological inhibition leads to the opposite effect. Similar mechanisms were observed in other diseases. Phosphorylation of ACE2 by AMPK in the endothelium leads to its activation and mitigates PH, pulmonary hypertension (Zhang et al., 2018). In animal models of PH, activation of ACE2 by the RAS, renin-angiotensin system improves endothelia-dependent vasorelaxation, decreases proinflammatory TNFα, CCL2, IL6 and increases anti-inflammatory IL10 (Li et al., 2012) and ACE2 activator reduces monocrotaline-induced PH by suppressing the JAK/STAT and TGFβ (Haga et al., 2015).

The model indicates that the viral cell-entry mechanism and disease-specific ISG signature provides three key active components of SARS-CoV infected cells (TMPRSS2, ACE2 and SERPING1), which may functionally converge in the same pleiotropic signaling systems, namely the Contact Activation System (CAS) and Kallikrein Kinin System (KKS) pleiotropic signaling (Figure 1D):

• TMPRSS2 and ACE2 through common elements of their regulatory network (miR200c, SIRT1, EZH2 and AMPK) are regulated in a reciprocal manner (Figure 2).
• ACE2 is co-expressed with TMPRSS2 in respiratory tract cells and oral mucosa (Hamming et al., 2004; Jia et al., 2005; Xu et al., 2020; Ziegler et al., 2020) and present in endothelial cells and in the arterial smooth muscle cells of many organs (Hamming et al., 2004). As part of ISG-response, ACE2 plays a tissue-protective role in innate immunity (Menachery et al., 2014; Ziegler et al., 2020). SARS-CoV infection leads to robust down‐regulation of ACE2 RNA and protein expression (Kuba et al., 2005; Menachery et al., 2014). Loss of pulmonary ACE2 is a key event in the molecular pathogenesis of acute lung injury (ALI) (Imai et al., 2005; Kuba et al., 2005; Kuba et al., 2006; Imai et al., 2008). The detrimental effect of ACE2 downregulation in SARS-CoV infection is attributed to its role in the Renin-Angiotensin System (RAS) (Tseng et al., 2020). ACE2 converts AngII to Ang (1–7). Its downregulation leads to over-activation of the AngII receptor AT1R (Jia et al., 2005; Banu et al., 2020) (Figures 3, 4). However, on a systemic level, this conversion is ACE2-independent (Serfozo et al., 2020). ACE2 also participates in the KKS. It efficiently inactivates des-Arg-kinins (DAK) (Vickers et al., 2002). Moreover, pharmacological activation and inhibition of ACE2 emphasise it is critical role in inflammation (Figure 5). Thus, our model implicates ACE2 in the context of the KKS, but also in the context of RAS.
• While TMPRSS2 plays a key role in prostate cancer (Tomlins et al., 2005; Kron et al., 2017), it also activates pro-kallikrein-2 (KLK2) (Lucas et al., 2014), resulting in selective cleavage of kininogen to release kallidin (KD), a precursor of the ACE2 substrate des-Arg (Hoffmann et al., 2020)-KD (DAKD). Thus, TMPRSS2 is an activator of the KKS.

SERPING1 is the major regulator of the CAS (Figure 1D). Downregulation of SERPING1 leads to activation of the CAS and excess Bradykinin (Cugno et al., 2009), a precursor of the ACE2 substrate DABK. Aberrant regulation of CAS leads to inflammation and autoimmunity and is involved in diseases like ischemia/reperfusion-syndrome, sepsis, atherosclerosis and diabetes (Davis et al., 2010; Schoenfeld et al., 2016).

### 3.3 Host-response driven disease mechanisms

Global level analysis of the model reveals that the SARS-CoV-2 virus targets cells expressing constituents of a highly inducible inflammatory signaling system leading to its excess activation. The model further suggests that it is the pleiotropic nature of this system that appears to be responsible for the diverse clinical manifestations of COVID-19. Importantly, it also provides a possible mechanism through which a disease phenotype may be propagated, even in the absence of the original viral trigger (Figure1B). The following sections analyze these different perspectives in detail.

#### 3.3.1 Convergence within the pleiotropic KKS dysregulates ACE2-DAK-B1R axis: A “perfect storm,” triggering systemic disease

The functions of ACE2, TMPRSS2 and SERPING1 converge within the KKS. Key products of KKS, the kinins bradykinin (BK), Kalladin (KD), and their des-Arg metabolites, des-Arg (Brock et al., 2022)-BK (DABK) and DAKD are major inflammatory mediators. By inactivating DABK and DAKD, ACE2 is a negative regulator of KKS signaling (Jia, 2016). Under normal conditions, DABK and DAKD are readily metabolized (Vickers et al., 2002). Under inflammatory conditions, DABK can accumulate (McLean et al., 2000), with downregulated ACE2 leading to further accumulation of des-Arg-Kinins (DAK) (Sodhi et al., 2018). Thus, induction of CAS along with downregulation of KKS inhibitor SERPING1 and des-Arg-Kinin inactivator ACE2 may lead to local excess of DAK, resulting in constitutive activation and induction of its receptor B1R (Figure 1B). Indeed, clinical samples from COVID-19 patients revealed that B1R was one of the most prominently upregulated genes with a 260-fold expression increase (Mast et al., 2021).

B1R is part of a rapid response system expressed in inflammation associated cytotypes (Walker et al., 1995; Böckmann and Paegelow, 2000; Wu et al., 2002). It is controlled by inflammatory stimuli, such as IL1β or availability of DABK or DAKD, and rapidly switched-off by internalization in the absence of ligand (Marceau et al., 1983; Dray and Perkins, 1993; Couture et al., 2001; Prado et al., 2002; Calixto et al., 2004; Leeb-Lundberg et al., 2005). Spatial proximity of B1R to enzymes controlling the half-life of its ligands contributes to its regulatory role (Lamb et al., 2002; Lu et al., 2008; Qadri and BaderKinin, 2018). B1R activation promotes cytosolic influx of extracellular Ca^2+^. Sustained activation increases receptor expression and induces resistance to desensitization and internalization, triggering a positive feed-forward-loop and excess downstream signaling (Faussner et al., 1999; Marceau et al., 2002; Calixto et al., 2004). B1R is involved in signaling cascades controlling numerous mechanisms that when over-activated are directly related to the molecular pathologies underlying COVID-19. Characteristically, it induces 1) an inflammatory response at the site of infection and recruitment of neutrophiles and leukocytes, 2) coagulation to prevent infection, 3) nociception, 4) increased endothelial/epithelial barrier permeability allowing fluid and proteins to move into the interstitium and immune cells to trans-migrate, 5) reduced exocytosis, 6) senescence as mechanisms to fight pathogens and 7) fibrogenesis for wound healing (Figure 1C).

#### 3.3.2 A feed-forward loop decoupling molecular pathogenesis from virus load: Auto-induction of B1R bearing MVs and interplay with the regulatory miR200c

**FIGURE 6:**
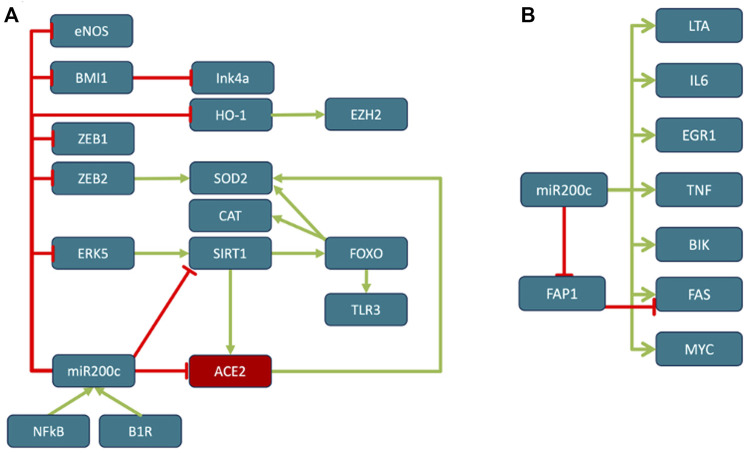
Role of miR200c. **(A)** miR200c down-regulates ACE2, reactive oxygen species (ROS) scavengers, eNOS, SIRT1 and derepresses senescence marker p16INK4a. miR200c levels elevated in lung diseases such as fibrosis, chronic obstructive pulmonary disease (COPD) and pneumonia (Cao et al., 2014; Jiang et al., 2017; Liu et al., 2017). B1R activation induces miR200c (Tang et al., 2017), which in turn represses ACE2 in acute respiratory distress syndrome (ARDS) (Liu et al., 2017). miR200c directly represses SIRT1 (Carlomosti et al., 2017), eNOS and indirectly FOXO1 (Carlomosti et al., 2017), which in turn leads to downregulation of ROS scavengers Catalase and SOD2. In addition, oxidative stress induced miR200c downregulates ROS scavenger heme oxidase HO1 (Wu et al., 2017). ROS scavengers alleviate ARDS and septic shock (Janssen and Nozik-Grayck, 2017), while when down-regulated, increased ROS leads to ARDS progression and endothelial/epithelial barrier dysfunction (Kellner et al., 2017; Chen et al., 2018). In addition, HO1 stimulates the expression of EZH2 (He et al., 2019) (see reciprocal regulation of ACE2 and TMPRSS2). Downregulation of SOD2 and SIRT1 by miR200c is mediated through ZEB2 and ERK5 (Wu et al., 2017). FOXO is activated in patients with respiratory tract diseases. TLR3-mediated innate immune responses of bronchial epithelial cells depend on FOXO and its deficiency results in suppression of epithelial innate immune function and increased of pathogen uptake (Totura et al., 2015). miR200c also targets BMI-1 (Cao et al., 2011), absence of which severely affects lymphopoiesis (van der Lugt et al., 1994) (see senescence). **(B)** The effects of miR200c overexpression in epithelial cells. Overexpression induces LTA (19,5x), IL6 (17,5x), EGR1 (8,4x), TNF (7,2x), BIK (4,8x), FAS (4,2x), MYC (2, 8) (Tryndyak et al., 2010). Lymphotoxin (LTA)-signaling participates in airways remodeling during inflammation (Koroleva et al., 2018). Notably, SARS-CoV triggers EGR1 dependent activation of TGFß inducing profibrotic responses (Li et al., 2016), while inhibition of EGR1 ameliorates pulmonary fibrosis (Bhattacharyya et al., 2013). BIK may contribute to lung destruction in COPD (Joyce-Brady and Tuder, 2011) and MYC is a key regulator in sepsis-induced ARDS (Zhang et al., 2019). FAS induces apoptosis. In addition to upregulating FAS, mir200c directly targeting FAS inhibitor FAP1 (Schickel et al., 2010). HCV induced miR200c down modulates FAP-1 resulting in significant increases in expression of collagen and fibroblast growth factor (Ramachandran et al., 2013).

Our patient-level model highlights that in B1R-mediated pathologies, intercellular information exchange *via* MVs may contribute to the dissemination of disease phenotypes (Figure 6). In acute vasculitis, B1R bearing MVs play a role in the bi-directional communication between leukocytes and endothelium. Here, MVs transfer functional receptors to promote kinin-associated inflammation (Kahn et al., 2017; Mossberg et al., 2017). Patients with acute vasculitis show high-levels of circulating B1R-positive endothelial MVs, which induce a neutrophil chemotactic effect. Patient plasma induces release of more B1R-positive MVs from endothelial cells. SERPING1 depleted plasma promotes excessive release of B1R-positive endothelial MVs, while addition of SERPING1 or B1R-antagonist abolishes this effect (Mossberg et al., 2017). Thus, B1R signaling induces excretion of B1R bearing MVs in an auto-amplifying manner, while downregulation of SERPING1 *via* induction of KKS increases ligand supply. Such MV-based intercellular communication also occurs between different airway cells (Gupta et al., 2019). Interestingly, airway epithelial cell secretions are enriched with components of the CAS and KKS, containing key regulators of these systems, indicating that under normal conditions this mechanism constitutes a well-balanced system. This aligns with the observation that pulmonary epithelial cells express active surface peptidases to degrade kinins rapidly, keeping the system balanced and protecting the lung (Ghebrehiwet et al., 2019).

**FIGURE 7:**
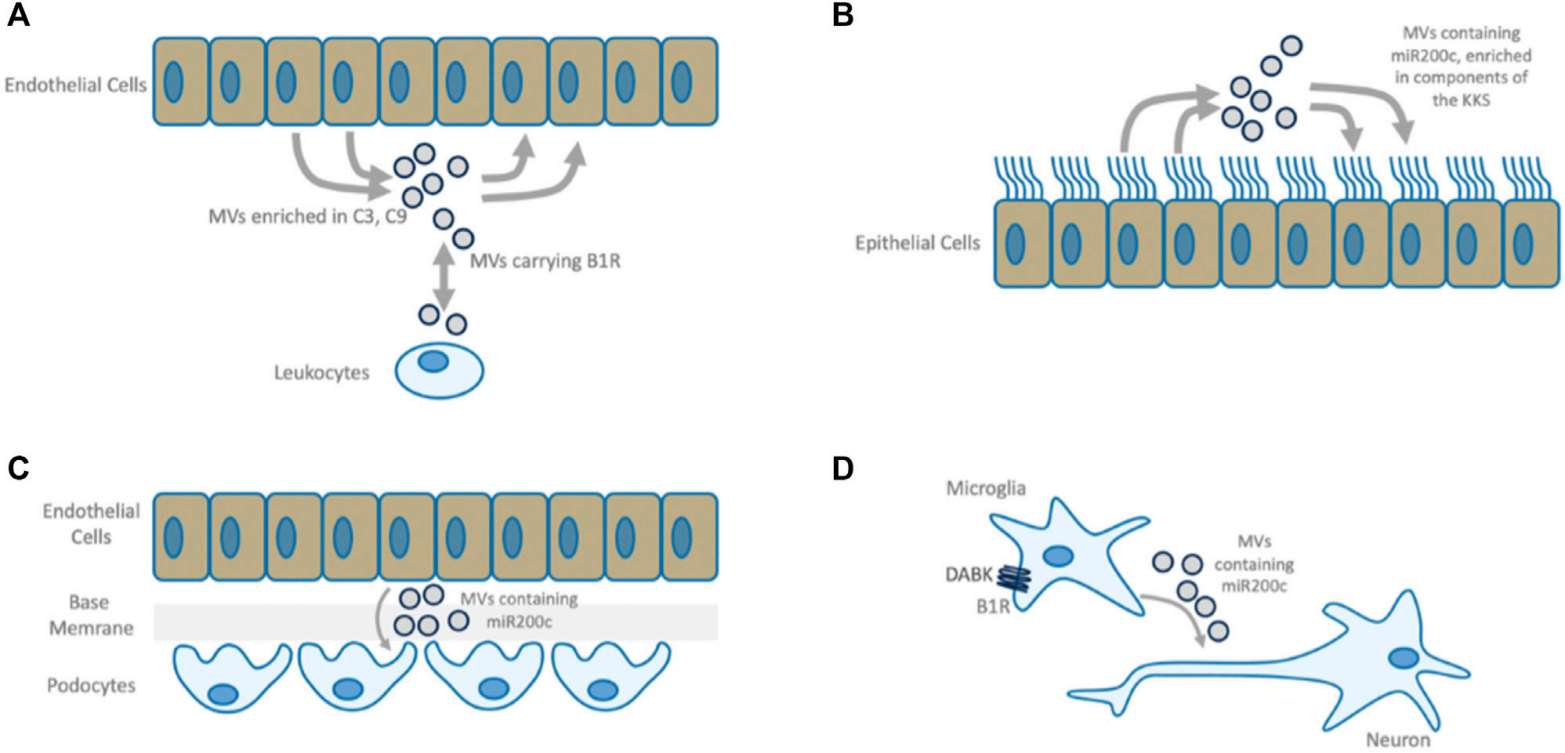
B1R mediated MVs. **(A)** B1R bearing microvesicles (MVs) participate in bi-directional communication between leukocytes and endothelium. Plasma from vasculitis patients is enriched in C3- and C9-positive endothelial MVs which can be reversed by B1R-antagonism or by supplementation of SERPING1 (Lopatko Fagerström et al., 2019). **(B)** MV-based intercellular communication between airway epithelial cells. Airway epithelial cell secretions are enriched with components of the CAS and KKS and contain both positive and negative regulators of these systems, indicating that under normal conditions this mechanism constitutes a well-balanced communication system. This is in line with the observation that pulmonary epithelial cells express active peptidases along their surfaces to degrade kinins rapidly, keeping the system in balance and protecting the lung from edema (Ghebrehiwet et al., 2019). These MVs also carry miR200c. **(C)** In Kidney Disease, activated endothelial cells cause functional changes to podocytes *via* MV transfer, causing endothelial barrier breakdown. miR200c bearing MVs are transferred in response to inflammatory mediators and induce mitochondrial stress, decrease VEGF secretion and increase expression of miR200c in target cells leading to podocyte dysfunction (Hill et al., 2020). Antagomir treatment ameliorates this disease phenotype (Ottaviani et al., 2019). Endothelial cell activation by the same inflammatory mediator induces epithelial barrier damage, nephrotic-range proteinuria, renal epithelial cell damage, infiltration of mononuclear leukocytes, and apoptosis of several renal cell types (Moreno-Manzano et al., 2003), a phenotype that is similar to B1R activation. **(D)** miR200c bearing MVs transferred from microglia to neural cells upon B1R activation mediate neuronal damage. B1R antagonism improves the neurologic function and causes downregulation of both miR200c in microglial cells and the quantity of MVs released microglial cells. MVs from miR200c knockdown cells have the same effect on neuronal cells as MVs derived from cells treated with B1R antagonist (Tang et al., 2017).

The secretome and exosomes of airway epithelial cells also contain miRNAs. miR200c, a negative regulator of ACE2 expression (Figure 6), is significantly enriched (Gupta et al., 2019) and induced by oxidative stress (Wu et al., 2017) and highly elevated in pneumonia and chronic obstructive pulmonary disease patients (Liu et al., 2017) and positively correlated with disease severity in interstitial lung disease (Jiang et al., 2017). In SARS-CoV, ALI or Acute Respiratory Distress Syndrome (ARDS) is associated with downregulation of ACE2, mediated by increased miR200c. Inhibition of miR200c ameliorates ALI (Liu et al., 2017). By targeting antioxidant proteins, miR200c leads to an increase in reactive oxygen species (ROS) formation (Carlomosti et al., 2017; Wu et al., 2017), contributing to progression of the inflammatory process and endothelial/epithelial barrier dysfunction (Kellner et al., 2017). miR200c has also been shown to directly target FOXO (Carlomosti et al., 2017). FOXO is activated in patients with pulmonary diseases and its deficiency results in suppression of TLR3-dependent epithelial innate immune function and increased pathogen uptake (Totura et al., 2015). In epithelial cells, miR200c overexpression strongly induces pro-inflammatory IL6 (Tryndyak et al., 2010). miR200c and B1R carrying MVs play a role in the molecular pathology of many diseases, including kidney disease, vasculitis and Kawasaki Disease (KWD) (Zhang R. et al., 2017) (Figure 7). Next, MVs secreted by stressed cardiomyocytes are highly enriched in miR200c. MV-mediated intercellular communication between microglial and neural cells in cerebral injury (Tang et al., 2017) suggests that B1R signaling is involved in the generation of miR200c carrying MVs. Here, miR200c bearing MVs are transferred from microglia to neural cells mediating neuronal damage. In this context, miR200c selectively represses Syntaxin1, an important functional protein for neurotransmission causing loss of function of neural cells (Davis et al., 1998; Watanabe et al., 2013). Interestingly, these pathological processes can be ameliorated by B1R antagonism or SERPING1 supplementation mimicking the phenotype of Kininogen knockouts.

In summary, the model shows that B1R and miR200c bearing MVs may play an important role in molecular pathologies associated with epithelial and endothelial barrier damage, fibrosis, cardiac and neuronal damage: MVs containing miR200c are involved in communication between airway cells, B1R activation leads to self-propagating dissemination of B1R bearing MVs and can induce the formation of miR200c positive MVs. In targeted cells, B1R is auto-induced and miR200c can suppress ACE2, which in turn leads to accumulation of B1R ligands DAK. This constitutes a mechanism through which a disease phenotype may be propagated even in the absence of the original viral trigger. Indeed, analysis of post-mortem COVID-19 lung suggests two distinct stages of disease-progression. Early disease has high viral-load and high expression of cytokines and ISGs and sparse immune infiltrates, while in late disease, low viral loads, low local expression of cytokines and ISGs, and strong infiltration of macrophages and lymphocytes prevail. Patients who die early are unable to control SARS-CoV-2, while patients who die later suffer from diffuse tissue-damage and immunopathology (Nienhold et al., 2020) suggesting that late disease stage pathogenesis is apparently decoupled from acute viral-load.

### 3.4 Multiple pathologies of COVID-19 phenotypes may converge mechanistically

**FIGURE 8:**
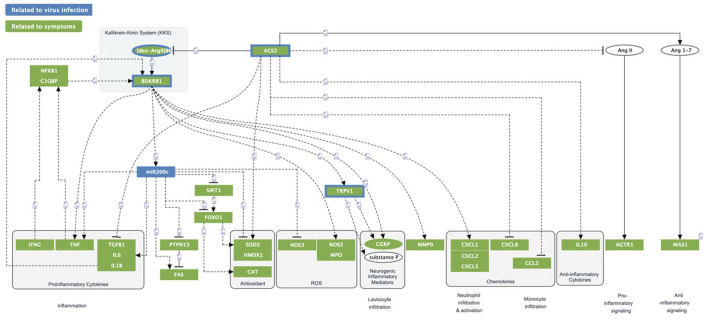
ACE2/DAK/B1R triggered inflammatory processes. Multiple inflammatory processes are triggered by the dysregulation of the ACE2/DAK/B1R axis. Excess activation of B1R triggers Cytokines/Chemokines, ROS inflammation and neurogenic inflammation.

**FIGURE 9:**
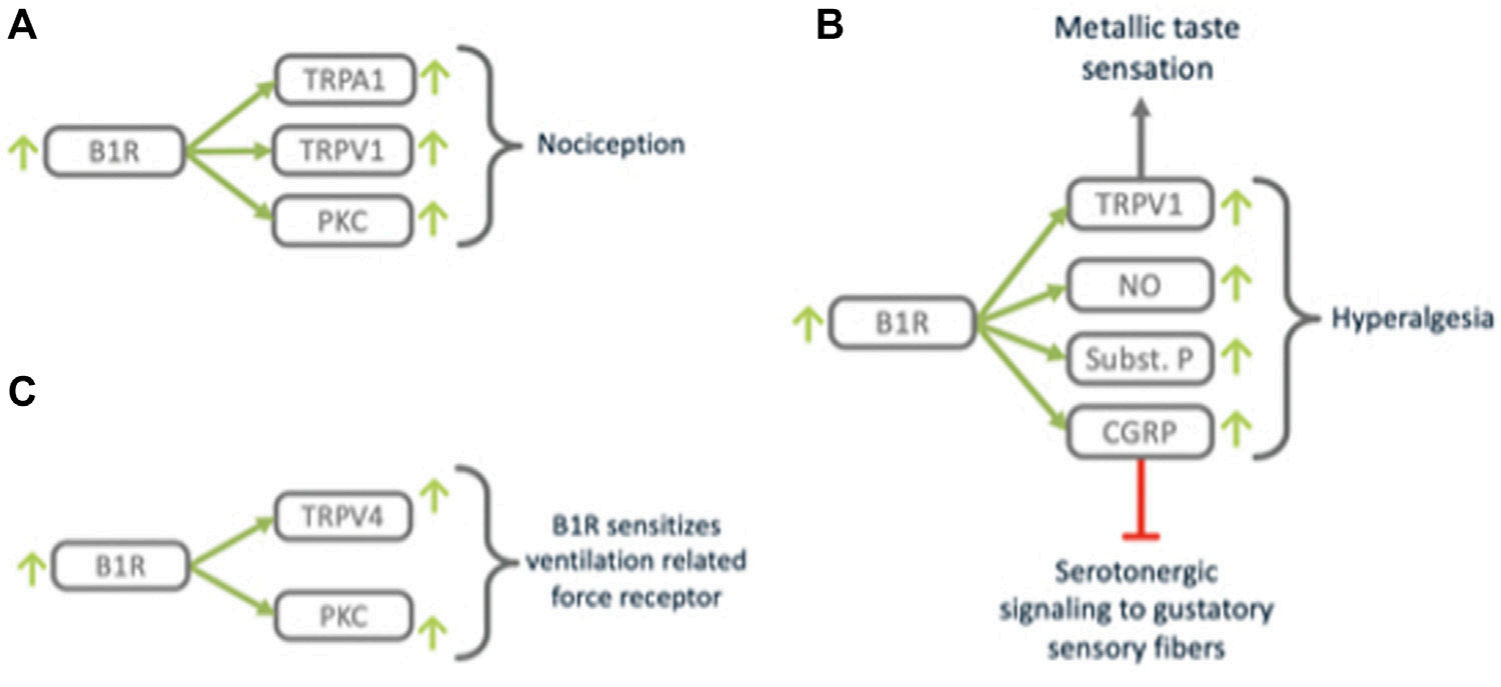
B1R triggers nociception and closely related processes. B1R **(A)** mediates nociception *via* TRPV1, **(B)** modulates taste sensation *via* TRPV1 and neurogenic signaling mediators SubstanceP and CGRP and **(C)** sensitizes mechano-sensing *via* TRPV4.

**FIGURE 10:**
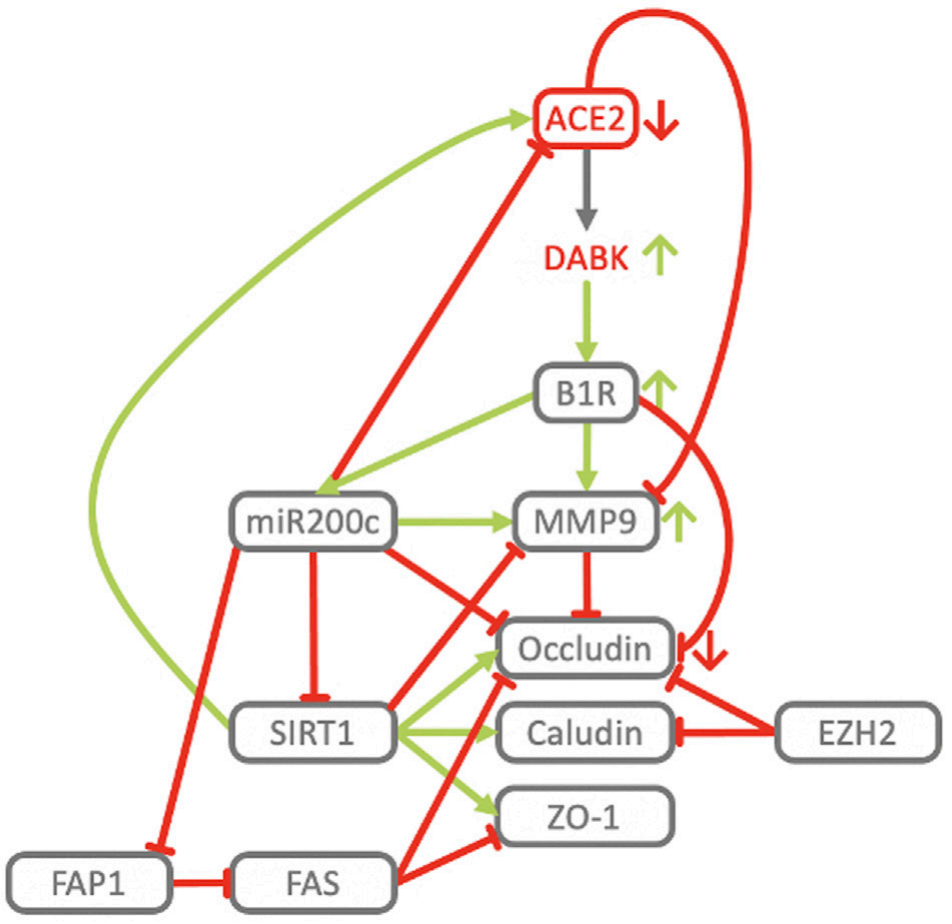
Key players of the ACE2/DAK/B1R axis cooperate in the downregulation of tight junction proteins and induction of MMP9. B1R stimulation results in the loss of Occludin expression at tight junctions and an increase of vascular permeability (Mugisho et al., 2019). In neuroinflammatory diseases B1R contributes to inflammation and loss of blood-brain-barrier integrity, while inhibition of B1R protects mice from focal brain injury by reducing blood-brain barrier leakage and inflammation (Raslan et al., 2010). Neutrophils engage the KKS to open up the endothelial barrier in acute inflammation (Kenne et al., 2019). Activation of B1R induces expression and secretion of MMP-9 and MMP-2 (Matus et al., 2016). Active MMP2 and MMP9 degrade components of the alveolar basement membrane (Dunsmore and Rannels, 1996; Vu, 2001), non-matrix components such as integrins (Greenlee et al., 2007; Vaisar et al., 2009), and intercellular targets such as E-cadherin (Symowicz et al., 2007; Li et al., 2010). MMP-9 levels are elevated in ALI/ARDS (Davey et al., 2011) and predictive of the development of ARDS (Hsu et al., 2015). A distinct increase in circulating MMP-9 has been identified in COVID-19 patients with respiratory failure (Ueland et al., 2020). MMP-9 also exacerbates injury pathways in ischemic stroke, impairs and actively degrades components of the BBB, leading to the development of cerebral edema and hemorrhagic transformation (Turner and Sharp, 2016; Brilha et al., 2017). In CKD, MMP-9 activity is associated with resistant albuminuria (Pulido-Olmo et al., 2016). B1R blockade has been shown to dramatically reduce edema formation not only in ARDS but also in models of acute ischemic stroke (Austinat et al., 2009), traumatic brain injury (Raslan et al., 2010) and multiple sclerosis (Göbel et al., 2011). In accordance with B1R downregulating Occludin and inducing MMP-9, ACE2 deficiency has been associated with increased MMP-9 levels in myocardial infarction (Kassiri et al., 2009), while antago-miR200c, potentially *via* derepression of ACE2, inhibits MMP-9, increases Occludin mRNA and protein expression resulting in increased TJ permeability (Al-Sadi et al., 2017). In contrast, ectopic delivery of miR200c transcriptionally and translationally represses Occludin (Elhelw et al., 2017). The effect of other regulatory elements and effectors of our COVID-19 model, like SIRT1, EZH2 and FAS on barrier integrity is consistent with their respective mechanistic roles in regulating the ACE2-DAK-B1R axis. TMPRSS2 activity may also contribute to increased barrier permeability. TMPRSS2 cleaves and thereby activates PAR2 (Wilson et al., 2005). In airways PAR2 activation induces constriction, increases lung vascular and epithelial permeability and pulmonary edema (Su et al., 2005). In concordance with its general protective role, activation of SIRT1 by Resveratrol maintains the epithelial barrier by increasing the expression of TJ proteins ZO1, Occludin and Claudin1 (Ma et al., 2014), while it negatively regulates MMP9 in diabetic retinopathy, and reduction of SIRT1 levels through oxidative stress confers an increase in MMP9 (Kowluru et al., 2014). Activation of FAS increases barrier permeability and decreases the expression of Occludin and ZO1 in the alveolar-capillary membrane *in vivo* and in human alveolar epithelium *in vitro* (Herrero et al., 2019). FAS is an effector of miR200c and among the most highly induced genes in response to miR200c overexpression (Tryndyak et al., 2010). At the same time miR200c represses FAP1, a negative regulator of FAS (Schickel et al., 2010). Consistent with this, Hepatitis C Virus induced miR200c down modulates FAP1 and promotes fibrosis (Ramachandran et al., 2013). EZH2-knockdown leads to upregulation of Occludin and Claudins (de Vries et al., 2015). EHZ2 expression increases with age, while aging exacerbates ALI-induced changes of the epithelial barrier, lung function, and inflammation. ALI in old mice showed 6x BALF protein, 2x neutrophils, higher levels of CXCL1, ICAM1, MMP-9 and significantly reduced Occludin (Kling et al., 2017).

**FIGURE 11:**
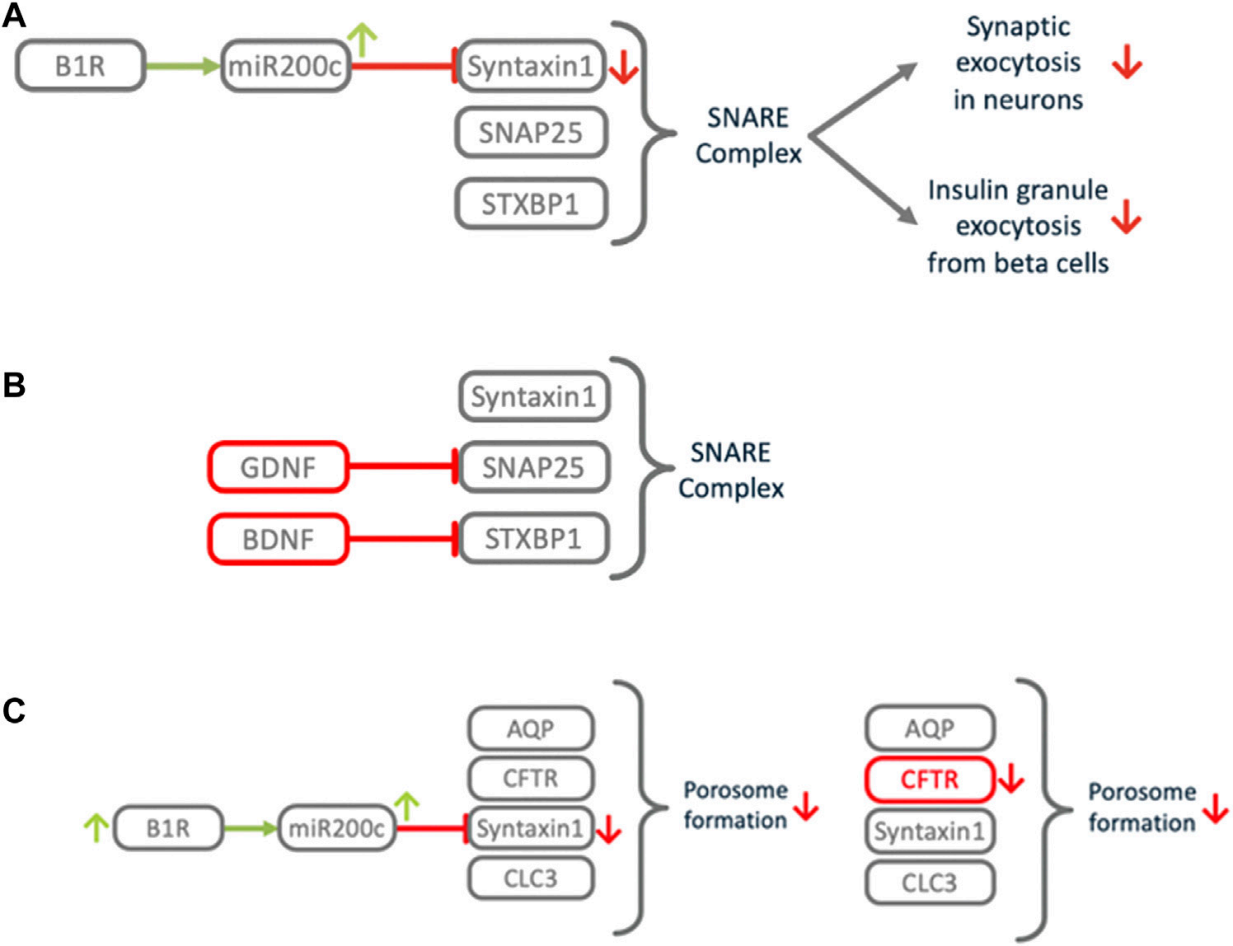
Impact of B1R induced miR200c on exocytosis and related processes. **(A)** Downregulation of Syntaxin1 impairs the SNARE complex, a central element of the exocytosis machinery. This impacts signal transduction between neurons, which might cause COVID-19 associated anosmia, ageusia, cognitive impairment or silent hypoxia. **(B)** Congenital central hypoventilation syndrome (CCHS) causes the same symptoms as COVID-associated silent hypoxemia. In CCHS defects in growth factors GDNF or BDNF cause downmodulation of SNAP25 or STXBP1, leading to SNARE dysfunction in the oxygen sensing carotid bodies. **(C)** COVID-19 associated thick mucus resembles the cystic fibrosis (CF) phenotype. In CF, mutations in CFTR cause porosome dysfunction, which impairs the SNARE dependent transient fusion of secretory vesicles at the porosome. Excess activation of B1R may induce local downregulation of Syntaxin1, which is also involved in this process, thus mimicking the CF phenotype.

**FIGURE 12:**
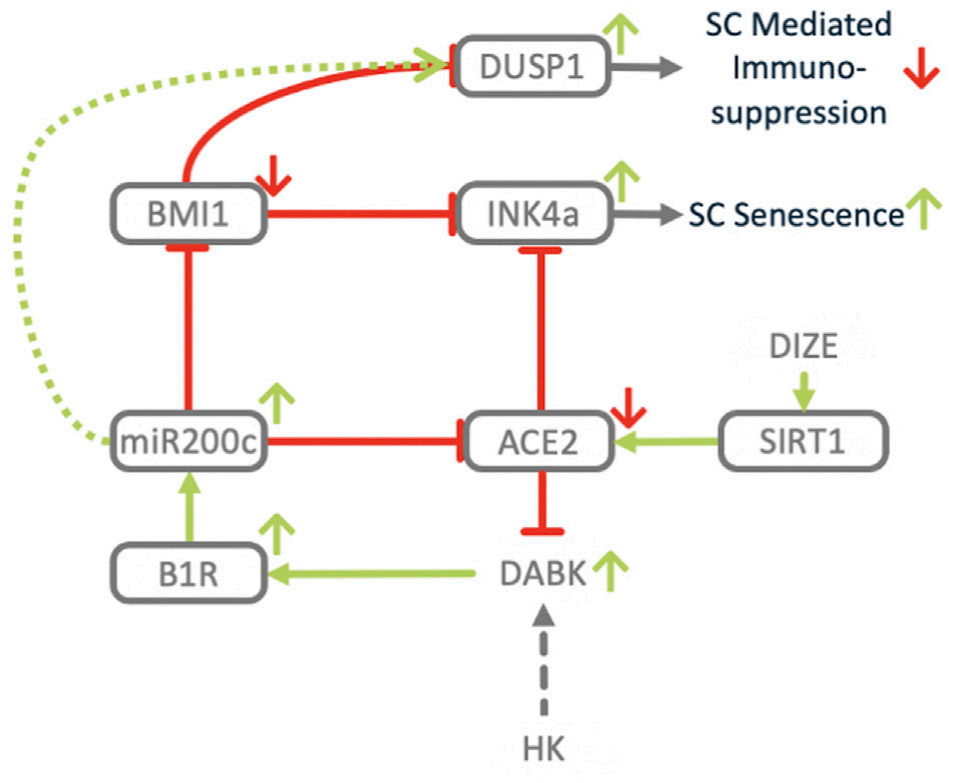
Dysregulation of ACE2/DAK/B1R axis induces senescence. Downregulation of ACE2 and induction of miR200c impact the expression of key regulators of senescence. BMI1. Inhibition of BMI1 *via* upregulation of p16INK4a induces stem cell senescence. Upregulation of DUSP1 has been associated with immune-senescence.

A hyper-inflammatory response is a major cause of severe disease and mortality (Del Valle et al., 2020). Our model demonstrates how excess activation of inflammatory signaling (Figure 8) turns productive inflammatory response and recruitment of immune cells, into a detrimental cytokine storm and immunopathology. Here, coagulation is no longer restricted to the site of infection/injury but leads to disseminated thrombotic events. Likewise, nociception (Figure 9) becomes chronic pain and increased barrier permeability (Figure 10) causes edema. Under normal inflammatory conditions, exocytosis is downregulated to disturb virus dissemination, however, when inflammation remains unresolved, the same mechanism can lead to impairment of neuronal signal transmission (Figure 11). Inflammation is also known to trigger senescence as a pathogen defense mechanism, which under hyper-inflammatory conditions may impair stem cell function and cause autoimmunity (Figure 12). Finally, inflammation triggers fibrogenesis for healing processes which under hyper-inflammation leads to scar formation. Importantly, these mechanisms can be triggered by an imbalance in ACE2-DAK-B1R signaling and associated regulatory components (e.g., miR200c or SIRT1) (Figure 1C).

Altogether, the model suggests that dysregulated homeostasis of eight mechanisms, alone or in combination, may contribute to the pathogenesis of major COVID-19 phenotypes. The multiple and seemingly unrelated clinical faces of COVID-19, including common disease symptoms (e.g., dry cough, myalgia, anosmia, transient diabetes and silent hypoxia), and severe manifestations (e.g., ARDS, lung fibrosis, acute coronary syndromes and thromboembolic events) may largely be linked to the pleiotropic activity of a few core molecular players and mechanisms involved in the host response (Figure 1). Interestingly, our model reveals that some more rare phenotypes can be matched to other diseases sharing the same symptoms. Silent hypoxemia, for instance, causes the same symptoms as the Congenital Central Hereditary Hypoventilation Syndrome (CCHS). The molecular pathologies of both converge on the same molecular mechanism (Figure 11). A full analytical overview of the generated model is summarized in Supplementary Table S1 and made available *via* our COVID-19 Explorer.

### 3.5 Detailed examples of mechanistic convergence

#### 3.5.1 Exocytosis

A number of different manifestations and symptoms of COVID-19 share a common underlying theme. Anosmia, ageusia, silent hypoxemia and neurological dysfunctions possibly result from impairment of signal transmission in the nervous system. As described above, there is a mechanism by which miR200c selectively represses Syntaxin1. Syntaxin1 is an important functional protein for neurotransmission and its dysfunction causes loss-of-function of neural cells (Davis et al., 1998; Watanabe et al., 2013). B1R activation induces secretion of microvesicles (MVs) carrying miR200c from microglial cells which are delivered to neural cells, where miR200c represses Syntaxin1 and damages these cells (Tang et al., 2017). Such a mechanism combines numerous mechanistic principles induced *via* the ACE2/DAK/B1R axis. Syntaxin1 is essential for the exocytosis of synaptic vesicles. Together with SNAP-25 and STXBP1, Syntaxin1 is the starting point for the assembly of the SNARE complex that drives vesicle fusion (Dawidowski and Cafiso, 2016). Mutations in these genes are associated with intellectual disabilities and seizures. Morphogenic analysis of SARS-CoV-2 in human airway epithelial cultures shows that virus release from cells occurs through exocytosis (Zhu et al., 2020). It is therefore possible that under normal circumstances, the B1R mediated downregulation of the synaptic machinery is part of a host defense mechanism, while excess activation of B1R leads to collateral damage. However, it is not known whether Syntaxin1 or other members of the Syntaxin family are responsible for vesicle fusion in this context.

#### 3.5.2 Neurological dysfunction

COVID-19 patients can develop a range of neurological complications. A population study identified long-term cognitive deficits in patients who have recovered from COVID-19 (Hampshire et al., 2020). The scale of the observed deficits equates to an 8.5-point difference in IQ, which is equivalent to the average 10-year decline. One of the key factors contributing to age-related cognitive decline is the dysregulation of the fronto-temporal SNARE protein interactome (Ramos-Miguel et al., 2018). Global cognitive decline is associated with reduced SNARE complex levels (Syntaxin1, SNAP25, VAMP). Synapse dysfunction is initiated early in this process and occurs independent of neuropathology-driven synapse loss (Honer et al., 2012; Boyle et al., 2013), with the SNARE complex and its interactors representing a fingerprint of synaptic functionality. Reduced expression of Syntaxin1 because of B1R overactivation could provide an explanation for the cognitive decline in COVID-19 patients. The proposed virus independent mechanism could also explain the long-term impairment past acute symptoms.

#### 3.5.3 Anosmia, ageusia

ACE2 is expressed in epithelial cells of oral mucosa and is highly enriched in epithelial cells of tongue (Xu et al., 2020), and in fungiform and circumvallate papillae (Shigemura et al., 2019). Olfactory sensory neurons express neither ACE2 nor TMPRSS2, however, epithelial support cells and stem cells express both genes, as do cells in the nasal respiratory epithelium (Sungnak et al., 2020). Non-neural expression of ACE2 and TMPRSS2 in the olfactory epithelium sustentacular cells occurs at levels comparable to that observed in lung cells (Brann et al., 2020). Thus, infection of these cell types, rather than sensory neurons may be responsible for anosmia in COVID-19 patients. Although sensory neurons are likely not targets of infection, intercellular communication *via* MVs could induce defects in taste and olfactory signal processing through the mechanism by which MV carrying miR200c are transferred from infected cells to sensory cells upon excess DAK and B1R activation. This mechanism could explain the observed complete anosmia and ageusia which is beyond mere taste modulation induced through mechanisms associated with nociception (see above). The synaptic release machinery of olfactory sensory neurons is centered on the Syntaxin1-dependent SNARE complex (Marcucci et al., 2009). Also taste cell synapses use the classical Syntaxin1 SNARE machinery for neurotransmitter release in circumvallate taste buds (Yang et al., 2007). Interestingly, Syntaxin1 and the SNARE complex are also potential targets in general anesthesia (van Swinderen and Kottler, 2014) and in the context of Huntington’s disease it was demonstrated that miR200c contributes to neuronal dysfunction by targeting genes regulating synaptic function (Jin et al., 2012).

#### 3.5.4 Silent hypoxemia

One of the distinctive features of COVID-19 is severe hypoxemia often associated with near normal respiratory system compliance, a condition that has been termed silent hypoxemia (Gattinoni et al., 2020; Ottestad et al., 2020; Wilkerson et al., 2020; Xie et al., 2020). This distinct symptom shares commonalities with the rare hereditary disease, congenital central CCHS (Figure 11). CCHS is a life-threatening disorder with impaired ventilatory response to hypoxia and hypercapnia which is related to carotid body (CB) dysfunction. CCHS patients show a significant decrease in the number of dopaminergic vesicles in oxygen sensor cells of CBs (López-Barneo et al., 2008). The oxygen sensor cells are strategically located at the bifurcation of the carotid artery, which supplies the brain. Upon arterial hypoxia they transmit signals to the respiratory center, which increases the frequency of breathing. Dopamine is considered as the predominant transmitter of the CB sensor cells and sensor cells utilize the exocytotic apparatus and its components SNAP25 and Syntaxin1 for signaling (Koerner et al., 2004). BDNF as well as GDNF carry CCHS disease causing mutations (Sasaki et al., 2003). BDNF enhances synaptic functions and restores the severe synaptic dysfunction induced by STXBP1 deficiency. Mutations that disrupt STXBP1 binding to the SNARE severely impair vesicular transmitter release (Lee et al., 2019). GDNF induces expression of SNAP25 (Barrenschee et al., 2015). Defects in GDNF or BDNF lead to SNARE deficiency which impairs exocytosis in CB sensory cells and leads to decreased dopaminergic signaling. In COVID-19 patients B1R/miR200c mediated downregulation of Syntaxin1 in CB sensory cells, which are openly exposed to circulating virus particles, might induce similar impairment of the exocytotic machinery causing silent hypoxemia.

#### 3.5.5 New-onset diabetes

New-onset Diabetes and severe metabolic complications of preexisting Diabetes, including diabetic ketoacidosis and hyperosmolarity, have been observed in patients with COVID-19 (Li et al., 2020a; Chee et al., 2020; Ren et al., 2020). A similar phenotype characterized by virus induced Diabetes was observed with SARS-CoV infections (Yang et al., 2010). In one controlled study (Yang et al., 2010), 39 patients who received no steroid treatment during the disease course and had no concomitant diseases such as pre-existing Diabetes, chronic hepatic, kidney, lung, cardiovascular disorders, cerebrovascular disorders, or blood dyscrasias before infection, were followed-up for 3 years. Fourteen of these 39 patients had Diabetes within 3 days of hospitalization, twenty after 2 weeks. Six patients had Diabetes at discharge and two patients still had Diabetes after 3 years of follow-up. In this study hyperglycemia was a predictor for death.

Pancreatic islet β-cells release Insulin *via* exocytosis of Insulin secretory granules. This process is mediated by distinct membrane fusion machineries (Gaisano, 2012). The fundamental components of this machinery are the three SNARE proteins Syntaxin1, SNAP-SNAP25 and VAMP (Südhof and Rothman, 2009). Syntaxin1 therefore plays a key role in Insulin granule exocytosis and replenishment. In Type-2 Diabetes (T2D), severely reduced islet Syntaxin1 levels contribute to Insulin secretory deficiency (Liang et al., 2017). In COVID-19 patients, ACE2 deficiency and B1R/miR200c mediated downregulation of SyntaxinA1 in β-cells may therefore lead to the induction of new-onset Diabetes. In many instances this phenotype persists even after the virus has been cleared, suggesting a mechanism that can propagate and maintain itself in the absence of virus. ACE2 deficiency has been directly linked to defects in Insulin secretion (Niu et al., 2008) and interestingly, B1R as well as CPN1, the enzyme that converts BK into the B1R ligand DABK have been researched as preclinical targets in the context of Diabetes (El Akoum et al., 2017; Haddad and Couture, 2017). ACE2 deficiency has been directly linked to defects in Insulin secretion (Niu et al., 2008) and interestingly, B1R as well as CPN1, the enzyme that converts BK into the B1R ligand DABK, have been researched as preclinical targets in the context of Diabetes (El Akoum et al., 2017; Haddad and Couture, 2017). B1R antagonism and pharmacological blockade of CPN1 exerted similar beneficial effects in a rat model of Diabetes (Haddad and Couture, 2017). In mouse models, B1R antagonism has been shown to reverse hyperglycemia (Catanzaro et al., 2010). The anti-diabetic effect of B1R antagonism is consistent with the reduction of glycemia in B1R knockout mice (Seguin et al., 2008). Recently it has been found that B1R-expressing adipose tissue coordinates the metabolic response to diet-induced obesity in a cell-nonautonomous manner and furthers adipose tissue remodeling and the development of metabolic syndrome (Sales et al., 2019). miR200c has also been linked to the molecular pathology of Diabetes. It has been shown that miR200c diminishes Insulin production by inducing pancreatic β-cell damage (Belgardt et al., 2015), while suppression of miR200c improves β-cell function in patients with T2D (Roshanravan et al., 2018). miR200c has also been identified as a mediator of diabetic endothelial dysfunction, Diabetes-associated vascular complications (Zhang et al., 2016) and Diabetes-associated cardiac hypertrophy (Singh et al., 2017). Inhibition of miR200c has been shown to restore endothelial function in diabetic mice (Zhang et al., 2016).

#### 3.5.6 Thick mucus

The mucous secretions found in the airways of COVID-19 patients (Mao L. et al., 2020) are reminiscent of those seen in cystic fibrosis (CF) patients (Martínez-Alemán et al., 2017). CF patients have elevated levels of highly viscous mucus in their lungs resulting from mutations that disrupt the CFTR gene (Cutting, 2015). A key property of mucus is its appropriate viscosity that enables its movement by the underlying cilia. Secretion of more viscous mucus hampers its proper transport, resulting in chronic and fatal airway disease. CFTR is known to interact with Syntaxin1, chloride channel CLC-3, and aquaporins to form the porosome complex. The porosome is essential for mucus hydration and controlling viscosity (Jena, 2014). The process of secretion *via* the porosome is similar to exocytosis: a pore is formed through transient fusion of a secretory vesicle at the porosome base *via* SNARE proteins resulting in the formation of a fusion pore. In COVID-19 patients B1R/miR200c mediated downregulation of Syntaxin1 in lung epithelium, a key component for secretion *via* the porosome, could mimic the molecular pathology of CF.

#### 3.5.7 Barrier permeability

One of the most common causes of hospital admission and death in patients with COVID-19 is ARDS, a clinical syndrome characterized by acute lung inflammation and increased-permeability pulmonary edema (Ware, 2020). Severe lung failures such as alveolar edema, ARDS and ALI are caused by increased permeability of the alveolar/airway epithelium and exudate formation. This barrier is sealed by TJs between cells, which are composed of Occludin, ZO1 and various claudins. A loss of TJ permselectivity in the airways results in an uncontrolled leakage of high molecular weight proteins and water into the airways, which finally results in the formation of alveolar edema and ARDS (Wittekindt, 2017). Inflammatory lung diseases like asthma, COPD and allergic airway inflammation may predispose patients to severe lung failures. In COPD, for example, the expression of Occludin and ZO1 in alveolar epithelia cells is reduced (Hong, 2012). The same is true for ventilation induced ALI where expression of Occludin is significantly decreased and alveolar permeability is increased. Upregulation of Occludin can reduce ventilation-induced lung injury (Liu et al., 2014). In sepsis-induced ARDS/ALI downregulation of TJ-proteins coincides with upregulation of inflammatory cytokines. Unfractionated heparin attenuates ALI by upregulating Claudin, ZO1 and Occludin (Liu J. et al., 2019). Down-regulation of TJ proteins is also observed in other pathologies involving epithelial barrier defects such as in chronic kidney disease, which is characterized by a marked depletion of TJ proteins (Claudin1, Occludin, and ZO1) in the gastric epithelium (Vaziri et al., 2013) or Kawasaki disease (KWD) where decreased ZO1 levels are associated with intestinal barrier dysfunction (Lai et al., 2020).

Epithelial TJs regulate alveolar air-fluid balance in the lungs, the production of appropriately concentrated urine in the kidney, as well as the absorption of nutrients and containment of bacteria throughout the gastrointestinal tract (Anderson and Van Itallie, 2009). TJs in endothelia maintain intravascular volume and regulate the flux of fluid and solutes between blood vessels and organ parenchyma (Rahimi, 2017). Therefore, endothelial and epithelial barrier dysfunction can result in malabsorption of nutrients, translocation of pathogens, capillary leak, interstitial edema, tissue dysoxia, and ultimately organ failure.

B1R stimulation results in the loss of Occludin expression at TJs and an increase of vascular permeability (Mugisho et al., 2019). In neuroinflammatory diseases B1R contributes to inflammation and loss of Blood: Brain barrier (BBB) integrity. Inhibition of B1R protects mice from focal brain injury by reducing BBB leakage and inflammation (Raslan et al., 2010). Neutrophils engage the KKS to open up the endothelial barrier in acute inflammation (Kenne et al., 2019). MMP9 also plays an important role in BBB break-down through its ability to degrade the base membrane. Levels of MMP9 are elevated in ALI/ARDS (Davey et al., 2011) and MMP9 activity is predictive of the development of ARDS (Hsu et al., 2015). A distinct increase in circulating MMP9 has been identified in COVID-19 patients with respiratory failure (Ueland et al., 2020). MMP9 also exacerbates injury pathways in ischemic stroke, impairs and actively degrades components of the BBB, leading to the development of cerebral edema and hemorrhagic transformation (Turner and Sharp, 2016; Brilha et al., 2017). In chronic kidney disease, MMP9 activity is associated with resistant albuminuria (Pulido-Olmo et al., 2016). Activation of B1R induces expression and secretion of MMP9 and MMP2 (Matus et al., 2016). Active MMP2 and MMP9 degrade components of the alveolar basement membrane (Dunsmore and Rannels, 1996; Vu, 2001), non-matrix components such as integrins (Greenlee et al., 2007; Vaisar et al., 2009), and intercellular targets such as E-cadherin (Symowicz et al., 2007; Li et al., 2010).

B1R blockade has been shown to dramatically reduce inflammatory processes and edema formation not only in ARDS but also in models of acute ischemic stroke (Austinat et al., 2009), traumatic brain injury (Raslan et al., 2010) and multiple sclerosis (Göbel et al., 2011). Consistent with B1R downregulating Occludin and inducing MMP9, ACE2 deficiency has been associated with increased MMP9 levels in MI (Kassiri et al., 2009), while antago-miR200c, potentially *via* derepression of ACE2, inhibits MMP9, increases Occludin mRNA and protein expression resulting in increased TJ permeability (Al-Sadi et al., 2017). In contrast, ectopic delivery of miR200c transcriptionally and translationally represses Occludin (Elhelw et al., 2017). The effect of other regulatory elements and effectors (Figure 2), like SIRT1, EZH2 and FAS on barrier integrity is consistent with their respective mechanistic roles in regulating the ACE2-DAK-B1R axis. TMPRSS2 activity may also contribute to increased barrier permeability. TMPRSS2 cleaves and thereby activates PAR2 (Wilson et al., 2005).

In airways, PAR2 activation induces constriction, increases lung vascular and epithelial permeability and pulmonary edema, triggers SubstanceP release and increases CXCL2 production (Su et al., 2005). Interestingly, SIRT1 mediates a protective effect on barrier integrity, which is in concordance with its regulatory effect on the ACE2/DAK/B1R signaling axis. Activation of SIRT1 by Resveratrol maintains the epithelial barrier by increasing the expression of TJ proteins ZO1, Occludin and Claudin1 (Ma et al., 2014), while it negatively regulates MMP9 in diabetic retinopathy, and reduction of SIRT1 levels through oxidative stress confers an increase in MMP9 (Kowluru et al., 2014). Activation of FAS increases barrier permeability and decreases the expression of Occludin and ZO1 in the alveolar-capillary membrane *in vivo* and in human alveolar epithelium *in vitro* (Herrero et al., 2019). FAS is an effector of miR200c and among the most highly induced genes in response to miR200c overexpression (Tryndyak et al., 2010). At the same time miR200c represses FAP1, a negative regulator of FAS (Schickel et al., 2010). Consistent with this, Hepatitis C Virus induced miR200c down-modulates FAP1 and promotes fibrosis (Ramachandran et al., 2013). Furthermore, and also consistent with its regulatory role, EZH2-knockdown is accompanied by upregulation of Occludin and Claudins (de Vries et al., 2015). An important factor influencing barrier integrity is age. Aging exacerbates ALI-induced changes of the epithelial barrier, lung function, and inflammation. ALI in old mice showed 6x BALF protein, 2x neutrophils, and higher CXCL1, ICAM1, MMP9 and significantly reduced Occludin levels (Kling et al., 2017).

### 3.6 Complex COVID-19 phenotypes

Complex COVID-19 phenotypes may involve a combination of dysregulated mechanisms. While many symptoms are directly related to individual disease mechanisms triggered by B1R overactivation, more complex organ-level syndromes may involve combinations. For instance, in COVID-19 lungs, hyper-inflammatory phenotypes coincide with increased epithelial permeability and resulting edema formation (Carsana et al., 2020), neutrophilia (Fu et al., 2020), micro-thrombotic events (Merrill et al., 2020), endotheliitis (Varga et al., 2020), fibrosis (Grillo et al., 2020) and impaired self-renewal capacity (Shao et al., 2020). Similarly, cardiovascular (Nishiga et al., 2020), kidney (Pei et al., 2020) and neurologic (Bridwell et al., 2020) manifestations arise from a combination of the SARS-CoV triggered pathogenic events. Vasculitis and KWD as well as GBS serve as examples to describe the concerted interplay of the different pathogenic mechanisms triggered in the context of our COVID-19 disease model.

#### 3.6.1 Vasculitis/Kawasaki disease (KWD)

An unusually high incidence of KWD has been reported for children suffering from COVID-19 (Belhadjer et al., 2020; Jones et al., 2020; Moreira, 2020; Nathan et al., 2020; Riphagen et al., 2020; Rivera-Figueroa et al., 2020; Toubiana et al., 2020; Verdoni et al., 2020). The appearance of clinical manifestations resembling KWD has been attributed to a new phenotype of autoimmunity (Rodríguez et al., 2020). Post-mortem examination of COVID-19 patients reveals damage in many organ systems suggesting a general vascular dysfunction (Menter et al., 2020). Cases of COVID-19 associated cutaneous vasculitis have also been reported (Dominguez-Santas et al., 2020; Papa et al., 2020). Loss of TJs and barrier integrity may also play a role in vasculitis and KWD. The TJ protein ZO1 is decreased in KWD and has been associated with intestinal barrier dysfunction of KWD (Lai et al., 2020). Reduced mRNA expression of multiple intestinal TJs was observed in a murine model of KWD (Rivas et al., 2017) and experimental data as well as clinical, genetic, and transcriptome evidence from patients converge to suggest a key role of IL1β in KWD (Noval Rivas et al., 2019), which is a key upstream regulator of B1R.

Transfer of B1R bearing MVs was identified as novel inflammatory mechanism in vasculitis (Kahn et al., 2017), which is also involved in endothelium-neutrophil communication (Tharaux and Dhaun, 2017). Patients with acute vasculitis show high levels of circulating B1R positive endothelial MVs. B1R positive MVs induce a neutrophil chemotactic effect, which can be blocked by B1 receptor antagonist. Patient plasma induces the release of more B1R positive MVs from endothelial cells, an effect that is dependent on the presence of B1R positive MVs in patient plasma. SERPING1 depleted plasma promoted excessive release of B1R positive endothelial MVs. Addition of SERPING1 or B1R-antagonist inhibited this effect (Mossberg et al., 2017). Serum level of miR200c one of the key regulators in our model has been identified as a suitable diagnostic biomarker and potential target in KWD (Zhang R. et al., 2017).

It has also been demonstrated that plasma from Vasculitis patients has significantly more C3- and C9-positive endothelial MVs than controls. Perfusion of patient acute-phase plasma samples over glomerular endothelial cells induced the release of significantly more complement-positive MVs, in comparison to remission or control plasma. Complement bearing MVs are strongly reduced by B1R antagonism or SERPING1. Likewise, perfusion of glomerular endothelial cells with SERPING1-depleted plasma induced the release of complement-positive MVs, which in turn was significantly reduced by B1R antagonism or SERPING1 (Lopatko Fagerström et al., 2019). Vasculitis often occurs in the context of connective tissue disease. In patients with connective tissue disease, autoantibodies to ACE2 are associated with vasculopathy (Takahashi et al., 2010) and severity of interstitial lung disease is positively correlated with PBMC miR200c, as has been demonstrated for patients with Sjogren’s disease (Jiang et al., 2017). Antineutrophilic autoantibodies (ANCAs), which play a role in endogenous systemic vasculitis (Silva de Souza, 2015) have been identified in cases of COVID-19 associated vasculitis and glomerulonephritis (Uppal et al., 2020) and an autoimmune component has been ascribed to the COVID-19-associated KWD phenotype (Rodríguez et al., 2020). Indeed, anti-endothelial cell autoantibodies have been identified in KWD patients (Sakurai, 2019). Thus, the ACE2/DAK/B1R axis associated mechanism driving autoimmunity and stem cell senescence may play a role in the COVID-19 associated pathogenesis of Vasculitis/KWD.

In summary, Vasculitis and KWD are associated with activation of the complement system, KKS signaling, inflammation, loss of TJs and an autoimmune component. All of these are triggered by the same mechanism underlying our disease model. The disease mechanism of KWD converges with the COVID-19 model at the molecular level, as exemplified by miR200c. This micro-RNA is both a disease specific diagnostic biomarker for KWD, and a key element of the disease model contributing to increased vascular permeability and autoimmunity. Intercellular communication mediated by MVs plays an equally central role in both models.

#### 3.6.2 Guillain-Barré (GBS)

An increasing number of case reports suggest that COVID-19 may induce GBS (Camdessanche et al., 2020; Zhao et al., 2020). GBS is an acute inflammatory demyelinating polyradiculoneuropathy resulting from an autoimmune attack on the myelin. Interestingly, B1R activation contributes to demyelination in multiple sclerosis, while B1R inhibition or its genetic deletion decrease the neuroinflammatory response and myelin loss in the spinal cord (Dutra et al., 2011) and the upstream kininogen was established as a key mediator of neurodegeneration (Langhauser et al., 2012). Serum antibodies against glycolipids, mainly gangliosides, are detected in about 60% of patients with GBS and its variants and play a crucial role in the pathogenic mechanisms of GBS (Uchibori and Chiba, 2015). Overactivation of the ACE2/DAK/B1R axis may mechanistically contribute to the development of GBS and drive the autoimmune reaction.

### 3.7 Risk factors

The model reveals that many of the identified risk-factors comprising obesity, age, sex, smoking and diverse comorbidities such as diabetes or Alzheimer`s disease (Holman et al., 2020; Zhou et al., 2020) are directly related to overlapping pathogenic mechanisms.

#### 3.7.1 Type-2 diabetes (T2D)

It appears that T2D is both a risk factor for severity and in the form of new-onset diabetes, a consequence of COVID-19 (Table 1). Subjects who develop T2D have a complex phenotype with defects in insulin secretion and insulin resistance in target tissues. Key players of our COVID models are associated with both mechanisms. In T2D the ACE2: ACE ratio negatively correlates with HbA1C and loss of ACE2 exacerbates cardiovascular complications of T2D. A potential therapeutic role of ACE2 in the context of T2D has mostly been discussed in the light of its role in the RAS (Batlle et al., 2010). On the other hand, in T2D, SERPING1 is downregulated, while KNG1 is upregulated (Zhang et al., 2013), highlighting a role for the KKS system. This provides additional context to reduced ACE2 activity and its effect on KKS-driven accumulation of DAKs and overactivation of B1R.

**TABLE 1:** Readouts of COVID-19 trials utilizing drugs that have the potential to increase ACE2 expression in the context host response (Chee et al., 2020).

System/Drug group	Drug	Target	Indication (inclusion criteria)	Outcome	References (PMID)
Kinin-Kallikrein	Berinert (C1 inhibitor)	C1R and C1S	Severe COVID-19 pneumonia: SpO2 ≤ 94% in ambient air or Pa02/FiO2 ≤ 300 mmHg	- No change in “time to clinical improvement"	3669276 Yamakawa et al. (1987)
				- No change to coagulation parameters	
				- Eosinophils increased	
	Icatibant (B2R antagonist)	BDKRB2	Severe COVID-19 pneumonia: SpO2 ≤ 94% in ambient air or Pa02/FiO2 ≤ 300 mmHg	- No change in “time to clinical improvement"	3669276 Yamakawa et al. (1987)
				- No change to coagulation parameters	
				- Eosinophils count increased	
	Icatibant (B2R antagonist)	BDKRB2	Severe COVID-19 pneumonia: >3 L/min supplemental oxygen, a CT severity score of ≥7	- 89% (8/9 patients) had reduction of >3 l/min oxygen supplementation 24 h post treatment	32789513 van de Veerdonk et al. (2020)
				- No severe adverse events	
				- No clear association with D-dimer levels or fever	
	Conestat Alfa (C1 inhibitor)	C1R and C1S	Moderate or severe COVID-19 pneumonia by CT scan, C-reactive protein level of at least 30 mg/L oxygen saturation of <93% at rest in ambient air	- Immediate defervescence in 4 out of 5 patients	32922409 Urwyler et al. (2020)
				- 2.5x reduction in intubation or death	
				- No change in length of hospitalization	
ACE Inhibitors and ARBs	Various ACEi and ARBs	ACE and ATR1	COVID-19 patients with ACEi or ARBs prescription before contracting COVID-19. The study compared outcomes between patients randomly assigned to discontinue or continue ACEi or ARBs upon hospitalization	- No difference in outcomes between discontinued or continued use of ACEi or ARBs during COVID-19 hospitalization	33422263 Cohen et al. (2021), 33464336 Lopes et al. (2021)
TRP channels	GSK2798745 (antagonist)	TRPV4	Lung congestion in patients with heart failure: Patients with Heart failure NYHA Class II/III	- Improved lung diffusing capacity for carbon monoxide DLco (only trend not significant)	32227554 Stewart et al. (2020), 30637626 Goyal et al. (2019)
				- No serious adverse events	
Vitamin D	Calcifediol		Patients hospitalized with COVID-19 acute respiratory infection (Radiography confirmed)	- Significant reduction of ICU admission (2% vs. 50%)	32871238 Entrenas Castillo et al. (2020)
				- Limitation—comparison groups not fully matched	
				- No information on BMI (and thus potential obesity)	
Calcium release-activated Calcium (CRAC) channel	Auxora (CM4620) (inhibitor)	ORAI1	Severe COVID-19 pneumonia (chest imaging) with respiratory compromise (e.g. ≥ 30 breaths/min, heart rate ≥125 bpm, SpO_2_ < 93% on room air or PaO_2_/FiO_2_ < 300)	- Reduced median time to recovery (5 vs. 12 days)	32795330 Miller et al. (2020)
				- Reduced risk of intubation (18% vs. 50%)	
				- Combined death or intubation hazard ratio: 0.23	
Non-steroidal anti-inflammatory (NSAIDs)	Acetylsalicylic acid		Patients hospitalized with COVID-19 pneumonia	- Significantly reduced in-hospital death rate	33476420 Meizlish et al. (2021)
	Acetylsalicylic acid + Anti-coagulation (tirofiban, clopidogrel, fondaparinux)		Patients with COIVD19 severe respiratory failure requiring Continuous positive airway pressure (CPAP)	- Reduction in A-a O_2_ gradient	32450344 Viecca et al. (2020)
				- Increased PaO2/FiO2 ratio	
				- Earlier weaning from CPAP	
Glucose lowering drugs	Metformin	PRKAB1, GPD1, ETFDH	Study of susceptibility to contract COVID-19 in diabetic patients using Metformin	- No significant association of metformin and risk of COVID-19	33560344 Wang et al. (2021)
	Metformin	PRKAB1, GPD1, ETFDH	Hospitalized COVID-19 patients using Metformin prior to COVID-19 infection	- Reduced mortality/risk of death	33745895 Cheng et al. (2021), 33662839 Ghany et al. (2021), 33580540 Bramante et al. (2021), 33519709 Crouse et al. (2021), 33309936 Lalau et al. (2021), 33232684 Lally et al. (2020), 33471718 Li et al. (2020b), Meta-analyses: 32844132 Hariyanto and Kurniawan (2020), 33395778 Lukito et al. (2020)
				- Reduced risk of acute ischemic stroke	
				- Some studies point to higher positive effect in females	
				- Some studies show initially more severe course of disease in diabetic patients but overall lower mortality	
	Gliptin (DPP4 inhibitor)	DPP4	Meta-analysis of 9 studies with COVID-19 patients using DPP-4 inhibitor	- Reduced risk ration (RR) of death (0.76)	33838614 Rakhmat et al. (2021)

According to our model, ACE2 deficiency and B1R/miR200c may mediate the downregulation of Syntaxin1 in β-cells, which could impair exocytosis in pancreatic β-cells and hence hamper insulin secretion. Recently, it has been shown that upregulated circulating miR-200c in plasma may increase the risk of severe COVID-19 for obese individuals (Papannarao et al., 2021). On the other hand, miR200c is a biomarker of insulin resistance in obesity. miR200c diminishes insulin production by inducing pancreatic β-cell damage, while its suppression improves β-cell function in T2D and restores endothelial function. miR200c downregulates IRS1, which is associated with insulin resistance. It also targets PGC1A, which controls the hepatic ratio of IRS1 and IRS2 and low PGC1A is associated with insulin resistance. PGC1A is also negatively regulated by TRPV4, while TRPV4 antagonists reduce high‐fat diet‐induced obesity, insulin resistance, diabetic nephropathy, retinopathy, and neuropathy. TRPV4 in turn is sensitized by B1R and its activity is mediated *via* a B1R-PKCε axis. B1R sensitizes PKCε and induces its translocation. PKCε activity has been demonstrated to contribute to lipid-induced insulin resistance. Furthermore, B1R in adipose tissue controls the response to diet-induced obesity and its deletion protects from obesity and improves insulin sensitivity.

In summary, combined evidence from the COVID-19 model connects key players both directly and synergistically to defective insulin secretion and insulin resistance in target organs, providing a rationale for both diabetes as risk factor and a consequence of COVID-19.

#### 3.7.2 Co-morbidities and novel onset diseases

Low levels of ACE2 are associated with more severe outcomes and fatality in severe lung diseases. ACE2 plays a role in asthma, COPD, pulmonary fibrosis, PH, ALI and ARDS amongst others. We also examined the role of ACE2 in disease contexts other than lung. In general, ACE2 plays a protective role and in many of these and it is often downregulated in disease affected tissue. This, for example, is the case in T2D, where a negative correlation between ACE2/ACE vs. HbA1C is observed and loss of ACE2 exacerbates cardiovascular complications in Diabetes (Joshi et al., 2019). ACE2 also plays a role in atherosclerosis, heart failure, cardiac fibrosis, ventricular remodeling, arrhythmia, cerebral ischemia, chronic kidney disease, diabetic nephropathy, liver diseases. Genetic variants of ACE2 are associated with cardiovascular risk, hypertension, hypertensive left ventricular hypertrophy, essential hypertension, AF and cardiomyopathy.

A similar collection of diseases and related phenotypes is affected by pathological B1R signaling (see above). Hence, there is a molecular rationale for the observed COVID-19 co-morbidity risk phenotypes. If the virus directly or indirectly (*via* MVs) affects cells of such diseased organ systems, the downregulation of ACE2 and induction of B1R signaling may aggravate pre-existing conditions or even induce the onset of disease in patients at risk. New-onset Diabetes is one such example of a COVID-19 induced disease phenotype. A bi-directional interplay between the pathogenic mechanisms triggered by COVID-19 and those underlying T2D may both define T2D as severity risk-factor and COVID-19 as a trigger for accelerated development of a Diabetes phenotype.

#### 3.7.3 Smoking and air pollution

Cigarette smoking and air pollution have been associated with higher risk of severe COVID-19 outcomes (Comunian et al., 2020). Since cigarette smoking and nicotine reduce the expression of ACE2, this might contribute to the development of cardiovascular and pulmonary diseases (Jia, 2016; Oakes et al., 2018; Yue et al., 2018). Next, exposure to air particulate matters induces B1R and Kallikrein in lung and heart (Aztatzi-Aguilar et al., 2015) and cigarette smoke induced a significant upregulation in B1R expression level (Al Hariri et al., 2016). In rat lung, cigarette smoke leads to an enhanced expression of B1R (5x) and IL1β (30x), while no increase in levels of B2R or TNFα was observed (Lin et al., 2010). Nicotine also enhances EZH2 expression and EZH2 dependent gene silencing (Vaz et al., 2017; Kumari et al., 2018) which may contribute to the development of COPD (Anzalone et al., 2019). As discussed above, EZH2 is a negative regulator of ACE2 and a positive regulator of TMPRSS2. Thus, air pollution and cigarette smoke may affect pathogenic mechanisms that are involved in COVID-19 pathogenesis at multiple levels, which may increase disease severity in a synergistic manner.

#### 3.7.4 Age and gender

Variation in expression of both TMPRSS2 and ACE2 suggests a credible hypothesis for correlation of vulnerability to SARS-CoV infection to age groups (Wang et al., 2020). However, no significant differences in the infection rate of males and females are found with increasing numbers of COVID‐19 patients (Chen J. et al., 2020; Zhang J. J. et al., 2020; Brüssow, 2020; Huang et al., 2020). On the other hand, the link between age and severity of COVID-19 seems well established and the same is true for co-morbidities which are associated with decreased levels of ACE2 which are mostly age-related diseases. Decreased ACE2 levels found in older patients and cardiovascular disease increase the likelihood of severe COVID-19 (AlGhatrif et al., 2010). A negative correlation between ACE2 expression and COVID‐19 fatality could be established at both population and molecular levels (Chen N. et al., 2020). Highest ACE2 expression levels were identified Asian females (>30% higher than other ethnic groups) and an age‐dependent decrease was observed across all ethnic groups. A strong decrease in ACE2 was found T2D patients and data from human and mice revealed reduced ACE2 expression with inflammatory cytokine treatment and upregulation by estrogen and androgen, both of which decrease with age.

Animal models show that ACE2 expression is dramatically reduced with ageing, with significantly higher ACE2 levels in old females compared to males (Xie et al., 2006). In hematopoietic stem cells, the ACE2/ACE ratio is negatively correlated with age in healthy and in diabetic individuals (Joshi et al., 2019). In airway epithelial cells, DNA methylation near the transcription start site of the ACE2 gene associated with biological age (Corley and Ndhlovu, 2020) and differences in methylation patterns are observed between males and females (Fan et al., 2017). ACE2 is located on the X-Chromosome in a region that escapes X-inactivation (Tukiainen et al., 2017). On the other hand, ACE2 expression is controlled by SRY3, a transcription factor encoded by a gene located on the Y chromosome. SRY3 decreases the activity of ACE2 promoter by 0.5-fold (Milsted et al., 2010), while estrogen upregulates ACE2 expression and causes a protective shift in ACE/ACE2 ratio at the mRNA and protein level (Bukowska et al., 2017). This is consistent with the finding that the rate of degradation of DABK is much higher in women compared with men (Cyr et al., 2001).

The age-dependent expression patterns of EZH2 and SIRT1 are consistent with their respective effects on ACE2 expression. While the activity of ACE2 activator SIRT1 decreases (Hwang et al., 2013), the activity of ACE2 repressor EZH2 increases with age (Dozmorov, 2015; Han and Sun, 2020). Energy sensing *via* AMPK and SIRT1 plays a role in ACE2 regulation. The negative correlation of ACE2 to T2D is mirrored by the finding that SIRT1 in negatively correlated with hyperglycemia (Yang et al., 2018). Caloric restriction *via* energy sensing mechanisms results in SIRT1 activation (Waldman et al., 2018). SIRT1 activators have been linked to beneficial effects on COVID-19 severity and mortality. A recent study suggests positive effects of Metformin (Bramante et al., 2020). Also Melatonin has been suggested as potential COVID-19 treatment (Zhang R. et al., 2020) and a clinical trial assessing the efficacy of Melatonin as preventive drug is underway (Ziaei et al., 2020) (NCT04353128). Beneficial effects of SIRT1 activator resveratrol are also being discussed (Filardo et al., 2020). The potential effect of SIRT1 activation is consistent with its role in attenuation of airway inflammation, ARDS, ALI, fibrosis, endothelial barrier dysfunction and edema and maintaining the epithelial barrier by regulating TJs, described above. Metformin, Melatonin and Resveratrol are well tolerated inducers/activators of SIRT1 that could serve as potentially preventive interventions.

#### 3.7.5 Obesity

Numerous components of our COVID-19 disease model are directly associated with obesity. In obesity, factors that contribute to the molecular pathology of COVID-19 are activated, while protective factors are downmodulated. ACE2 reduces cytokine release and inhibits signaling pathways of tissue fibrosis in experimental models of obesity (Rodrigues Prestes et al., 2017) and its deficiency in epicardial adipose tissue worsens inflammation and cardiac dysfunction in response to diet-induced obesity (Patel et al., 2016). The adipocytokine Apelin induces ACE2 expression (Sato et al., 2013) while its expression is decreased in adipose tissue (Wu et al., 2011). The ACE2 activator, SIRT1, shows an inverse correlation with adiposity. SIRT1 levels strongly negatively correlate with the amount of visceral fat (Mariani et al., 2018) B1R in adipocytes regulates glucose tolerance and predisposition to obesity (Mori et al., 2012) and B1R knockout mice have been shown to be resistant to obesity induced by a high-fat diet (Morais et al., 2015). Furthermore, B1R expression in adipocytes controls adiposity and in turn contributes to whole-body Insulin sensitivity (Sales et al., 2019). It has recently been found that B1R-expressing adipose tissue coordinates the metabolic response to diet-induced obesity and furthers adipose tissue remodeling and the development of metabolic syndrome. Thus, B1R antagonism is considered as therapeutic tool for the treatment of obesity and Diabetes (El Akoum et al., 2017). In conclusion, the same systems that are derailed by the virus in our COVID-19 model are disturbed in obesity and contribute to the development and progression of obesity related diseases (Sales et al., 2019). Thus, B1R antagonism is considered as therapeutic tool for the treatment of obesity and Diabetes (El Akoum et al., 2017). In conclusion, the same systems that are derailed by the virus in our COVID-19 model are disturbed in obesity and contribute to the development and progression of obesity related diseases.

#### 3.7.6 Alzheimer’s disease (AD)

APOE4 is the major AD susceptibility gene (Corder et al., 1993). Homozygous APOE4 has also been associated with significant risk for the development of severe COVID-19, as well as death following infection (Kuo et al., 2020). New research has demonstrated that APOE4-associated cognitive decline in AD is associated with breakdown of the BBB, independent of AD pathology (Montagne et al., 2020). APOE4 activates the CypA-MMP9 pathway leading to accelerated BBB breakdown causing neuronal and synaptic dysfunction, while blockade of the CypA-MMP9 pathway restores BBB integrity followed by normalization of neuronal and synaptic functions. This disease mechanism is complementary to the COVID-19 model resulting in barrier dysfunction through downregulation of TJ proteins and induction of MMP9. AD therefore might constitute a risk factor for COVID-19 severity, while COVID-19 from a mechanistic perspective could contribute to AD progression (see above). Interestingly, CypA interacts with the SARS-CoV Nsp1 protein and plays an important role in virus replication (Liu et al., 2020).

#### 3.7.7 GWAS risk factor converging with ACE2, B1R and mir200c signaling

A genome-wide association study (GWAS) involving 1980 patients with COVID-19 and severe disease (defined as respiratory failure) identified a 3p21.31 gene cluster as a genetic susceptibility locus in patients with COVID-19 with respiratory failure and confirmed a potential involvement of the ABO blood-group system. At locus 3p21.31, the association signal spans the genes SLC6A20, LZTFL1, CCR9, FYCO1, CXCR6 and XCR1 (Ellinghaus et al., 2020). Interestingly, the sodium-imino acid transporter 1 (SIT1/SLC6A20) which co-transports with high affinity l-proline, methylaminoisobutyrate, methyl-proline, and hydroxyproline with sodium and chloride, functionally interacts with ACE2 (Singer and Camargo, 2011). Several members of the SLC6 family transporters, neutral amino acid transporters SLC6A19, SLC6A18 and SLC6A20 interact with ACE2 and its structural homolog TMEM27 (Collectrin). TMEM27 shares 47.8% identity with the non-catalytic extracellular, transmembrane and cytosolic domains of ACE2 and is located next to ACE2 on chromosome Xp22. Expression of ACE2 on the apical membrane of enterocytes as well as TMEM27 in kidney is necessary for the expression of these amino acid transporters in the intestine and kidney, respectively. TMEM27 has been shown to bind proteins involved in intracellular and membrane protein trafficking such Snapin and SNAP25 and plays a role in glucose-stimulated Insulin exocytosis in pancreas. Interestingly, ACE2 deficiency is also linked to defects in Insulin secretion (Niu et al., 2008).

Mutations in SLC6A20 have been associated with Hirschsprung’s disease (Xie et al., 2019). The set of genes associated with Hirschsprung include GDNF, which is also linked to the CCHS (Section 3.5.4). Interestingly, CCHS is frequently complicated with neurocristopathies such as Hirschsprung (Sasaki et al., 2003). Interestingly, the molecular pathologies of these diseases (Diabetes, Hirschsprung, CCHS) all converge at the SNARE mediated exocytosis machinery. Mutations in SLC6A19 cause Hartnup disease which is characterized by neutral aminoaciduria. Under certain conditions, patients may also additionally develop symptoms resembling pellagra including photosensitive dermatitis, ataxia, and psychotic behavior, which are secondary to niacin deficiency due to inadequate intestinal absorption of tryptophan, the precursor for niacin synthesis (Hartnup Disease, 1998). Low levels of essential tryptophan may also lead to low levels in Serotonin, Melatonin and Niacin. Similar neuropsychiatric symptoms have been associated with COVID-19 (Ellul et al., 2020). Some COVID-19 patients develop acute Fanconi syndrome including aminoaciduria (Kormann et al., 2020), which resembles the Collectrin KO phenotype, characterized by a Fanconi-like polyuria, which has been linked to its role in SNARE complex formation (Chu and Le, 2014). Significant decreases in Tryptophan levels have been identified with COVID-19 patients (Thomas et al., 2020) and a clinical trial assessing the effect of Niacin on COVID-19 patients is currently underway (Wilkerson et al., 2020).

In summary, the potential COVID-19 severity associated gene, SLC6A20, and other amino acid transporters known to interact with ACE2 functionally converge with ACE2, B1R and mir200c signaling at the SNARE complex. Dysregulation of the SNARE machinery might play a role in different COVID-19 affected organ systems (gastrointestinal system, CNS) and diseases (see new onset Diabetes, silent hypoxemia/hypoventilation).

### 3.8 Diagnostic and therapeutic implications

Given the importance of the KKS as a key dysregulated pathway in COVID-19, its therapeutic modulation provides a potentially tractable mode-of-action to control the production of active kinins and their des-Arg derivatives. In hereditary angioedema, SERPING1 deficiency leads to accumulation of excess BK, which in turn over-activates B1R. Enzyme replacement therapy and kallikrein inhibition are approved, safe and efficacious treatments for Hereditary Angioedema (HAE). Repurposing these drugs to reestablish cellular control of the CAS might be suitable as host directed therapies for COVID-19 patients. The results of enzyme replacement therapy with C1 Inhibitor are inconclusive, with one study reporting a 2.5 fold reduction in intubation and death, while B2R inhibition had no significant impact (Table 1).

Modulation of ACE2 activity is another potential therapeutic strategy for host directed therapy. Diminazene Aceturate directly enhances ACE2 activity (Qi et al., 2013) and PPARγ activator Rosiglitazone increases ACE2 promoter activity (Scroggin et al., 2012). Activation of SIRT1 increases ACE2 expression, and pharmacological activation of SIRT1 has been shown to attenuate ARDS and lung fibrosis. SIRT1 activators have been linked to beneficial effects on COVID-19 severity and mortality. Melatonin stimulates the SIRT1 signaling pathway (Shah et al., 2017) and has been suggested as a potential COVID-19 treatment (Zhang R. et al., 2020) and a clinical trial assessing its efficacy as preventive drug is underway^218^. Beneficial effects of SIRT1 activator resveratrol are also being discussed (Filardo et al., 2020). Metformin has been demonstrated to exert its protective effect in oxidative injuries *via* enhancing SIRT1 and AMPK expression in human endothelial cells (Hung et al., 2016). Interestingly, Metformin treatment was associated with significantly decreased mortality in COVID-19 patients with Diabetes in a retrospective analysis (Luo et al., 2020). This potential effect of SIRT1 activation is consistent with its role in attenuation of airway inflammation, ARDS, ALI, fibrosis, edema and maintaining endothelial and epithelial barriers. Readouts of trials involving ACE2 modulating drugs are summarized in Table 1. Ongoing COVID-19 trials utilizing ACE2 modulators are listed in Supplementary Table S2 and summarized in Figure 3 of Supplementary Material S1.

According to the COVID-19 model B1R could be a preferred therapeutic target. B1R has been researched as a target for the past 10 years, mainly in the context of hyperalgesia and osteoarthritis. However, no clinical results have been published and most of the reported trials are inactive or have been suspended or discontinued (Supplementary Table S3). In patients hospitalized with severe COVID-19, the use of dexamethasone has been recommended as standard-of-care medication. Dexamethasone results in lower 28-day mortality among patients who received invasive mechanical ventilation or oxygen (Horby et al., 2020). Interestingly, the induction of B1R is sensitive to treatment with dexamethasone (Phagoo et al., 2005). In the context of our disease model this activity may explain Dexamethasone’s striking effects.

COVID-19 associated organ damage cannot be wholly explained by the virus’ organ tropism and localized viral load. In COVID-19 associated kidney disease, for example, viral load is low and unevenly distributed (Puelles et al., 2020; Su et al., 2020), making it difficult to explain the extensive kidney damage seen in some patients (Wang et al., 2021). Currently, there are no diagnostic tools associated with such systemic effects. A systemic virus-independent mechanism requires system-wide distribution of a signal that bears the potential to induce a broad spectrum of pathophysiological dysregulation in disparate organs/tissues. A derailed pleiotropic signaling system such as the KKS/B1R signaling axis provides a likely candidate. In this context circulating microvesicles enriched in B1R and mir200c or circulating mir200c itself could serve as biomarker candidates. Indeed, serum, plasma or PBMC levels of miR200c has been identified as a diagnostic biomarker candidate in different COVID-19 related disease contexts, namely Kawasaki Disease (Zhang W. et al., 2017), Pneumonia (Liu et al., 2017), interstitial lung disease (Jiang et al., 2017) COPD (Cao et al., 2014) and fibrosis of multiple tissues (Yang et al., 2012; Ramachandran et al., 2013; Chen et al., 2017). Recently, it has also been shown that upregulated circulating miR-200c in plasma may increase the risk of severe disease in obese individuals (Papannarao et al., 2021).

Our COVID-19 knowledge model contains several drug targets with potential implications for host-directed therapies. These perspectives are summarized in Supplementary Tables S2, S3. Pharmaceutically tractable target structures include the KKS at multiple levels such as Kallikrein inhibitors, SerpinG1 enzyme replacement or B1R inhibitors. Interestingly, the induction of B1R is sensitive to treatment with dexamethasone. Modulation of ACE2 activity presents another potential angle of attack for host directed therapy either by direct activation (e.g., Diminazene Aceturate) or indirectly by induction, e.g., through SIRT-1 activators such as Melatonin, Resveratrol and Metformin or activators of PPARγ (Dambha-Miller et al., 2020). Readouts of trials utilizing drugs that potentially induce ACE2 expression (Dambha-Miller et al., 2020) are summarized in Table 1.

## 4 Discussion

Working in response to the onset of the COVID-19 pandemic, we developed an iterative, expert-driven strategy to compile published and emergent data into a comprehensive COVID-19 knowledge model. By integrating observed clinical phenotypes with core signaling mechanisms, this interactive resource provides a novel patient-level overview of COVID-19 molecular symptomatology that is intended to aid the rapid and systematic elucidation of potential COVID-19 pathogenic mechanisms. In this work, we report on the insights and hypotheses gleaned through COVID-19 Explorer (Brock et al., 2022). We demonstrated how patient-level knowledge modeling of disease symptomatology provides an effective strategy to deconvolute the complexity of the COVID-19 systemic disease, covering perspectives ranging from the host factors involved in SARS-CoV-2 infection, to “a perfect storm” triggered by SARS-COV-2 induced acute hyper-inflammation to “accelerated aging” in subacute/chronic-COVID-19 syndrome *via* virus-independent engagement of pro-senescence pathways. Overall, our model suggests that viral perturbation of eight key mechanisms, alone or in combination, contributes to the pathogenesis of the primary COVID-19 phenotypes.

In terms of host factors responsible for SARS-CoV-2 entry, the COVID-19 knowledge model highlights the importance of ACE2, TMPRSS2, and components of the ISG response that are specifically dysregulated by SARS-CoV host interaction (ACE2, SERPING1). According to the model, these factors may converge on unifying pleiotropic signaling pathways comprising RAS and KKS as part of the CAS. The concurrent downregulation of ACE2 and SERPING1 may reciprocally amplify the deregulation of KKS, which may in turn lead to a cascading over-activation of downstream signaling, especially in acute COVID-19.

Beyond, the host factors responsible for infection, we also examined the mechanisms driving systemic disease. At the time the model was developed in early 2020, new and apparently unrelated clinical phenotypes were concurrently described, which were immediately linked to the core mechanisms identified by our model (e.g., barrier permeability or exocytosis). For instance, first reports of silent hypoxemia emerged in April 2020. The symptoms of silent hypoxemia were mapped against the symptomatology of heritable diseases, identifying CCHS as a phenotypically related disease. CCHS is caused by dysfunction of the exocytosis machinery in oxygen sensing cells, providing a direct link to our model. A similar link between clinical phenotypes and our model was established upon the first reports of endotheliitis (Varga et al., 2020), vasculitis and the role of micro-thrombotic events in severe disease (Table 1). Here too, direct links between the molecular etiology of the observed symptoms and our model could be drawn and strikingly, by mid-April, first cases of new onset KWD-like disease were reported in children with COVID-19, thereby also suggesting the predictive potential of the model. Indeed, for some clinical phenotypes predicted by our model, clinical observations in the on-going pandemic provided real-time validation of many hypotheses (see Supplementary Table S1 for summary).

The model clearly identifies eight key mechanisms that alone or in combination can contribute to the pathogenesis of observed COVID-19 phenotypes, revealing several functionally connected mechanisms across various organ-systems and identified numerous hypotheses for both, viral-dependent and -independent disease mechanisms, and associated pharmacologic targets that may warrant further evaluation. In this context, the model suggests that circulating microvesicles enriched in B1R and mir200c or circulating mir200c itself may serve as potential biomarkers. Interestingly, miR200c identified as a key player in our model, serves as diagnostic biomarker in KWD and MV-based intercellular communication, plays a key role in the molecular etiology of endotheliitis. Hence, and not surprisingly, cardiovascular complications have rapidly emerged as key-threats in COVID-19 (Supplementary Table S1). Next, besides pharmaceutically tractable target structures such as the KKS at multiple levels such as Kallikrein inhibitors, SerpinG1 enzyme replacement or B1R inhibitors, our model identified B1R as a potential therapeutic target.

From a clinical and translational perspective, the identification of discrete molecular players, their interactions, and their involvement in various signaling pathways, is central in the systematic understanding of COVID-19 disease course and outcome. Notably, the model also provides a mechanistic framework for new insights on potential risk-factors triggering COVID-19 as well as so-called “long-COVID” syndrome, occurring with multiple transient but also persistent symptoms, despite prior viral clearance. For instance, the accelerated aging phenomena may largely be explained by activated senescence pathways provided in the model. From a clinical viewpoint, the model underlines the increasing medical evidence that COVID-19 is a systemic disease, which makes it imperative to also treat it as such. Thus, this not only calls for more holistic therapeutic strategies, but also requires the development of comprehensive diagnostic algorithms and disease management guidelines to enable clinicians to tailor individual therapy plans for each patient (i.e., a personalized medicine approach), to accelerate its initiation and allow for rapid adjustments where necessary (e.g., management of COVID-19 induced ARDS).

While providing potential opportunities and hypotheses for drug repurposing, new therapeutic strategies and the immediate identification of potential safety concerns, our model may also enable a more rational development and prioritization of drug development, and clinical trial design. Notably, during the ongoing pandemic, numerous repurposed therapeutic regimens were initiated, but then revised repeatedly, with some ultimately discontinued due to safety issues and inefficiency/futility (e.g., Hydroxychloroquine). Hence, our model may also provide an evidence-based platform to assess repurposed drug candidates for their safety and overall clinical utility in COVID-19.

Numerous large-scale data and AI-initiatives have been launched to provide a testbed for pandemic forecasting (Sheridan, 2020). However, it should be emphasized that the majority provide only partial knowledge as opposed to a comprehensive understanding of COVID-19. Therefore, we believe that holistic clinico-molecular data mining platforms may represent valuable tools to augment 1) the rapid and evidence-driven response to emergent global health emergencies; 2) the in-depth analysis of any complex disease; 3) the development of more personalized disease management guidelines; and d) the identification and development of new drug candidates and biomarkers. From this perspective, such technology platforms may be of critical interest, especially as a framework for global data- and knowledge-sharing. This is particularly relevant in the face of future pandemics and public health emergencies, where multiple stakeholders must rapidly identify and logically structure tomes of real-time information. Patient level disease models may thus prove critical in our future response to such challenges.

Although our COVID-19 model comes with certain limitations (Brock et al., 2022) it does suggest that the overall strategy of aggregating and connecting existing knowledge in a semi-supervised manner, results in a defendable and testable molecular model that may provide an effective means to expedite response to future public health emergencies. This is supported by the fact that several of our original hypotheses have in the meantime been validated through translational research endeavors. Moreover, the work clearly suggests the utility of digital health technologies in evolving a new generation of disease-specific expert-reviews, with broader knowledge coverage and hypothesis generating capacity than current peer-reviewed based approaches.

Beyond the global implications for understanding the molecular basis of COVID-19, our results also suggest that patient-level models may be adapted for evidence-based care. Using knowledge model frameworks such as the MH Corona Explorer, a deeper understanding of any disease can be generated, linking its molecular foundations with its real-world clinical course and outcomes. Applied to COVID-19, the aim is to enable innovative approaches for the diagnosis and therapy of patients and improved patient care. Innovative joint efforts will be crucial to implement systematic studies combining COVID-19 patient data and digital models to identify new drug targets and biomarkers. Findings from such projects may help define predictors for addressing the progression to a severe disease course or for the long-term illness caused by COVID-19. Such collaborations enable knowledge from clinical practice to be combined with the in-depth COVID-19 disease model and be made directly available to doctors for patient care early and record reliable risk assessments of those affected by COVID-19 in order to be able to use them predictively or prognostically. In the future, we hope to be able to preemptively respond to emergent pandemics by networking information on new virus variants and potentially change the course of the disease and its epidemiological consequences. Adaptable treatment strategies that emerge could serve as a key function in care and thus contribute to the management of future pandemics.

## Data Availability

The datasets presented in this study can be found in online repositories. The names of the repository/repositories and accession number(s) can be found in the article/Supplementary Material.
