# Supplementary figures and images for: Whole patient knowledge modeling of COVID-19 symptomatology reveals common molecular mechanisms
Source: Front Mol Med. 2023 Jan 4;2:1035290. doi: 10.3389/fmmed.2022.1035290 (PMC11285600; doi:10.3389/fmmed.2022.1035290)

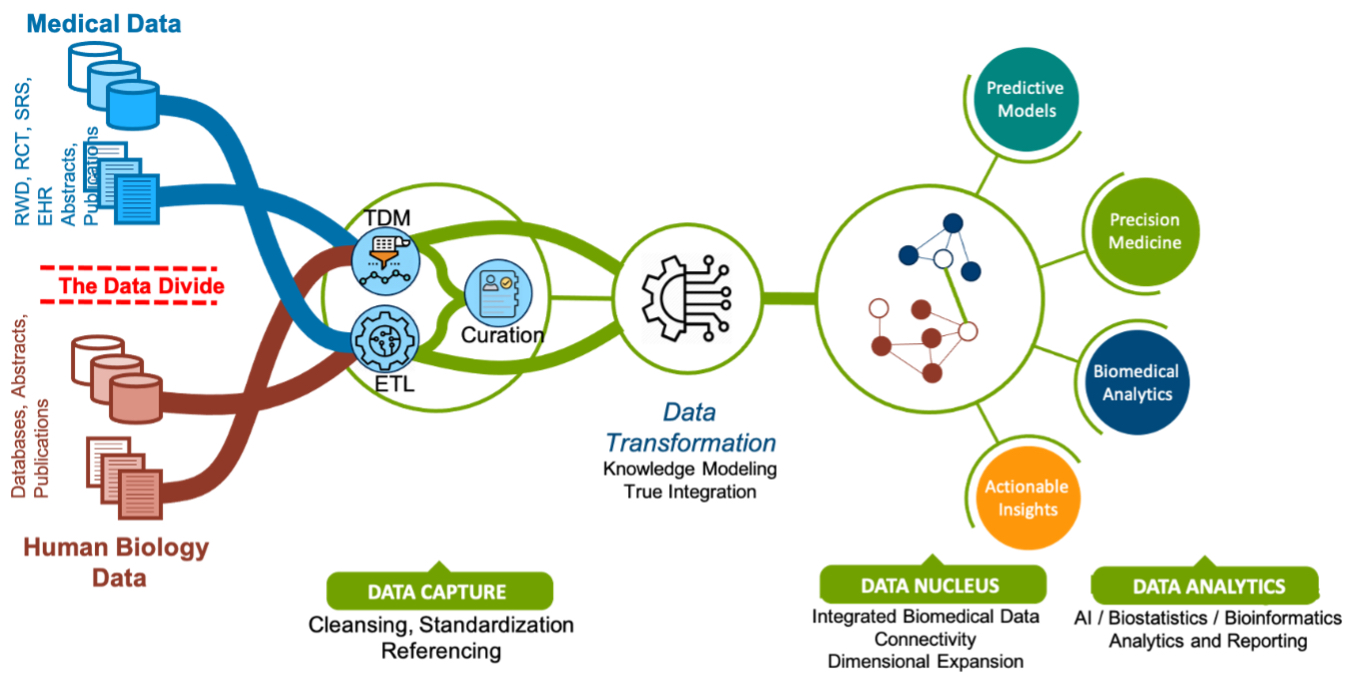

Supplement: Supplementary file 2 [file Image3.JPEG]

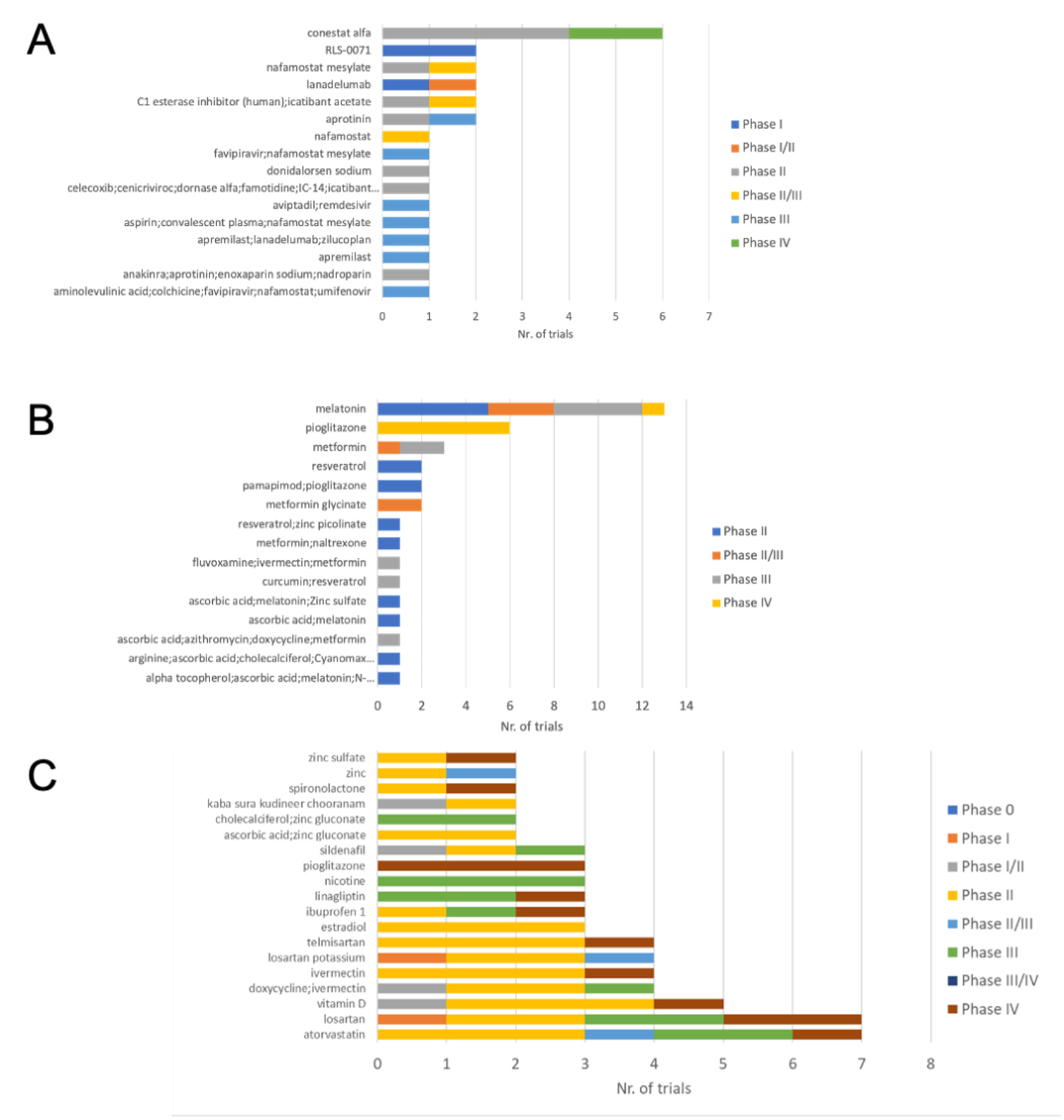

Supplement: Supplementary file 7 [file Image1.JPEG]

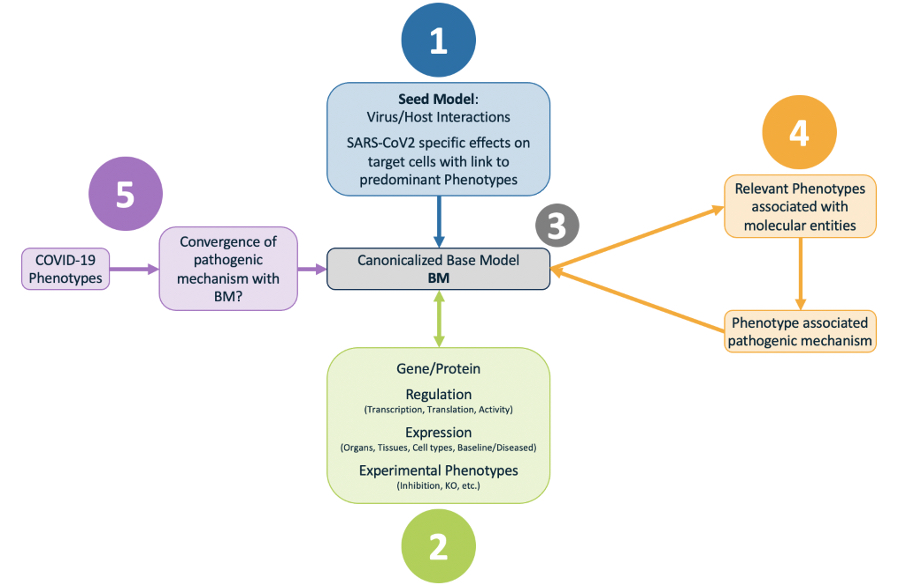

Supplement: Supplementary file 9 [file Image2.JPEG]
