# Supplementary material for: Whole patient knowledge modeling of COVID-19 symptomatology reveals common molecular mechanisms
Source: Front Mol Med. 2023 Jan 4;2:1035290. doi: 10.3389/fmmed.2022.1035290 (PMC11285600; doi:10.3389/fmmed.2022.1035290)
Supplement: Supplementary file 3 [file DataSheet4.DOCX]

**Supplement File 1**

**Whole patient knowledge modeling of COVID-19 symptomatology reveals common molecular mechanisms**

**Stephan Brock ^5^, David B. Jackson ^5^, Theodoros G. Soldatos ^5^, Klaus Hornischer ^5^, Anne Schäfer ^5^, Francesca Diella ^5^, Maximilian Y. Emmert ^1,2,3,4, *^, Simon P. Hoerstrup ^1,2, *^**

*^1^Institute for Regenerative Medicine, University of Zurich, Zurich, Switzerland*

*^2^Wyss Zurich, University of Zurich and ETH Zurich, Zurich, Switzerland*

*^3^Department of Cardiothoracic and Vascular Surgery, German Heart Institute Berlin, Berlin, Germany*

*^4^Department of Cardiovascular Surgery, Charité Universitätsmedizin Berlin, Berlin, Germany*

*^5^Molecular Health GmbH, 69115 Heidelberg, Germany*

*** Correspondence:**  **Corresponding Authors**

[**maximilian.emmert@irem.uzh.ch**](mailto:maximilian.emmert@irem.uzh.ch)  
[**simon.hoerstrup@irem.uzh.ch**](mailto:simon.hoerstrup@irem.uzh.ch)

Address for Correspondence:

Professor Maximilian Y. Emmert, MD, PhD

Institute for Regenerative Medicine (IREM),

Moussonstrasse 13, 8044 Zurich, Switzerland

Tel.: +41 44 634 5610

Fax : +41 44 634 56 08

E-Mail: [maximilian.emmert@irem.uzh.ch](mailto:maximilian.emmert@irem.uzh.ch)

Professor Simon P. Hoerstrup, MD, PhD

Institute for Regenerative Medicine (IREM),

Moussonstrasse 13, 8044 Zurich, Switzerland

Fax : +41 44 634 56 08

Tel.: +41 44 634 5610

E-Mail: [simon.hoerstrup@irem.uzh.ch](mailto:simon.hoerstrup@irem.uzh.ch)

**Keywords: COVID-19, SARS-CoV-2, molecular mechanisms, disease modeling, evidence-based medicine, translational research, knowledge engineering**

Table of Contents

[1 Glossary 3](#_Toc113022675)

[1.1 List of abbreviations 3](#_Toc113022676)

[1.2 HGNC symbols of listed gene names 5](#_Toc113022677)

[2 Materials and Methods 6](#_Toc113022678)

[2.1 Data Availability 6](#_Toc113022679)

[Online Availability 6](#_Toc113022680)

[3 Supplementary Tables 7](#_Toc113022681)

[4 Supplementary Figures 8](#_Toc113022682)

[5 References 11](#_Toc113022683)

# Glossary

## List of abbreviations

ACI: Augmented Clinicomics Intelligence

ACMG: American College of Medical Genetics

AD: Alzheimer’s disease

AF: Atrial fibrillation

AHFS: American Hospital Formulary Service

ALI: Acute lung injury

ANCAs: Antineutrophilic auto-antibodies

AR: Androgen receptor

ARDS: Acute respiratory distress syndrome

ATC: Anatomical Therapeutic Chemical (ATC) classification system

BBB: Blood Brain Barrier

BK: Bradykinin

CAS: Contact activation system

CCHS: Congenital central (hereditary) hypoventilation syndrome

CB: Carotid body

CF: Cystic fibrosis

CNS: Central nervous system

COPD: Chronic Obstructive Pulmonary Disease

COVID-19: Coronavirus disease 2019

DAK: des-Arg-kinins (i.e., DABK and/or DAKD)

DABK: des-Arg^9^-kinins

DAKD: des-Arg^10^-KD

DIC: Disseminated intravascular coagulation / coagulopathy

DIZE: Diminazene aceturate

DPC: Dermal papilla cells

EHR: Electronic health records

EPC: Endothelial progenitor cell

ESMO: European Society for Medical Oncology

ETL: Extraction, Transformation, and Loading

FAERS: FDA’s Adverse Event Reporting System (USA)

FDA: Food and Drug Administration (USA)

GBS: Guillain–Barré syndrome

GPML: GenMAPP Pathway Markup Language

GWAS: Genome-wide association study

HGNC: HUGO Gene Nomenclature Committee

HK: High molecular weight kininogen

HPO: Human Phenotype Ontology

HUGO: Human Genome Organisation

ICD: International Classification of Disease

ICTRP: International Clinical Trials Registry Platform

ISG: Interferon stimulated gene

ISO: International Organization for Standardization

KD: Kallidin

KWD: Kawasaki disease

KEGG: Kyoto Encyclopedia of Genes and Genomes

KKS: Kallikrein Kinin system

LPS: Lipopolysaccharide

MedDRA: Medical Dictionary for Regulatory Activities

MESH: Medical Subject Headings

MH: Molecular Health GmbH

MI: Myocardial infarction

MSC: Mesenchymal stem cell

MV: Microvesicle

NCCN: National Comprehensive Cancer Network

NLM: National Library of Medicine (USA)

NLP: Natural language processing

PAH: Pulmonary arterial hypertension

PH: Pulmonary hypertension

PK or PreKK: Prekallikrein

RAS: Renin-angiotensin system

RCT: Randomized clinical trials

ROS: Reactive Oxygen Species

RWD: Real world data

SARS-CoV: Severe Acute Respiratory Syndrome CoronaVirus

SLE: Systemic lupus erythematosus

SVG: Scalable Vector Graphics

T2D: Type 2 Diabetes

TDM: Text-/Data-Mining

TJ: Tight junction

UCSC: University of California-Santa Cruz

UMLS: Unified Medical Language System

VILI: Ventilator induced (acute) lung injury

WHO: World Health Organization

## HGNC symbols of listed gene names

| **Gene name(s) in publication text** | **HGNC** | |
| --- | --- | --- |
| (main article and supplementary material) | **Approved symbol** | **ID** |
| *AP1 (or c-Jun)* | JUN | 6204 |
| *APP* | XPNPEPL1; XPNPEP2 | 12822; 12823 |
| *AT1R* | AGTR1 | 336 |
| *AT2R* | AGTR2 | 338 |
| *B1R* | BDKRB1 | 1029 |
| *B2R* | BDKRB2 | 1030 |
| *Beclin-1* | BECN1 | 1034 |
| *Claudin1* | CLDN1 | 2032 |
| *CLC3* | CLCN3 | 2021 |
| *CypA* | PPIA | 9253 |
| *eNOS* | NOS3 | 7876 |
| *FAB1* | PIKFYVE | 23785 |
| *FXII* | F12 | 3530 |
| *gC1qR* | C1QBP | 1243 |
| *IFNγ* | IFNG | 5438 |
| *IL1α* | IL1A | 5991 |
| *IL1β* | IL1B | 5992 |
| *IL8* | CXCL8 | 6025 |
| *iNOS* | NOS2 | 7873 |
| *Kininogen (and derivatives BK, DAK)* | KNG1 | 6383 |
| *KK (or preKK or PK)* | KLKB1 | 6371 |
| *KLK2* | KLK2 | 6363 |
| *Mas1R* | MAS1 | 6899 |
| *Mortalin* | HSPA9 | 5244 |
| *NEP* | MME | 7154 |
| *NFκB* | NFKB1 | 7794 |
| *Occludin* | OCLN | 8104 |
| *Oct-1* | **POU2F1** | 9212 |
| *p16INK4a* | CDKN2A | 1787 |
| *PAR2* | F2RL1 | 3538 |
| *PEA3* | ETV4 | 3493 |
| *Pit-1a* | POU1F1 | 9210 |
| *POP, PEP* | PREP | 9358 |
| *Syntaxin1* | STX1A | 11433 |
| *TMEM27* | CLTRN | 29437 |
| *TNFa* | TNF | 11892 |
| *XCR* | XCR1 | 1625 |
| *ZO1* | TJP1 | 11827 |
| *HO1* | HMOX1 | 5013 |

# Materials and Methods

## Data Availability

## Online Availability

As the COVID-19 Explorer and the associated knowledge resource represents one of the main parts of our interactive paper, all data, methods and code/software associated with the atlas of whole patient symptomatology and the molecular pathways associated with these physiological effects can be downloaded within a dedicated download section of the COVID-19 Explorer interface, accessible at:

<https://covid19.molecularhealth.com/basemodel>

<https://covid19.molecularhealth.com/submodels?type=disease>

<https://covid19.molecularhealth.com/submodels?type=functional>

**(Login Information: Username: review, Password: E3cYN4pK).**

Furthermore, other raw, un-curated data pertaining to any aspect of this submission, will also be made available upon reasonable request. In addition, the following supplementary files and folders are provided:

**Supplementary Data File 1**

The supplementary file **``Submodels.csv``** contains summaries of all submodels along with associated symptoms, genes, organs, and the underlying references as Pubmed Unique Identifiers (PMIDs)

**Supplementary Data File 2**

The supplementary Zip Folder **``submodels_svg``** contains SVGs files of all submodels.

**Supplementary Data File 3**

The supplementary Zip Folder **``submodels_csv``** contains CSV files containing all binary interactions of the submodels.

In this context, it is also important to emphasize that the development of the COVID-19 Explorer was enabled by our DATAOME platform. The DATAOME is an expansive biomedical data and analytics infrastructure that has been developed for over a decade by our collaborators at Molecular Health Inc., in Heidelberg, Germany

# Supplementary Tables

Supplementary Table 1: File = 'Supplementary_Table_1.xlsx’ containing contents from the Covid19 Cockpit, summarizing COVID-19 clinical phenotypes, pathogenic mechanisms and the relation to the COVID-19 disease model for each of the SARS-CoV-2 perturbed eight key mechanisms. This file also contains an overview of the validation status for many of the clinical and molecular hypotheses generated during the course of this work (see 4^th^ column). A link to the COVID-19 Explorer is provided at the beginning of each section.

Supplementary Table 2: File = 'Supplementary_Table_2.xlsx’ containing ongoing COVID-19 trials utilizing ACE2 modulating drugs.

Supplementary Table 3: File = ‘Supplementary_Table_3.xlsx’ containing B1R inhibitors and their development status are listed.

# Supplementary Figures


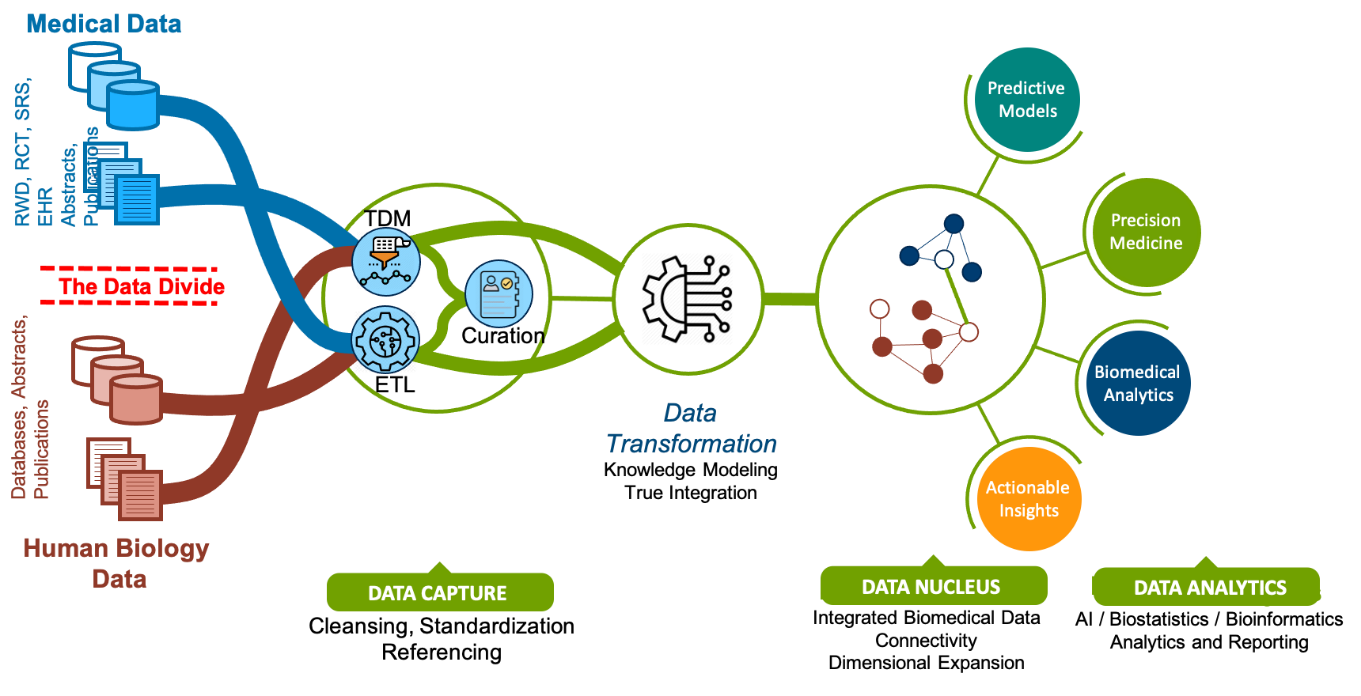


**Supplement Figure 1: Schematic overview of the Dataome technology deployed for COVID-19 disease modeling.**

The Dataome technology provides structured integration of clinical (e.g. Real World Data (RWD), Randomized Control Trials (RCT), Spontaneous Reporting System (SRS) and Electronic Health Record (EHR)) together with molecular data and knowledge. Through the *Data Capture* module, data is integrated (using text data mining (TDM) and automated Extraction, Transformation and Loading (ETL) processes) and normalized before integration into the *Data Nucleus*, using a transformation pipeline. This integrated data then serves as the clinico-molecular knowledgebase upon which decision support technologies and discovery applications are built (*Data Analytics*) providing opportunities regarding next-generation predictive models, precision medicine biomedical analytics and actionable insights.


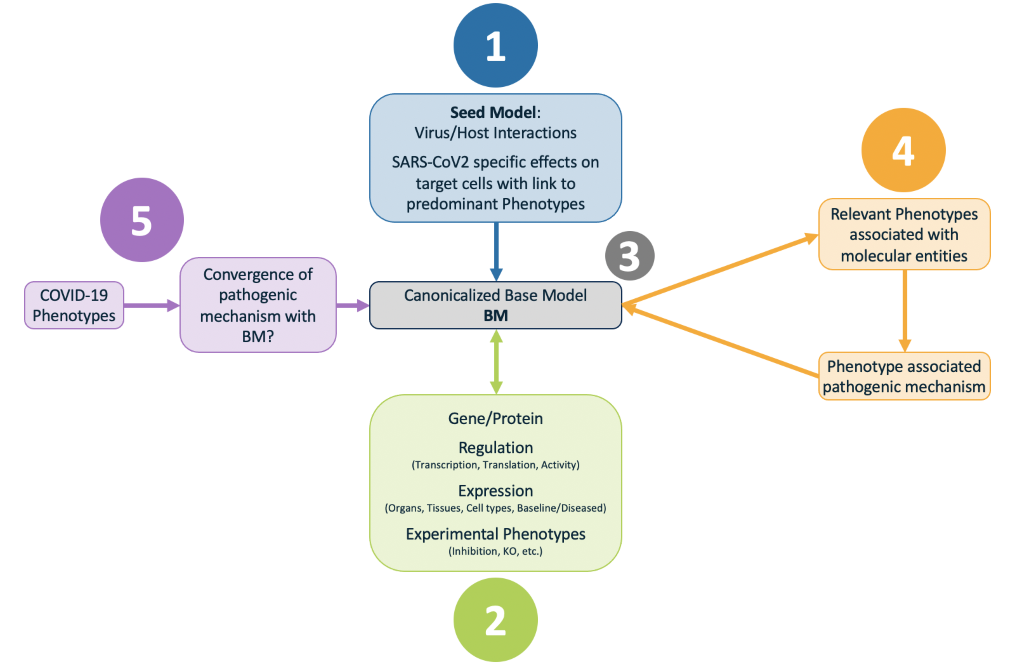


**Supplement Figure 2: Overview of the analytical workflow.**

The DATAOME technology enables the efficient and systematic traversing of large volumes of genotype, chemotype and phenotype information in a standardized manner. Steps 1-3: For generation of the initial seed model, virus-host interactions and effects on the innate immune response were captured. A minimum set of elements defining host cells and the host-specific response were included into the seed model. Regulatory elements, interactors, tissue specific expression and experimental phenotypes were mapped onto the seed model to generate a base model. Steps 4 and 5: Phenotypes associated with members of the base model were collected and screened for relevance w.r.t COVID-19 phenotypes. In parallel, clinical COVID-19 phenotypes were collected. Pathogenic mechanisms underlying these phenotypes were analyzed and screened for convergence with the base model. Results were integrated into the final disease model.


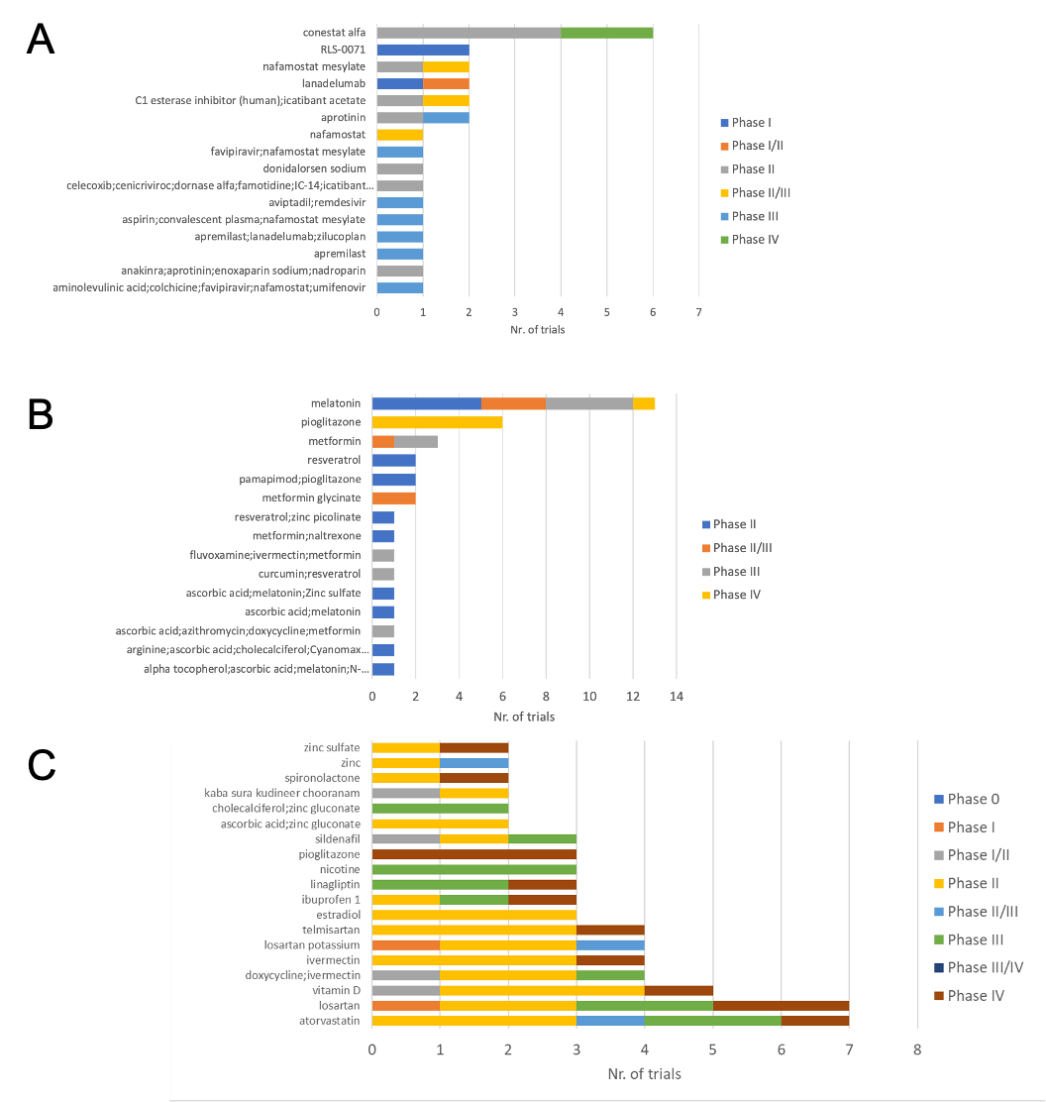


**Supplement Figure 3:** Number of COVID-19 trials utilizing drugs targeting **A)** KKS and C1 inhibitors, **B)** SIRT1, PPARG, and **C)** other potential ACE2 modulating drugs**^304^**.

# References

1. Pruitt, K.D., Tatusova, T. & Maglott, D.R. NCBI Reference Sequence (RefSeq): a curated non-redundant sequence database of genomes, transcripts and proteins. *Nucleic Acids Res* **33**, D501-504 (2005).

2. Kim, S. et al. PubChem 2019 update: improved access to chemical data. *Nucleic Acids Res* **47**, D1102-d1109 (2019).

3. AHFS. <https://www.ahfsdruginformation.com/>.

4. Brown, E.G., Wood, L. & Wood, S. The medical dictionary for regulatory activities (MedDRA). *Drug Saf* **20**, 109-117 (1999).

5. FDA Orange Book. <https://www.accessdata.fda.gov/scripts/cder/ob/index.cfm>.

6. MeSH. <https://www.ncbi.nlm.nih.gov/mesh>.

7. Bodenreider, O. The Unified Medical Language System (UMLS): integrating biomedical terminology. *Nucleic Acids Res* **32**, D267-270 (2004).

8. Köhler, S. et al. Expansion of the Human Phenotype Ontology (HPO) knowledge base and resources. *Nucleic Acids Res* **47**, D1018-d1027 (2019).

9. UniProt: a worldwide hub of protein knowledge. *Nucleic Acids Res* **47**, D506-d515 (2019).

10. Angers, M. et al. In vivo protein-DNA interactions at the kinin B(1) receptor gene promoter: no modification on interleukin-1 beta or lipopolysaccharide induction. *J Cell Biochem* **78**, 278-296 (2000).

11. Yates, A.D. et al. Ensembl 2020. *Nucleic Acids Res* **48**, D682-d688 (2020).

12. Kent, W.J. et al. The human genome browser at UCSC. *Genome Res* **12**, 996-1006 (2002).

13. Landrum, M.J. et al. ClinVar: improvements to accessing data. *Nucleic Acids Res* **48**, D835-d844 (2020).

14. Sherry, S.T. et al. dbSNP: the NCBI database of genetic variation. *Nucleic Acids Res* **29**, 308-311 (2001).

15. Wishart, D.S. et al. DrugBank 5.0: a major update to the DrugBank database for 2018. *Nucleic Acids Res* **46**, D1074-d1082 (2018).

16. Kanehisa, M. & Goto, S. KEGG: kyoto encyclopedia of genes and genomes. *Nucleic Acids Res* **28**, 27-30 (2000).

17. Jassal, B. et al. The reactome pathway knowledgebase. *Nucleic Acids Res* **48**, D498-d503 (2020).

18. Lindquist, M. VigiBase, the WHO Global ICSR Database System: Basic Facts. *Drug Information Journal* **42**, 409 - 419 (2008).

19. FAERS. <https://open.fda.gov/data/faers/>.

20. ESMO. <https://www.esmo.org/>.

21. NCCN. <https://www.nccn.org/>.

22. Flask micro web framework. <https://palletsprojects.com/p/flask/>.

23. Qadri, F. & Bader, M. Kinin B1 receptors as a therapeutic target for inflammation. *Expert Opin Ther Targets* **22**, 31-44 (2018).

24. Duggirala, H.J. et al. Use of data mining at the Food and Drug Administration. *J Am Med Inform Assoc* **23**, 428-434 (2016).

25. Soldatos, T.G., Taglang, G. & Jackson, D.B. In Silico Profiling of Clinical Phenotypes for Human Targets Using Adverse Event Data. *High Throughput* **7** (2018).

26. Schotland, P. et al. Target adverse event profiles for predictive safety in the post-market setting. *Clin Pharmacol Ther* (2020).

27. Gordon, D.E. et al. A SARS-CoV-2 protein interaction map reveals targets for drug repurposing. *Nature* **583**, 459-468 (2020).

28. Kutmon, M. et al. PathVisio 3: an extendable pathway analysis toolbox. *PLoS Comput Biol* **11**, e1004085 (2015).

29. Bostock, M., Ogievetsky, V. & Heer, J. D³: Data-Driven Documents. *IEEE Trans Vis Comput Graph* **17**, 2301-2309 (2011).

30. Jia, H.P. et al. ACE2 receptor expression and severe acute respiratory syndrome coronavirus infection depend on differentiation of human airway epithelia. *J Virol* **79**, 14614-14621 (2005).

31. Xu, H. et al. High expression of ACE2 receptor of 2019-nCoV on the epithelial cells of oral mucosa. *Int J Oral Sci* **12**, 8 (2020).

32. Ziegler, C.G.K. et al. SARS-CoV-2 Receptor ACE2 Is an Interferon-Stimulated Gene in Human Airway Epithelial Cells and Is Detected in Specific Cell Subsets across Tissues. *Cell* **181**, 1016-1035.e1019 (2020).

33. Hamming, I. et al. Tissue distribution of ACE2 protein, the functional receptor for SARS coronavirus. A first step in understanding SARS pathogenesis. *J Pathol* **203**, 631-637 (2004).

34. Wu, Y.H., Li, J.Y., Wang, C., Zhang, L.M. & Qiao, H. The ACE2 G8790A Polymorphism: Involvement in Type 2 Diabetes Mellitus Combined with Cerebral Stroke. *J Clin Lab Anal* **31** (2017).

35. Tao, L. et al. Angiotensin-converting enzyme 2 activator diminazene aceturate prevents lipopolysaccharide-induced inflammation by inhibiting MAPK and NF-κB pathways in human retinal pigment epithelium. *J Neuroinflammation* **13**, 35 (2016).

36. Zhang, X. et al. Angiotensin-converting enzyme 2 regulates autophagy in acute lung injury through AMPK/mTOR signaling. *Arch Biochem Biophys* **672**, 108061 (2019).

37. Xue, T., Wei, N., Xin, Z. & Qingyu, X. Angiotensin-converting enzyme-2 overexpression attenuates inflammation in rat model of chronic obstructive pulmonary disease. *Inhal Toxicol* **26**, 14-22 (2014).

38. Zhang, J. et al. AMP-activated Protein Kinase Phosphorylation of Angiotensin-Converting Enzyme 2 in Endothelium Mitigates Pulmonary Hypertension. *Am J Respir Crit Care Med* **198**, 509-520 (2018).

39. Li, G. et al. Angiotensin-converting enzyme 2 activation protects against pulmonary arterial hypertension through improving early endothelial function and mediating cytokines levels. *Chin Med J (Engl)* **125**, 1381-1388 (2012).

40. Haga, S. et al. A novel ACE2 activator reduces monocrotaline-induced pulmonary hypertension by suppressing the JAK/STAT and TGF-β cascades with restored caveolin-1 expression. *Exp Lung Res* **41**, 21-31 (2015).

41. Jia, H. Pulmonary Angiotensin-Converting Enzyme 2 (ACE2) and Inflammatory Lung Disease. *Shock* **46**, 239-248 (2016).

42. Zou, Z. et al. Angiotensin-converting enzyme 2 protects from lethal avian influenza A H5N1 infections. *Nat Commun* **5**, 3594 (2014).

43. Kuba, K. et al. A crucial role of angiotensin converting enzyme 2 (ACE2) in SARS coronavirus-induced lung injury. *Nat Med* **11**, 875-879 (2005).

44. Imai, Y. et al. Angiotensin-converting enzyme 2 protects from severe acute lung failure. *Nature* **436**, 112-116 (2005).

45. Unthank, J.L. et al. Molecular basis for impaired collateral artery growth in the spontaneously hypertensive rat: insight from microarray analysis. *Physiol Rep* **1**, e0005 (2013).

46. Li, X. et al. Angiotensin converting enzyme-2 is protective but downregulated in human and experimental lung fibrosis. *Am J Physiol Lung Cell Mol Physiol* **295**, L178-185 (2008).

47. Wang, L., Wang, Y., Yang, T., Guo, Y. & Sun, T. Angiotensin-Converting Enzyme 2 Attenuates Bleomycin-Induced Lung Fibrosis in Mice. *Cellular physiology and biochemistry : international journal of experimental cellular physiology, biochemistry, and pharmacology* **36**, 697-711 (2015).

48. El-Hashim, A.Z. et al. Angiotensin-(1-7) inhibits allergic inflammation, via the MAS1 receptor, through suppression of ERK1/2- and NF-κB-dependent pathways. *Br J Pharmacol* **166**, 1964-1976 (2012).

49. Magalhães, G.S. et al. Angiotensin-(1-7) attenuates airway remodelling and hyperresponsiveness in a model of chronic allergic lung inflammation. *Br J Pharmacol* **172**, 2330-2342 (2015).

50. Tan, W.S.D., Liao, W., Zhou, S., Mei, D. & Wong, W.F. Targeting the renin-angiotensin system as novel therapeutic strategy for pulmonary diseases. *Curr Opin Pharmacol* **40**, 9-17 (2018).

51. Serfozo, P. et al. Ang II (Angiotensin II) Conversion to Angiotensin-(1-7) in the Circulation Is POP (Prolyloligopeptidase)-Dependent and ACE2 (Angiotensin-Converting Enzyme 2)-Independent. *Hypertension* **75**, 173-182 (2020).

52. Rodrigues Prestes, T.R., Rocha, N.P., Miranda, A.S., Teixeira, A.L. & Simoes, E.S.A.C. The Anti-Inflammatory Potential of ACE2/Angiotensin-(1-7)/Mas Receptor Axis: Evidence from Basic and Clinical Research. *Curr Drug Targets* **18**, 1301-1313 (2017).

53. Patel, V.B., Zhong, J.C., Grant, M.B. & Oudit, G.Y. Role of the ACE2/Angiotensin 1-7 Axis of the Renin-Angiotensin System in Heart Failure. *Circ Res* **118**, 1313-1326 (2016).

54. Ferreira, A.J. et al. Angiotensin-converting enzyme 2 activation protects against hypertension-induced cardiac fibrosis involving extracellular signal-regulated kinases. *Exp Physiol* **96**, 287-294 (2011).

55. Gaddam, R.R., Chambers, S. & Bhatia, M. ACE and ACE2 in inflammation: a tale of two enzymes. *Inflamm Allergy Drug Targets* **13**, 224-234 (2014).

56. (2020).

57. Patel, V.B. et al. Loss of angiotensin-converting enzyme-2 exacerbates diabetic cardiovascular complications and leads to systolic and vascular dysfunction: a critical role of the angiotensin II/AT1 receptor axis. *Circ Res* **110**, 1322-1335 (2012).

58. Kazemi-Bajestani, S.M., Patel, V.B., Wang, W. & Oudit, G.Y. Targeting the ACE2 and Apelin Pathways Are Novel Therapies for Heart Failure: Opportunities and Challenges. *Cardiol Res Pract* **2012**, 823193 (2012).

59. Sato, T. et al. Apelin is a positive regulator of ACE2 in failing hearts. *J Clin Invest* **123**, 5203-5211 (2013).

60. Wu, H.Z., Z.; Du Q.; Yao, H.; Li, Z.; Wu, L.; Sun, B.; Dai, Q. Down-regulation of Apelin in obesity-related hypertensive rats induced by high-fat diet: Possible role of angiotensin II–AT1R system. *International Journal of Cardiology* **152**, S50-S51 (2011).

61. Palaiodimos, L. et al. Severe obesity, increasing age and male sex are independently associated with worse in-hospital outcomes, and higher in-hospital mortality, in a cohort of patients with COVID-19 in the Bronx, New York. *Metabolism* **108**, 154262 (2020).

62. Huang, S., Chen, L., Lu, L. & Li, L. The apelin-APJ axis: A novel potential therapeutic target for organ fibrosis. *Clin Chim Acta* **456**, 81-88 (2016).

63. Marceau, F., Lussier, A., Regoli, D. & Giroud, J.P. Pharmacology of kinins: their relevance to tissue injury and inflammation. *Gen Pharmacol* **14**, 209-229 (1983).

64. Dray, A. & Perkins, M. Bradykinin and inflammatory pain. *Trends Neurosci* **16**, 99-104 (1993).

65. Couture, R., Harrisson, M., Vianna, R.M. & Cloutier, F. Kinin receptors in pain and inflammation. *Eur J Pharmacol* **429**, 161-176 (2001).

66. Calixto, J.B. et al. Kinin B1 receptors: key G-protein-coupled receptors and their role in inflammatory and painful processes. *Br J Pharmacol* **143**, 803-818 (2004).

67. Leeb-Lundberg, L.M., Marceau, F., Müller-Esterl, W., Pettibone, D.J. & Zuraw, B.L. International union of pharmacology. XLV. Classification of the kinin receptor family: from molecular mechanisms to pathophysiological consequences. *Pharmacol Rev* **57**, 27-77 (2005).

68. Prado, G.N. et al. Mechanisms regulating the expression, self-maintenance, and signaling-function of the bradykinin B2 and B1 receptors. *Journal of cellular physiology* **193**, 275-286 (2002).

69. Marceau, F. Kinin B1 receptors: a review. *Immunopharmacology* **30**, 1-26 (1995).

70. He, R. et al. Activation of AP-1 signal transduction pathway by SARS coronavirus nucleocapsid protein. *Biochem Biophys Res Commun* **311**, 870-876 (2003).

71. McLean, P.G., Perretti, M. & Ahluwalia, A. Kinin B(1) receptors and the cardiovascular system: regulation of expression and function. *Cardiovasc Res* **48**, 194-210 (2000).

72. Ghebrehiwet, B. et al. Soluble gC1qR is an autocrine signal that induces B1R expression on endothelial cells. *J Immunol* **192**, 377-384 (2014).

73. Al-Sadi, R., Rawat, M. & Ma, T. P-272 MMP-9 Modulation of Intestinal Epithelial Tight Junction Permeability: Role of Micro-RNA Regulation of Occludin. *Inflammatory Bowel Diseases* **23**, S88-S88 (2017).

74. Guo, W.X., Ghebrehiwet, B., Weksler, B., Schweitzer, K. & Peerschke, E.I. Up-regulation of endothelial cell binding proteins/receptors for complement component C1q by inflammatory cytokines. *J Lab Clin Med* **133**, 541-550 (1999).

75. Bengtson, S.H., Eddleston, J., Christiansen, S.C. & Zuraw, B.L. Double-stranded RNA increases kinin B1 receptor expression and function in human airway epithelial cells. *Int Immunopharmacol* **7**, 1880-1887 (2007).

76. Knoops, K. et al. SARS-coronavirus replication is supported by a reticulovesicular network of modified endoplasmic reticulum. *PLoS biology* **6**, e226 (2008).

77. Weber, F., Wagner, V., Rasmussen, S.B., Hartmann, R. & Paludan, S.R. Double-stranded RNA is produced by positive-strand RNA viruses and DNA viruses but not in detectable amounts by negative-strand RNA viruses. *J Virol* **80**, 5059-5064 (2006).

78. Walker, K., Perkins, M. & Dray, A. Kinins and kinin receptors in the nervous system. *Neurochem Int* **26**, 1-16; discussion 17-26 (1995).

79. Wu, J. et al. Identification of bradykinin receptors in clinical cancer specimens and murine tumor tissues. *Int J Cancer* **98**, 29-35 (2002).

80. Böckmann, S. & Paegelow, I. Kinins and kinin receptors: importance for the activation of leukocytes. *Journal of leukocyte biology* **68**, 587-592 (2000).

81. Enquist, J., Skröder, C., Whistler, J.L. & Leeb-Lundberg, L.M. Kinins promote B2 receptor endocytosis and delay constitutive B1 receptor endocytosis. *Mol Pharmacol* **71**, 494-507 (2007).

82. Faussner, A., Bathon, J.M. & Proud, D. Comparison of the responses of B1 and B2 kinin receptors to agonist stimulation. *Immunopharmacology* **45**, 13-20 (1999).

83. Marceau, F. et al. Kinin receptors: functional aspects. *Int Immunopharmacol* **2**, 1729-1739 (2002).

84. Lamb, M.E., Zhang, C., Shea, T., Kyle, D.J. & Leeb-Lundberg, L.M. Human B1 and B2 bradykinin receptors and their agonists target caveolae-related lipid rafts to different degrees in HEK293 cells. *Biochemistry* **41**, 14340-14347 (2002).

85. Lu, Y., Liu, D.X. & Tam, J.P. Lipid rafts are involved in SARS-CoV entry into Vero E6 cells. *Biochem Biophys Res Commun* **369**, 344-349 (2008).

86. Wu, Y.H. et al. A novel fine tuning scheme of miR-200c in modulating lung cell redox homeostasis. *Free Radic Res* **51**, 591-603 (2017).

87. Liu, Q. et al. miRNA-200c-3p is crucial in acute respiratory distress syndrome. *Cell Discovery* **3**, 17021 (2017).

88. Jiang, Z. et al. The correlation between miR-200c and the severity of interstitial lung disease associated with different connective tissue diseases. *Scand J Rheumatol* **46**, 122-129 (2017).

89. Cao, Z. et al. microRNA-183 down-regulates the expression of BKCaβ1 protein that is related to the severity of chronic obstructive pulmonary disease. *Hippokratia* **18**, 328-332 (2014).

90. Carlomosti, F. et al. Oxidative Stress-Induced miR-200c Disrupts the Regulatory Loop Among SIRT1, FOXO1, and eNOS. *Antioxid Redox Signal* **27**, 328-344 (2017).

91. Kellner, M. et al. ROS Signaling in the Pathogenesis of Acute Lung Injury (ALI) and Acute Respiratory Distress Syndrome (ARDS). *Adv Exp Med Biol* **967**, 105-137 (2017).

92. Janssen, W.J. & Nozik-Grayck, E. Power of Place: Intravascular Superoxide Dismutase for Prevention of Acute Respiratory Distress Syndrome. *Am J Respir Cell Mol Biol* **56**, 147-149 (2017).

93. Totura, A.L. et al. Toll-Like Receptor 3 Signaling via TRIF Contributes to a Protective Innate Immune Response to Severe Acute Respiratory Syndrome Coronavirus Infection. *mBio* **6**, e00638-00615 (2015).

94. Tryndyak, V.P., Beland, F.A. & Pogribny, I.P. E-cadherin transcriptional down-regulation by epigenetic and microRNA-200 family alterations is related to mesenchymal and drug-resistant phenotypes in human breast cancer cells. *Int J Cancer* **126**, 2575-2583 (2010).

95. Koroleva, E.P., Fu, Y.X. & Tumanov, A.V. Lymphotoxin in physiology of lymphoid tissues - Implication for antiviral defense. *Cytokine* **101**, 39-47 (2018).

96. Li, S.W. et al. SARS coronavirus papain-like protease induces Egr-1-dependent up-regulation of TGF-β1 via ROS/p38 MAPK/STAT3 pathway. *Sci Rep* **6**, 25754 (2016).

97. Bhattacharyya, S., Fang, F., Tourtellotte, W. & Varga, J. Egr-1: new conductor for the tissue repair orchestra directs harmony (regeneration) or cacophony (fibrosis). *J Pathol* **229**, 286-297 (2013).

98. Joyce-Brady, M.F. & Tuder, R.M. Just in the "Bik" of time. *Am J Respir Crit Care Med* **183**, 1447-1448 (2011).

99. Zhang, J. et al. Global transcriptional regulation of STAT3- and MYC-mediated sepsis-induced ARDS. *Ther Adv Respir Dis* **13**, 1753466619879840 (2019).

100. Schickel, R., Park, S.M., Murmann, A.E. & Peter, M.E. miR-200c regulates induction of apoptosis through CD95 by targeting FAP-1. *Molecular cell* **38**, 908-915 (2010).

101. Ramachandran, S. et al. Hepatitis C virus induced miR200c down modulates FAP-1, a negative regulator of Src signaling and promotes hepatic fibrosis. *PLoS One* **8**, e70744 (2013).

102. Magenta, A. et al. The Oxidative Stress-Induced miR-200c Is Upregulated in Psoriasis and Correlates with Disease Severity and Determinants of Cardiovascular Risk. *Oxid Med Cell Longev* **2019**, 8061901 (2019).

103. D'Agostino, M. et al. Circulating miR-200c is up-regulated in paediatric patients with familial hypercholesterolaemia and correlates with miR-33a/b levels: implication of a ZEB1-dependent mechanism. *Clin Sci (Lond)* **131**, 2397-2408 (2017).

104. Tao, J. et al. miR-200c Modulates the Pathogenesis of Radiation-Induced Oral Mucositis. *Oxid Med Cell Longev* **2019**, 2352079 (2019).

105. Aunin, E., Broadley, D., Ahmed, M.I., Mardaryev, A.N. & Botchkareva, N.V. Exploring a Role for Regulatory miRNAs In Wound Healing during Ageing:Involvement of miR-200c in wound repair. *Sci Rep* **7**, 3257 (2017).

106. Cheng, Z. et al. Identification of TMPRSS2 as a Susceptibility Gene for Severe 2009 Pandemic A(H1N1) Influenza and A(H7N9) Influenza. *J Infect Dis* **212**, 1214-1221 (2015).

107. Li, Y., Li, H. & Zhou, L. EZH2-mediated H3K27me3 inhibits ACE2 expression. *Biochem Biophys Res Commun* **526**, 947-952 (2020).

108. Kim, J. et al. Polycomb- and Methylation-Independent Roles of EZH2 as a Transcription Activator. *Cell Rep* **25**, 2808-2820.e2804 (2018).

109. Zhang, Q. et al. Novel pharmacological inhibition of EZH2 attenuates septic shock by altering innate inflammatory responses to sepsis. *Int Immunopharmacol* **76**, 105899 (2019).

110. Yacoub, R., Lee, K. & He, J.C. The Role of SIRT1 in Diabetic Kidney Disease. *Frontiers in endocrinology* **5** (2014).

111. Clarke, N.E., Belyaev, N.D., Lambert, D.W. & Turner, A.J. Epigenetic regulation of angiotensin-converting enzyme 2 (ACE2) by SIRT1 under conditions of cell energy stress. *Clin Sci (Lond)* **126**, 507-516 (2014).

112. Shao, M. et al. Exogenous angiotensin (1-7) directly inhibits epithelial-mesenchymal transformation induced by transforming growth factor-β1 in alveolar epithelial cells. *Biomedicine & pharmacotherapy = Biomedecine & pharmacotherapie* **117**, 109193 (2019).

113. Moran, C.S. et al. Resveratrol Inhibits Growth of Experimental Abdominal Aortic Aneurysm Associated With Upregulation of Angiotensin-Converting Enzyme 2. *Arterioscler Thromb Vasc Biol* **37**, 2195-2203 (2017).

114. Zeng, Z. et al. Activation and overexpression of Sirt1 attenuates lung fibrosis via P300. *Biochem Biophys Res Commun* **486**, 1021-1026 (2017).

115. Dai, Y. et al. Sirtuin 1 is required for antagonist-induced transcriptional repression of androgen-responsive genes by the androgen receptor. *Molecular endocrinology (Baltimore, Md.)* **21**, 1807-1821 (2007).

116. Lu, L. et al. Inhibition of SIRT1 increases EZH2 protein level and enhances the repression of EZH2 on target gene expression. *Chin Med Sci J* **26**, 77-84 (2011).

117. Vachharajani, V. & McCall, C.E. Sirtuins: potential therapeutic targets for regulating acute inflammatory response? *Expert Opin Ther Targets* **24**, 489-497 (2020).

118. Ma, L. et al. 3,5,4'-Tri-O-acetylresveratrol Attenuates Lipopolysaccharide-Induced Acute Respiratory Distress Syndrome via MAPK/SIRT1 Pathway. *Mediators Inflamm* **2015**, 143074 (2015).

119. Jin, W. The role of SIRT1 in LPS-induced lung endothelial barrier dysfunction and lung injury in vivo. *European Respiratory Journal* **48**, PA895 (2016).

120. Fu, C. et al. Activation of SIRT1 ameliorates LPS-induced lung injury in mice via decreasing endothelial tight junction permeability. *Acta Pharmacol Sin* **40**, 630-641 (2019).

121. Zhang, Y.Q. et al. Resveratrol ameliorates lipopolysaccharide-induced epithelial mesenchymal transition and pulmonary fibrosis through suppression of oxidative stress and transforming growth factor-β1 signaling. *Clin Nutr* **34**, 752-760 (2015).

122. Peng, Z., Zhang, W., Qiao, J. & He, B. Melatonin attenuates airway inflammation via SIRT1 dependent inhibition of NLRP3 inflammasome and IL-1β in rats with COPD. *Int Immunopharmacol* **62**, 23-28 (2018).

123. Hung, C.H., Chan, S.H., Chu, P.M., Lin, H.C. & Tsai, K.L. Metformin regulates oxLDL-facilitated endothelial dysfunction by modulation of SIRT1 through repressing LOX-1-modulated oxidative signaling. *Oncotarget* **7**, 10773-10787 (2016).

124. Li, L., Chen, Z., Fu, W., Cai, S. & Zeng, Z. Emerging Evidence concerning the Role of Sirtuins in Sepsis. *Crit Care Res Pract* **2018**, 5489571 (2018).

125. Chu, H. et al. Sirtuin1 Protects against Systemic Sclerosis-related Pulmonary Fibrosis by Decreasing Proinflammatory and Profibrotic Processes. *Am J Respir Cell Mol Biol* **58**, 28-39 (2018).

126. Ma, Y. et al. [Role of SIRT1 in the protection of intestinal epithelial barrier under hypoxia and its mechanism]. *Zhonghua Wei Chang Wai Ke Za Zhi* **17**, 602-606 (2014).

127. Tang, M. et al. Protective action of B1R antagonist against cerebral ischemia-reperfusion injury through suppressing miR-200c expression of Microglia-derived microvesicles. *Neurol Res* **39**, 612-620 (2017).

128. Murça, T.M. et al. Oral administration of an angiotensin-converting enzyme 2 activator ameliorates diabetes-induced cardiac dysfunction. *Regul Pept* **177**, 107-115 (2012).

129. Rangarajan, S. et al. Metformin reverses established lung fibrosis in a bleomycin model. *Nat Med* **24**, 1121-1127 (2018).

130. Watanabe, Y. et al. Point mutation in syntaxin-1A causes abnormal vesicle recycling, behaviors, and short term plasticity. *J Biol Chem* **288**, 34906-34919 (2013).

131. Davis, S. et al. Increase in syntaxin 1B mRNA in hippocampal and cortical circuits during spatial learning reflects a mechanism of trans-synaptic plasticity involved in establishing a memory trace. *Learn Mem* **5**, 375-390 (1998).

132. Dawidowski, D. & Cafiso, D.S. Munc18-1 and the Syntaxin-1 N Terminus Regulate Open-Closed States in a t-SNARE Complex. *Structure* **24**, 392-400 (2016).

133. Zhu, N. et al. Morphogenesis and cytopathic effect of SARS-CoV-2 infection in human airway epithelial cells. *Nat Commun* **11**, 3910 (2020).

134. Hampshire, A. et al. Cognitive deficits in people who have recovered from COVID-19 relative to controls: An N=84,285 online study. *medRxiv*, 2020.2010.2020.20215863 (2020).

135. Ramos-Miguel, A. et al. Frontotemporal dysregulation of the SNARE protein interactome is associated with faster cognitive decline in old age. *Neurobiol Dis* **114**, 31-44 (2018).

136. Honer, W.G. et al. Cognitive reserve, presynaptic proteins and dementia in the elderly. *Transl Psychiatry* **2**, e114 (2012).

137. Boyle, P.A. et al. Much of late life cognitive decline is not due to common neurodegenerative pathologies. *Ann Neurol* **74**, 478-489 (2013).

138. Shigemura, N. et al. Expression of Renin-Angiotensin System Components in the Taste Organ of Mice. *Nutrients* **11** (2019).

139. Sungnak, W., Huang, N., Bécavin, C. & Berg, M. SARS-CoV-2 Entry Genes Are Most Highly Expressed in Nasal Goblet and Ciliated Cells within Human Airways. *ArXiv* (2020).

140. Brann, D.H. et al. Non-neuronal expression of SARS-CoV-2 entry genes in the olfactory system suggests mechanisms underlying COVID-19-associated anosmia. *bioRxiv*, 2020.2003.2025.009084 (2020).

141. Marcucci, F., Zou, D.J. & Firestein, S. Sequential onset of presynaptic molecules during olfactory sensory neuron maturation. *J Comp Neurol* **516**, 187-198 (2009).

142. Yang, R., Ma, H., Thomas, S.M. & Kinnamon, J.C. Immunocytochemical analysis of syntaxin-1 in rat circumvallate taste buds. *J Comp Neurol* **502**, 883-893 (2007).

143. van Swinderen, B. & Kottler, B. Explaining general anesthesia: a two-step hypothesis linking sleep circuits and the synaptic release machinery. *BioEssays : news and reviews in molecular, cellular and developmental biology* **36**, 372-381 (2014).

144. Jin, J. et al. Interrogation of brain miRNA and mRNA expression profiles reveals a molecular regulatory network that is perturbed by mutant huntingtin. *J Neurochem* **123**, 477-490 (2012).

145. Xie, J., Tong, Z., Guan, X., Du, B. & Qiu, H. Clinical Characteristics of Patients Who Died of Coronavirus Disease 2019 in China. *JAMA Netw Open* **3**, e205619 (2020).

146. Gattinoni, L. et al. COVID-19 pneumonia: different respiratory treatments for different phenotypes? *Intensive Care Med* **46**, 1099-1102 (2020).

147. Ottestad, W., Seim, M. & Mæhlen, J.O. COVID-19 with silent hypoxemia. *Tidsskr Nor Laegeforen* **140** (2020).

148. Wilkerson, R.G., Adler, J.D., Shah, N.G. & Brown, R. Silent hypoxia: A harbinger of clinical deterioration in patients with COVID-19. *Am J Emerg Med* **38**, 2243.e2245-2243.e2246 (2020).

149. López-Barneo, J., Ortega-Sáenz, P., Pardal, R., Pascual, A. & Piruat, J.I. Carotid body oxygen sensing. *Eur Respir J* **32**, 1386-1398 (2008).

150. Koerner, P., Hesslinger, C., Schaefermeyer, A., Prinz, C. & Gratzl, M. Evidence for histamine as a transmitter in rat carotid body sensor cells. *J Neurochem* **91**, 493-500 (2004).

151. Sasaki, A. et al. Molecular analysis of congenital central hypoventilation syndrome. *Human genetics* **114**, 22-26 (2003).

152. Lee, Y.I. et al. Dysregulation of the SNARE-binding protein Munc18-1 impairs BDNF secretion and synaptic neurotransmission: a novel interventional target to protect the aging brain. *Geroscience* **41**, 109-123 (2019).

153. Barrenschee, M. et al. SNAP-25 is abundantly expressed in enteric neuronal networks and upregulated by the neurotrophic factor GDNF. *Histochem Cell Biol* **143**, 611-623 (2015).

154. Chee, Y.J., Ng, S.J.H. & Yeoh, E. Diabetic ketoacidosis precipitated by Covid-19 in a patient with newly diagnosed diabetes mellitus. *Diabetes Res Clin Pract* **164**, 108166 (2020).

155. Li, J. et al. COVID-19 infection may cause ketosis and ketoacidosis. *Diabetes Obes Metab* **22**, 1935-1941 (2020).

156. Ren, H. et al. Association of the insulin resistance marker TyG index with the severity and mortality of COVID-19. *Cardiovasc Diabetol* **19**, 58 (2020).

157. Yang, J.K., Lin, S.S., Ji, X.J. & Guo, L.M. Binding of SARS coronavirus to its receptor damages islets and causes acute diabetes. *Acta Diabetol* **47**, 193-199 (2010).

158. Gaisano, H.Y. Deploying insulin granule-granule fusion to rescue deficient insulin secretion in diabetes. *Diabetologia* **55**, 877-880 (2012).

159. Südhof, T.C. & Rothman, J.E. Membrane fusion: grappling with SNARE and SM proteins. *Science (New York, N.Y.)* **323**, 474-477 (2009).

160. Liang, T. et al. New Roles of Syntaxin-1A in Insulin Granule Exocytosis and Replenishment. *J Biol Chem* **292**, 2203-2216 (2017).

161. Niu, M.J., Yang, J.K., Lin, S.S., Ji, X.J. & Guo, L.M. Loss of angiotensin-converting enzyme 2 leads to impaired glucose homeostasis in mice. *Endocrine* **34**, 56-61 (2008).

162. El Akoum, S., Haddad, Y. & Couture, R. Impact of pioglitazone and bradykinin type 1 receptor antagonist on type 2 diabetes in high-fat diet-fed C57BL/6J mice. *Obes Sci Pract* **3**, 352-362 (2017).

163. Haddad, Y. & Couture, R. Kininase 1 As a Preclinical Therapeutic Target for Kinin B(1) Receptor in Insulin Resistance. *Frontiers in pharmacology* **8**, 509 (2017).

164. Catanzaro, O.L., Dziubecki, D., Obregon, P., Rodriguez, R.R. & Sirois, P. Antidiabetic efficacy of bradykinin antagonist R-954 on glucose tolerance test in diabetic type 1 mice. *Neuropeptides* **44**, 187-189 (2010).

165. Seguin, T. et al. Hemodynamic and renal involvement of B1 and B2 kinin receptors during the acute phase of endotoxin shock in mice. *Int Immunopharmacol* **8**, 217-221 (2008).

166. Sales, V.M. et al. Kinin B(1) Receptor Acts in Adipose Tissue to Control Fat Distribution in a Cell-Nonautonomous Manner. *Diabetes* **68**, 1614-1623 (2019).

167. Belgardt, B.F. et al. The microRNA-200 family regulates pancreatic beta cell survival in type 2 diabetes. *Nat Med* **21**, 619-627 (2015).

168. Roshanravan, N. et al. The suppression of TXNIP and miR-200c improve beta-cell function in patients with Type 2 diabetes: A randomized, double-blind, placebo-controlled trial. *Journal of Functional Foods* **48**, 481-489 (2018).

169. Zhang, H. et al. Inhibition of miR-200c Restores Endothelial Function in Diabetic Mice Through Suppression of COX-2. *Diabetes* **65**, 1196-1207 (2016).

170. Singh, G.B. et al. MicroRNA-200c modulates DUSP-1 expression in diabetes-induced cardiac hypertrophy. *Mol Cell Biochem* **424**, 1-11 (2017).

171. Mao, Y., Lin, W., Wen, J. & Chen, G. Clinical and pathological characteristics of 2019 novel coronavirus disease (COVID-19): a systematic reviews. *medRxiv*, 2020.2002.2020.20025601 (2020).

172. Martínez-Alemán, S.R. et al. Understanding the Entanglement: Neutrophil Extracellular Traps (NETs) in Cystic Fibrosis. *Front Cell Infect Microbiol* **7**, 104 (2017).

173. Cutting, G.R. Cystic fibrosis genetics: from molecular understanding to clinical application. *Nat Rev Genet* **16**, 45-56 (2015).

174. Jena, B.P. Porosome in Cystic Fibrosis. *Discoveries (Craiova)* **2** (2014).

175. Ware, L.B. Physiological and biological heterogeneity in COVID-19-associated acute respiratory distress syndrome. *Lancet Respir Med* **8**, 1163-1165 (2020).

176. Wittekindt, O.H. Tight junctions in pulmonary epithelia during lung inflammation. *Pflugers Arch* **469**, 135-147 (2017).

177. Hong, J. Expression of Tight Junction Protein Occludin and ZO-1 in Lungs of COPD Rats. *Journal of Kunming Medical University* (2012).

178. Liu, M., Gu, C. & Wang, Y. Upregulation of the tight junction protein occludin: effects on ventilation-induced lung injury and mechanisms of action. *BMC Pulm Med* **14**, 94 (2014).

179. Liu, Y. et al. Unfractionated Heparin Alleviates Sepsis-Induced Acute Lung Injury by Protecting Tight Junctions. *J Surg Res* **238**, 175-185 (2019).

180. Vaziri, N.D., Yuan, J., Nazertehrani, S., Ni, Z. & Liu, S. Chronic kidney disease causes disruption of gastric and small intestinal epithelial tight junction. *Am J Nephrol* **38**, 99-103 (2013).

181. Lai, W.-T., Huang, Y.-H., Lo, M.-H. & Kuo, H.-C. Tight junction protein ZO-1 in Kawasaki disease. (2020).

182. Anderson, J.M. & Van Itallie, C.M. Physiology and function of the tight junction. *Cold Spring Harbor perspectives in biology* **1**, a002584 (2009).

183. Rahimi, N. Defenders and Challengers of Endothelial Barrier Function. *Front Immunol* **8**, 1847 (2017).

184. Mugisho, O.O., Robilliard, L.D., Nicholson, L.F.B., Graham, E.S. & O'Carroll, S.J. Bradykinin receptor-1 activation induces inflammation and increases the permeability of human brain microvascular endothelial cells. *Cell Biol Int* (2019).

185. Raslan, F. et al. Inhibition of bradykinin receptor B1 protects mice from focal brain injury by reducing blood-brain barrier leakage and inflammation. *J Cereb Blood Flow Metab* **30**, 1477-1486 (2010).

186. Kenne, E. et al. Neutrophils engage the kallikrein-kinin system to open up the endothelial barrier in acute inflammation. *FASEB journal : official publication of the Federation of American Societies for Experimental Biology* **33**, 2599-2609 (2019).

187. Davey, A., McAuley, D.F. & O'Kane, C.M. Matrix metalloproteinases in acute lung injury: mediators of injury and drivers of repair. *Eur Respir J* **38**, 959-970 (2011).

188. Hsu, A.T. et al. Kinetics and Role of Plasma Matrix Metalloproteinase-9 Expression in Acute Lung Injury and the Acute Respiratory Distress Syndrome. *Shock* **44**, 128-136 (2015).

189. Ueland, T. et al. Distinct and early increase in circulating MMP-9 in COVID-19 patients with respiratory failure. *J Infect* **81**, e41-e43 (2020).

190. Turner, R.J. & Sharp, F.R. Implications of MMP9 for Blood Brain Barrier Disruption and Hemorrhagic Transformation Following Ischemic Stroke. *Front Cell Neurosci* **10**, 56 (2016).

191. Brilha, S. et al. Matrix metalloproteinase-9 activity and a downregulated Hedgehog pathway impair blood-brain barrier function in an in vitro model of CNS tuberculosis. *Sci Rep* **7**, 16031 (2017).

192. Pulido-Olmo, H. et al. Role of matrix metalloproteinase-9 in chronic kidney disease: a new biomarker of resistant albuminuria. *Clin Sci (Lond)* **130**, 525-538 (2016).

193. Matus, C.E. et al. Activation of the human keratinocyte B1 bradykinin receptor induces expression and secretion of metalloproteases 2 and 9 by transactivation of epidermal growth factor receptor. *Exp Dermatol* **25**, 694-700 (2016).

194. Dunsmore, S.E. & Rannels, D.E. Extracellular matrix biology in the lung. *Am J Physiol* **270**, L3-27 (1996).

195. Vu, T.H. Don't mess with the matrix. *Nat Genet* **28**, 202-203 (2001).

196. Greenlee, K.J., Werb, Z. & Kheradmand, F. Matrix metalloproteinases in lung: multiple, multifarious, and multifaceted. *Physiol Rev* **87**, 69-98 (2007).

197. Vaisar, T. et al. MMP-9 sheds the beta2 integrin subunit (CD18) from macrophages. *Molecular & cellular proteomics : MCP* **8**, 1044-1060 (2009).

198. Li, C. et al. Development of atopic dermatitis-like skin disease from the chronic loss of epidermal caspase-8. *Proc Natl Acad Sci U S A* **107**, 22249-22254 (2010).

199. Symowicz, J. et al. Engagement of collagen-binding integrins promotes matrix metalloproteinase-9-dependent E-cadherin ectodomain shedding in ovarian carcinoma cells. *Cancer Res* **67**, 2030-2039 (2007).

200. Austinat, M. et al. Blockade of bradykinin receptor B1 but not bradykinin receptor B2 provides protection from cerebral infarction and brain edema. *Stroke* **40**, 285-293 (2009).

201. Göbel, K. et al. Blockade of the kinin receptor B1 protects from autoimmune CNS disease by reducing leukocyte trafficking. *J Autoimmun* **36**, 106-114 (2011).

202. Kassiri, Z. et al. Loss of angiotensin-converting enzyme 2 accelerates maladaptive left ventricular remodeling in response to myocardial infarction. *Circ Heart Fail* **2**, 446-455 (2009).

203. Elhelw, D.S. et al. Ectopic delivery of miR-200c diminishes hepatitis C virus infectivity through transcriptional and translational repression of Occludin. *Arch Virol* **162**, 3283-3291 (2017).

204. Wilson, S. et al. The membrane-anchored serine protease, TMPRSS2, activates PAR-2 in prostate cancer cells. *Biochem J* **388**, 967-972 (2005).

205. Su, X., Camerer, E., Hamilton, J.R., Coughlin, S.R. & Matthay, M.A. Protease-activated receptor-2 activation induces acute lung inflammation by neuropeptide-dependent mechanisms. *J Immunol* **175**, 2598-2605 (2005).

206. Kowluru, R.A., Santos, J.M. & Zhong, Q. Sirt1, a negative regulator of matrix metalloproteinase-9 in diabetic retinopathy. *Invest Ophthalmol Vis Sci* **55**, 5653-5660 (2014).

207. Herrero, R. et al. Fas activation alters tight junction proteins in acute lung injury. *Thorax* **74**, 69-82 (2019).

208. de Vries, N.A. et al. Prolonged Ezh2 Depletion in Glioblastoma Causes a Robust Switch in Cell Fate Resulting in Tumor Progression. *Cell Rep* **10**, 383-397 (2015).

209. Kling, K.M., Lopez-Rodriguez, E., Pfarrer, C., Mühlfeld, C. & Brandenberger, C. Aging exacerbates acute lung injury-induced changes of the air-blood barrier, lung function, and inflammation in the mouse. *Am J Physiol Lung Cell Mol Physiol* **312**, L1-l12 (2017).

210. Jones, V.G. et al. COVID-19 and Kawasaki Disease: Novel Virus and Novel Case. *Hosp Pediatr* **10**, 537-540 (2020).

211. Moreira, A. Kawasaki disease linked to COVID-19 in children. *Nat Rev Immunol* **20**, 407 (2020).

212. Nathan, N., Prevost, B. & Corvol, H. Atypical presentation of COVID-19 in young infants. *Lancet* **395**, 1481 (2020).

213. Riphagen, S., Gomez, X., Gonzalez-Martinez, C., Wilkinson, N. & Theocharis, P. Hyperinflammatory shock in children during COVID-19 pandemic. *Lancet* **395**, 1607-1608 (2020).

214. Verdoni, L. et al. An outbreak of severe Kawasaki-like disease at the Italian epicentre of the SARS-CoV-2 epidemic: an observational cohort study. *Lancet* **395**, 1771-1778 (2020).

215. Rivera-Figueroa, E.I., Santos, R., Simpson, S. & Garg, P. Incomplete Kawasaki Disease in a Child with Covid-19. *Indian Pediatr* **57**, 680-681 (2020).

216. Belhadjer, Z. et al. Acute Heart Failure in Multisystem Inflammatory Syndrome in Children in the Context of Global SARS-CoV-2 Pandemic. *Circulation* **142**, 429-436 (2020).

217. Toubiana, J. et al. Kawasaki-like multisystem inflammatory syndrome in children during the covid-19 pandemic in Paris, France: prospective observational study. *Bmj* **369**, m2094 (2020).

218. Rodríguez, Y. et al. Autoinflammatory and autoimmune conditions at the crossroad of COVID-19. *J Autoimmun* **114**, 102506 (2020).

219. Menter, T. et al. Postmortem examination of COVID-19 patients reveals diffuse alveolar damage with severe capillary congestion and variegated findings in lungs and other organs suggesting vascular dysfunction. *Histopathology* **77**, 198-209 (2020).

220. Papa, A., Salzano, A.M., Di Dato, M.T. & Varrassi, G. Images in Practice: Painful Cutaneous Vasculitis in a SARS-Cov-2 IgG-Positive Child. *Pain Ther* **9**, 805-807 (2020).

221. Dominguez-Santas, M. et al. Cutaneous small-vessel vasculitis associated with novel 2019 coronavirus SARS-CoV-2 infection (COVID-19). *J Eur Acad Dermatol Venereol* **34**, e536-e537 (2020).

222. Rivas, M.N.W., D.; Abe, M.; Franklin, M. K.; Chen, S.; Shimada, K.; Crother, T. R.; Arditi, M. in Circulation (2017).

223. Noval Rivas, M. et al. Intestinal Permeability and IgA Provoke Immune Vasculitis Linked to Cardiovascular Inflammation. *Immunity* **51**, 508-521.e506 (2019).

224. Kahn, R. et al. Microvesicle transfer of kinin B1-receptors is a novel inflammatory mechanism in vasculitis. *Kidney Int* **91**, 96-105 (2017).

225. Tharaux, P.L. & Dhaun, N. Endothelium-Neutrophil Communication via B1-Kinin Receptor-Bearing Microvesicles in Vasculitis. *J Am Soc Nephrol* **28**, 2255-2258 (2017).

226. Mossberg, M. et al. C1-Inhibitor Decreases the Release of Vasculitis-Like Chemotactic Endothelial Microvesicles. *J Am Soc Nephrol* **28**, 2472-2481 (2017).

227. Zhang, W., Wang, Y., Zeng, Y., Hu, L. & Zou, G. Serum miR-200c and miR-371-5p as the Useful Diagnostic Biomarkers and Therapeutic Targets in Kawasaki Disease. *Biomed Res Int* **2017**, 8257862 (2017).

228. Lopatko Fagerström, I. et al. Blockade of the kallikrein-kinin system reduces endothelial complement activation in vascular inflammation. *EBioMedicine* **47**, 319-328 (2019).

229. Takahashi, Y., Haga, S., Ishizaka, Y. & Mimori, A. Autoantibodies to angiotensin-converting enzyme 2 in patients with connective tissue diseases. *Arthritis Res Ther* **12**, R85 (2010).

230. Silva de Souza, A.W. Autoantibodies in systemic vasculitis. *Front Immunol* **6**, 184 (2015).

231. Uppal, N.N. et al. De Novo ANCA-Associated Vasculitis With Glomerulonephritis in COVID-19. *Kidney Int Rep* **5**, 2079-2083 (2020).

232. Sakurai, Y. Autoimmune Aspects of Kawasaki Disease. *J Investig Allergol Clin Immunol* **29**, 251-261 (2019).

233. Zhao, H., Shen, D., Zhou, H., Liu, J. & Chen, S. Guillain-Barré syndrome associated with SARS-CoV-2 infection: causality or coincidence? *Lancet Neurol* **19**, 383-384 (2020).

234. Camdessanche, J.P. et al. COVID-19 may induce Guillain-Barré syndrome. *Rev Neurol (Paris)* **176**, 516-518 (2020).

235. Dutra, R.C. et al. The role of kinin receptors in preventing neuroinflammation and its clinical severity during experimental autoimmune encephalomyelitis in mice. *PLoS One* **6**, e27875 (2011).

236. Langhauser, F. et al. Kininogen deficiency protects from ischemic neurodegeneration in mice by reducing thrombosis, blood-brain barrier damage, and inflammation. *Blood* **120**, 4082-4092 (2012).

237. Uchibori, A. & Chiba, A. [Autoantibodies in Guillain-Barré Syndrome]. *Brain Nerve* **67**, 1347-1357 (2015).

238. Flamier, A. et al. Modeling Late-Onset Sporadic Alzheimer's Disease through BMI1 Deficiency. *Cell Rep* **23**, 2653-2666 (2018).

239. Joshi, S. et al. Aging Healthy, or with Diabetes, is Associated with ACE2/ACE Imbalance in the Hematopoietic Stem Progenitor Cells. *The FASEB Journal* **33**, 514.517-514.517 (2019).

240. Comunian, S., Dongo, D., Milani, C. & Palestini, P. Air Pollution and Covid-19: The Role of Particulate Matter in the Spread and Increase of Covid-19's Morbidity and Mortality. *Int J Environ Res Public Health* **17** (2020).

241. Oakes, J.M., Fuchs, R.M., Gardner, J.D., Lazartigues, E. & Yue, X. Nicotine and the renin-angiotensin system. *Am J Physiol Regul Integr Comp Physiol* **315**, R895-r906 (2018).

242. Yue, X. et al. Nicotine Downregulates the Compensatory Angiotensin-Converting Enzyme 2/Angiotensin Type 2 Receptor of the Renin–Angiotensin System. *Ann Am Thorac Soc* **15**, S126-S127 (2018).

243. Aztatzi-Aguilar, O.G., Uribe-Ramírez, M., Arias-Montaño, J.A., Barbier, O. & De Vizcaya-Ruiz, A. Acute and subchronic exposure to air particulate matter induces expression of angiotensin and bradykinin-related genes in the lungs and heart: Angiotensin-II type-I receptor as a molecular target of particulate matter exposure. *Part Fibre Toxicol* **12**, 17 (2015).

244. Al Hariri, M. et al. Cigarette Smoking-Induced Cardiac Hypertrophy, Vascular Inflammation and Injury Are Attenuated by Antioxidant Supplementation in an Animal Model. *Frontiers in pharmacology* **7**, 397 (2016).

245. Lin, J.C. et al. Mechanism of cigarette smoke-induced kinin B(1) receptor expression in rat airways. *Peptides* **31**, 1940-1945 (2010).

246. Kumari, K. et al. Nicotine associated breast cancer in smokers is mediated through high level of EZH2 expression which can be reversed by methyltransferase inhibitor DZNepA. *Cell death & disease* **9**, 152 (2018).

247. Vaz, M. et al. Chronic Cigarette Smoke-Induced Epigenomic Changes Precede Sensitization of Bronchial Epithelial Cells to Single-Step Transformation by KRAS Mutations. *Cancer Cell* **32**, 360-376.e366 (2017).

248. Anzalone, G. et al. Cigarette smoke affects the onco-suppressor DAB2IP expression in bronchial epithelial cells of COPD patients. *Sci Rep* **9**, 15682 (2019).

249. Wang, X. et al. (Preprints.org, 2020).

250. Zhang, J.J. et al. Clinical characteristics of 140 patients infected with SARS-CoV-2 in Wuhan, China. *Allergy* **75**, 1730-1741 (2020).

251. Brüssow, H. The Novel Coronavirus - A Snapshot of Current Knowledge. *Microb Biotechnol* **13**, 607-612 (2020).

252. Chen, N. et al. Epidemiological and clinical characteristics of 99 cases of 2019 novel coronavirus pneumonia in Wuhan, China: a descriptive study. *Lancet* **395**, 507-513 (2020).

253. Huang, C. et al. Clinical features of patients infected with 2019 novel coronavirus in Wuhan, China. *Lancet* **395**, 497-506 (2020).

254. News, C.R. (2020).

255. Chen, J. et al. Individual variation of the SARS-CoV-2 receptor ACE2 gene expression and regulation. *Aging Cell* **19** (2020).

256. Xie, X., Chen, J., Wang, X., Zhang, F. & Liu, Y. Age- and gender-related difference of ACE2 expression in rat lung. *Life Sci* **78**, 2166-2171 (2006).

257. Corley, M.J.N., L.C. DNA Methylation Analysis of the COVID-19 Host Cell Receptor, Angiotensin I Converting Enzyme 2 Gene (ACE2) in the Respiratory System Reveal Age and Gender Differences. *Preprints* (2020).

258. Fan, R. et al. Preliminary analysis of the association between methylation of the ACE2 promoter and essential hypertension. *Mol Med Rep* **15**, 3905-3911 (2017).

259. Tukiainen, T. et al. Landscape of X chromosome inactivation across human tissues. *Nature* **550**, 244-248 (2017).

260. Milsted, A. et al. Regulation of multiple renin-angiotensin system genes by Sry. *J Hypertens* **28**, 59-64 (2010).

261. Bukowska, A. et al. Protective regulation of the ACE2/ACE gene expression by estrogen in human atrial tissue from elderly men. *Exp Biol Med (Maywood)* **242**, 1412-1423 (2017).

262. Cyr, M. et al. Bradykinin and des-Arg(9)-bradykinin metabolic pathways and kinetics of activation of human plasma. *Am J Physiol Heart Circ Physiol* **281**, H275-283 (2001).

263. Hwang, J.W., Yao, H., Caito, S., Sundar, I.K. & Rahman, I. Redox regulation of SIRT1 in inflammation and cellular senescence. *Free Radic Biol Med* **61**, 95-110 (2013).

264. Han, X. & Sun, Z. Epigenetic Regulation of KL (Klotho) via H3K27me3 (Histone 3 Lysine [K] 27 Trimethylation) in Renal Tubule Cells. *Hypertension* **75**, 1233-1241 (2020).

265. Dozmorov, M.G. Polycomb repressive complex 2 epigenomic signature defines age-associated hypermethylation and gene expression changes. *Epigenetics* **10**, 484-495 (2015).

266. Yang, D. et al. Autophagy in diabetic kidney disease: regulation, pathological role and therapeutic potential. *Cell Mol Life Sci* **75**, 669-688 (2018).

267. Waldman, M. et al. Regulation of diabetic cardiomyopathy by caloric restriction is mediated by intracellular signaling pathways involving 'SIRT1 and PGC-1α'. *Cardiovasc Diabetol* **17**, 111 (2018).

268. Bramante, C. et al. Observational Study of Metformin and Risk of Mortality in Patients Hospitalized with Covid-19. *medRxiv*, 2020.2006.2019.20135095 (2020).

269. Zhang, R. et al. COVID-19: Melatonin as a potential adjuvant treatment. *Life Sci* **250**, 117583 (2020).

270. (2020).

271. Filardo, S., Di Pietro, M., Mastromarino, P. & Sessa, R. Therapeutic potential of resveratrol against emerging respiratory viral infections. *Pharmacol Ther* **214**, 107613 (2020).

272. Patel, V.B. et al. ACE2 Deficiency Worsens Epicardial Adipose Tissue Inflammation and Cardiac Dysfunction in Response to Diet-Induced Obesity. *Diabetes* **65**, 85-95 (2016).

273. Mariani, S. et al. Inverse Association of Circulating SIRT1 and Adiposity: A Study on Underweight, Normal Weight, and Obese Patients. *Frontiers in endocrinology* **9**, 449 (2018).

274. Mori, M.A. et al. Kinin B1 receptor in adipocytes regulates glucose tolerance and predisposition to obesity. *PLoS One* **7**, e44782 (2012).

275. Morais, R.L. et al. Kinin B1 and B2 receptor deficiency protects against obesity induced by a high-fat diet and improves glucose tolerance in mice. *Diabetes Metab Syndr Obes* **8**, 399-407 (2015).

276. Corder, E.H. et al. Gene dose of apolipoprotein E type 4 allele and the risk of Alzheimer's disease in late onset families. *Science (New York, N.Y.)* **261**, 921-923 (1993).

277. Kuo, C.L. et al. APOE e4 Genotype Predicts Severe COVID-19 in the UK Biobank Community Cohort. *J Gerontol A Biol Sci Med Sci* **75**, 2231-2232 (2020).

278. Montagne, A. et al. APOE4 leads to blood-brain barrier dysfunction predicting cognitive decline. *Nature* **581**, 71-76 (2020).

279. Liu, C., von Brunn, A. & Zhu, D. Cyclophilin A and CD147: novel therapeutic targets for the treatment of COVID-19. *Med Drug Discov* **7**, 100056 (2020).

280. Ellinghaus, D. et al. Genomewide Association Study of Severe Covid-19 with Respiratory Failure. *N Engl J Med* **383**, 1522-1534 (2020).

281. Singer, D.C., S. M. R. Collectrin and ACE2 in renal and intestinal amino acid transport. *Channels* **5**, 410-423 (2011).

282. Xie, X. et al. Associations of SLC6A20 genetic polymorphisms with Hirschsprung's disease in a Southern Chinese population. *Bioscience reports* **39** (2019).

283. Ellul, M.A. et al. Neurological associations of COVID-19. *Lancet Neurol* **19**, 767-783 (2020).

284. Kormann, R. et al. Coronavirus disease 2019: acute Fanconi syndrome precedes acute kidney injury. *Clinical Kidney Journal* **13**, 362-370 (2020).

285. Chu, P.L. & Le, T.H. Role of collectrin, an ACE2 homologue, in blood pressure homeostasis. *Curr Hypertens Rep* **16**, 490 (2014).

286. Thomas, T. et al. COVID-19 infection results in alterations of the kynurenine pathway and fatty acid metabolism that correlate with IL-6 levels and renal status. *medRxiv* (2020).

287. (2020).

288. Qi, Y. et al. Diminazene aceturate enhances angiotensin-converting enzyme 2 activity and attenuates ischemia-induced cardiac pathophysiology. *Hypertension* **62**, 746-752 (2013).

289. Scroggin, M.P., K. M.; Lazatigues, E. The PPAR-γ agonist Rosiglitazone increases angiotensin-converting enzyme 2 (ACE2) promoter activity in neurons. *The FASEB Journal* **26**, 875.813 (2012).

290. Shah, S.A. et al. Melatonin Stimulates the SIRT1/Nrf2 Signaling Pathway Counteracting Lipopolysaccharide (LPS)-Induced Oxidative Stress to Rescue Postnatal Rat Brain. *CNS Neurosci Ther* **23**, 33-44 (2017).

291. Luo, P. et al. Metformin Treatment Was Associated with Decreased Mortality in COVID-19 Patients with Diabetes in a Retrospective Analysis. *Am J Trop Med Hyg* **103**, 69-72 (2020).

292. Horby, P. et al. Dexamethasone in Hospitalized Patients with Covid-19 - Preliminary Report. *N Engl J Med* (2020).

293. Phagoo, S.B., Reddi, K., Silvallana, B.J., Leeb-Lundberg, L.M. & Warburton, D. Infection-induced kinin B1 receptors in human pulmonary fibroblasts: role of intact pathogens and p38 mitogen-activated protein kinase-dependent signaling. *J Pharmacol Exp Ther* **313**, 1231-1238 (2005).

294. Liu, J. et al. AMPK: a balancer of the renin-angiotensin system. *Bioscience reports* **39** (2019).

295. Hernández Prada, J.A. et al. Structure-based identification of small-molecule angiotensin-converting enzyme 2 activators as novel antihypertensive agents. *Hypertension* **51**, 1312-1317 (2008).

296. Chen, X. et al. Heme Oxygenase-1 Reduces Sepsis-Induced Endoplasmic Reticulum Stress and Acute Lung Injury. *Mediators Inflamm* **2018**, 9413876 (2018).

297. He, Z. et al. HO-1 promotes resistance to an EZH2 inhibitor through the pRB-E2F pathway: correlation with the progression of myelodysplastic syndrome into acute myeloid leukemia. *J Transl Med* **17**, 366 (2019).

298. Cao, Q. et al. Coordinated regulation of polycomb group complexes through microRNAs in cancer. *Cancer Cell* **20**, 187-199 (2011).

299. van der Lugt, N.M. et al. Posterior transformation, neurological abnormalities, and severe hematopoietic defects in mice with a targeted deletion of the bmi-1 proto-oncogene. *Genes Dev* **8**, 757-769 (1994).

300. Ghebrehiwet, B., Geisbrecht, B.V., Xu, X., Savitt, A.G. & Peerschke, E.I.B. The C1q Receptors: Focus on gC1qR/p33 (C1qBP, p32, HABP-1)(1). *Semin Immunol* **45**, 101338 (2019).

301. Hill, N. et al. Glomerular endothelial derived vesicles mediate podocyte dysfunction: A potential role for miRNA. *PLoS One* **15**, e0224852 (2020).

302. Ottaviani, L.M.J., R. P.; Sansonetti, M.; Sampaio-Pinto, V.; Halkein, J.; el Azzouzi, H.; Olieslagers, S., Nascimento, D. S.; de Windt, L. J.; da Costa Martins, P. A.; in Circulation Research, Vol. 125 (2019).

303. Moreno-Manzano, V. et al. Retinoids as a potential treatment for experimental puromycin-induced nephrosis. *Br J Pharmacol* **139**, 823-831 (2003).

304. Dambha-Miller, H. et al. Currently prescribed drugs in the UK that could upregulate or downregulate ACE2 in COVID-19 disease: a systematic review. *BMJ Open* **10**, e040644 (2020).
