# Supplementary material for: Whole patient knowledge modeling of COVID-19 symptomatology reveals common molecular mechanisms
Source: Front Mol Med. 2023 Jan 4;2:1035290. doi: 10.3389/fmmed.2022.1035290 (PMC11285600; doi:10.3389/fmmed.2022.1035290)
Supplement: Supplementary file 6 [file Table1.DOCX]

**Tables**

**Table 1:** **The COVID-19 Cockpit.**

Summary of the reported COVID-19 clinical phenotypes, pathogenic mechanisms and the relation to the COVID-19 disease model for each of the SARS-CoV-2 perturbed eight key mechanisms. A link to the COVID-19 Explorer is provided at the beginning of each section.

| **COVID-19 Cockpit** | | | |  |
| --- | --- | --- | --- | --- |
| **Exocytosis** (Supplement Fig. 12, 3.3.1)  (https://covid19.molecularhealth.com/t/submodels/55.html) | | | |  |
| **COVID-19 Phenotype** | **Molecular Pathology** | | **Relation to Model and Findings** | **Validation of Predictions** |
| **Neurological Dysfunction**  Cognitive deficits equivalent to >10 years ageing**^1, 2^** | - Dysregulation of the frontotemporal **SNARE** interactome is a cause of age-related cognitive decline^3^. - Global cognitive decline is associated with reduced SNARE complex levels (**Syntaxin1**, **SNAP25**, **VAMP**) and early synapse dysfunction independent of synapse loss^4, 5^. | | - Synaptic impairment due to **B1R**/**miR200c** induced repression of **Syntaxin1**. - Virus-independent propagation of symptomatology due to microvesicle (MV) based disease mechanism. |  |
| **Anosmia / Ageusia**  Changes in smell and taste as well as complete anosmia and ageusia are prominent and specific symptoms of COVID-19^6-9^. | - The synaptic release machinery of olfactory sensory neurons is centered on the **Syntaxin1** dependent **SNARE** complex^10^. - Taste cell synapses use the classical **Syntaxin1** **SNARE** machinery for neurotransmitter release in circumvallate taste buds^11^. | | - Olfactory sensory neurons express neither **ACE2** nor **TMPRSS2**. - Epithelial support cells, cells in the nasal epithelium, stem cells, epithelial cells of oral mucosa, in fungiform and circumvallate papillae express both of these genes^12-14^. - MVs carrying **miR200c** are transferred from infected cells to sensory cells upon excess des-Arg-kinin (DAK) and **B1R** activation may lead to suppression of **Syntaxin1**. |  |
| **Silent Hypoxemia**  Severe hypoxemia associated with near normal respiratory system compliance^15^. | - Similar to congenital central hypo-ventilation syndrome (CCHS), a life-threatening disorder with impaired ventilatory response to hypoxia caused by carotid body (CB) dysfunction. - CCHS patients show a significant decrease of dopaminergic vesicles in oxygen sensor cells of CBs^16^. - Sensor cells of the CB utilize the exocytotic apparatus and its components **SNAP25** and **Syntaxin1** for signaling^17^. | | - CB sensor cells are centrally exposed to circulating virus particles. - **B1R**/**miR200c** mediated downregulation of **Syntaxin1** may induce impairment of the exocytotic machinery in CB sensor cells causing silent hypoxemia. | - COVID-19 affects the CB even in the absence of its direct invasion^18^ |
| **New-onset Diabetes**  New-onset diabetes and severe metabolic complications of preexisting diabetes (e.g. ketoacidosis and hyperosmolarity) have been observed in COVID-19 patients^19-21^. | - SERPING1 is down-regulated, while KNG1 is upregulated in T2D^22^ - Pancreatic islet β-cells release insulin via exocytosis of insulin secretory granules. - Exocytosis is mediated by the SNARE complex (**Syntaxin1**, **SNAP25** and VAMP)^23, 24^. - **Syntaxin1** plays a key role in Insulin granule exocytosis and replenishment^23^. - **ACE2** **deficiency** has been directly linked to defects in insulin secretion^25^. - **B1R** as well as **CPN1**, the enzyme that converts bradykinin (BK) into the **B1R** ligand **DABK**^26, 27^. **B1R** antagonism and blockade of **CPN1**^27-29^. - **B1R** sensitizes **PKCε**^30^ and B1R activation causes translocation of **PKCε**^31^ while **PKCε** contributes to lipid-induced insulin resistance^32^ - **B1R** in adipose tissue controls the response to diet-induced obesity, its deletion protects from diet-induced obesity and improves insulin sensitivity^33^. - **miR200c** is biomarker of insulin resistance in obesity^34^. - **miR200c** diminishes insulin production by inducing pancreatic β-cell damage^35^. - Suppression of **miR200c** improves β-cell function in patients with T2D and restores endothelial function in diabetic mice^36^. - **miR200c** down-regulates **IRS1^37^**. In diabetes, **IRS1** down-regulation is associated with insulin resistance^38^. - **miR200c** also targets **PGC1A**^39^, **PGC1A** controls the hepatic ratio of **IRS1** and IRS2 and in type 2 diabetes, low **PGC1A** is associated with insulin resistance^40^, in addition **TRPV4** (which is sensitized by **B1R** activation^30^) negatively regulates the **PGC1A** expression^41^ and **TRPV4** antagonists reduce high‐fat diet‐induced obesity, insulin resistance, diabetic nephropathy, retinopathy and neuropathy^41-44^. It has been demonstrated that **TRPV4** activity is mediated via the **B1R**-**PKCε** axis^30^. - **SERPING1** is down-regulated, while **KNG1** is upregulated in T2D^22^.      - Exocytosis is mediated by the SNARE complex (**Syntaxin1**, **SNAP25** and VAMP)^23, 24^. - **Syntaxin1** plays a key role in Insulin granule exocytosis and replenishment^23^. - **ACE2** **deficiency** has been directly linked to defects in insulin secretion^25^. - **B1R** as well as **CPN1**, the enzyme that converts bradykinin (BK) into the **B1R** ligand **DABK**^26, 27^. **B1R** antagonism and blockade of **CPN1**^27-29^. - B1R sensitizes ^30^ and B1R activation causes translocation of^31^ while PKCε contributes to lipid-induced insulin resistance^32^ - **B1R** in adipose tissue controls the response to diet-induced obesity, its deletion protects from diet-induced obesity and improves insulin sensitivity^33^. - **miR200c** is biomarker of insulin resistance in obesity^34^. - **miR200c** diminishes insulin production by inducing pancreatic β-cell damage^35^. - Suppression of **miR200c** improves β-cell function in patients with T2D and restores endothelial function in diabetic mice^36^. - **miR200c** down-regulates **IRS1^37^**. In diabetes, **IRS1** down-regulation is associated with insulin resistance^38^. - **miR200c** also targets **PGC1A**^39^, **PGC1A** controls the hepatic ratio of **IRS1** and IRS2 and in type 2 diabetes, low **PGC1A** is associated with insulin resistance^40^, in addition **TRPV4** (which is sensitized by **B1R** activation^30^) negatively regulates the **PGC1A** expression^41^ and **TRPV4** antagonists reduce high‐fat diet‐induced obesity, insulin resistance, diabetic nephropathy, retinopathy and neuropathy^41-44^. It has been demonstrated that **TRPV4** activity is mediated via the **B1R**-**PKCε** axis^30^. - **SERPING1** is down-regulated, while **KNG1** is upregulated in T2D^22^. | | - **ACE2** deficiency and **B1R**/**miR200c** mediated downregulation of **Syntaxin1** in β-cells may lead to the induction of new-onset diabetes which can persist after virus clearance. - In addition **insulin resistance** may be induced by **B1R** mediated sensitizazion of **PKCε** and **miR200c** mediated downregulation of **IRS1. miR200c** also down-regulates **PCG1A**, which is associated with insulin resistance. PCG1A is also downmodulated by **TRPV4,**which in turn is sensitized by **B1R** activity. | - Islet miR200c levels are linked to diabetes^45^ - miR200c is a predictor of severity independent of COVID-19 risk factors^46^ |
| **Thick Mucus**  Mucous secretions of COVID-19 patients are reminiscent of those in cystic fibrosis (CF) patients^47^. | - CF is caused by mutations that disrupt the **CFTR** gene^48^. - **CFTR** is known to interact with **Syntaxin1**, chloride channel **CLC3**, and **aquaporins** to form the porosome complex^49^. - The process of secretion via the **porosome** is similar to exocytosis: a pore is formed through transient fusion of a secretory vesicle at the porosome base via **SNARE** proteins, resulting in a fusion pore^50^. | | - B1R/miR200c mediated downregulation of Syntaxin1 in lung epithelium may therefore mimic the molecular pathology of CF. |  |
| **Senescence** (Supplement Fig. 13)  (https://covid19.molecularhealth.com/t/submodels/46.html) | | | | - **Virus-induced senescence is a driver and therapeutic target in COVID-19^51^** - **Expression of the senescence marker CDKN2A increased in the pulmonary cells of patients with severe post-acute COVID-19^52^** |
| **COVID-19 Phenotype** | **Molecular Pathology** | | **Relation to Model and Findings** |  |
| **Objective muscle weakness**  Muscle weakness represents one of the most typical symptoms of COVID-19^53^. | - **ACE2** is expressed in muscle cells^54^. - **ACE2** deletion leads to early manifestation of aging-associated muscle weakness along with increased expression of **p16INK4a**^55, 56^. - Induction of senescence via de-repression of **p16INK4a** in muscle stem cells is responsible for age-related decline of the regeneration capacity of muscle tissue^57^. - Activation of the **p16INK4a** senescence pathway during aging breaks muscle homeostasis and causes degenerative muscle disease^58^. - In **ACE2** knockout mice **p16INK4a** is upregulated in skeletal muscles^59^. | | - Virus induced down-regulation of **ACE2** may induce objective muscle weakness through induction of a senescence pathway. - **P16INK4a** is a universal senescence marker and accumulates with age and inflammation^60^. - **BMI1** is a transcriptional repressor of **p16INK4a**^61^. - **BMI1** is a direct target of **miR200c**^62^. - **miR200c** has been shown to repress **BMI1**^63^ |  |
| **Lymphopenia**  T-cell senescence and lymphopenia has been reported in patients with COVID-19 pneumonia^64^. | - **BMI1**-depleted mice show defects in self-renewal of hematopoietic stem cells. - Overexpression of **BMI1** via repression of **p16INK4a** enables stem cells overcome senescence^65-67^. - **BMI1** regulates lymphoid specification by preventing B-cell lineage commitment. **BMI1** depletion in T-cells leads to T-to-B cell conversion^67^. | | - Virus induced overactivation of the **ACE2/DAK/B1R** axis via **miR200c** mediated downregulation of **BMI1** may contribute to the development of lymphopenia. |  |
| **Tissue repair deficiency**  The differentiation of mesenchymal stem cells (MSCs) into type-II alveolar epithelial cells is critical for re-epithelization and recovery in ARDS^68^.  Cellular senescence is a mediator of COVID-19 severity^69^. | - Endothelial progenitor cells (EPCs) and MSCs augment tissue repair. Due to their immunomodulatory properties MSCs were extensively used in regenerative medicine^70, 71^. - MSCs were recently used to treat COVID-19^72^. - In MSCs, knockdown of **BMI1** reduces self-renewal by upregulation of **p16INK4a**^73^. - **BMI1** induces immunosuppressive properties and senescence-control mechanisms in human MSCs by directly suppressing **DUSP1**^74^. - **p16INK4a** triggers senescence in EPCs. The onset is accelerated by administration of **Kininogen**^75^. - **ACE2** priming of MSCs and EPCs enhances their therapeutic efficacy^76, 77^. - Activation of **SIRT1** supports MSC mediated repair. This effect is abolished by **ACE2** inhibiton^78^. | | - Virus-induced overactivation of the **ACE2/DAK/B1R** axis via **miR200c** mediated downregulation of **BMI1** may contribute to stem cell senescence. |  |
| **Alopecia**  One of the striking long-term symptoms of COVID-19 survivors is hair loss**^79, 80^** | - Dermal Papilla Cells (DPCs) play a key role in normal hair growth and are considered as a reservoir of multipotent stem cells^81^. - Balding DPCs were linked to premature senescence as they lose proliferative capacity, express senescence markers and show decreased expression of **BMI1** together with upregulation of **p16INK4a**^82^. | | - The mechanism through which **B1R** overactivation contributes to **p16INK4a** expression may provide a direct link between our model and the observed alopecia in COVID-19 patients. |  |
| **Inflammatory Signaling** (Supplement Fig. 9)  (https://covid19.molecularhealth.com/t/submodels/55.html) | | | |  |
| **COVID-19 Phenotype** | **Molecular Pathology** | | **Relation to Model and Findings** |  |
| **Cytokine/Chemokine Syndromes**  (Hyperinflammation)  COVID-19 is characterized by immune cell infiltration and cytokine storm^83^. | - **ACE2** is anti-inflammatory:   **ACE2** activation reduces levels of **IL6**, **IL8**, **IL2**, and **CCL2**, **TNFα**, **IL1β** via downmodulation of **NFκB**, **MAPK**, **JAK**/**STAT** and **TGFβ** pathways and increases anti-inflammatory **IL10**^84-89^. Phosphorylation of **ACE2** by **AMPK** increases its anti-inflammatory activity. - **B1R** activation mediates the pro-inflammatory effect of **ACE2** downregulation^90^.  **B1R** induces chemokines **CXCL1**, **CXCL2**, **CXCL5**^90-92^, release of **MMP9** and **MPO** via **MAPK**^93^  and increase of **phosphatidylinositol** hydrolysis, **arachidonic acid** and **eicosanoid**^94, 95^. - **miR200c** induces **IL6** and **TNFα** ^96^. Knockdown of **miR200c** represses **TGFβ**, **TNFα**, and **IL1α** via inhibiting **NFκB** and **Smad2** activation^97^. | | - Downregulation of **ACE2** leads to over-activation of **B1R** and induction of **miR200c** leading to an increase in pro-inflammatory and decrease in anti-inflammatory cytokines. - The increase in chemokines induces neutrophil infiltration, activation and degranulation. |  |
| **Oxidative Stress Syndromes**  COVID-19 is associated with Reactive Oxygen Species (ROS) inflammation^98^ (e.g., ARDS). | - **miR200c** is induced by oxidative stress and highly elevated in plasma from pneumonia patients^99, 100^. **miR200c** represses antioxidant proteins **eNOS**, **catalase**, **HO1** and **SOD2**^99, 101^. - **B1R** activation induces **iNOS**. | | - **B1R** and **miR200c** lead to a decrease in antioxidant enzymes and increase in **ROS** formation. - In virus-induced lung injury concurrent downregulation of **eNOS** and upregulation of **iNOS** drive **ROS** production^102^. - **ROS** lead to ARDS progression and endothelial/epithelial barrier dysfunction^103-105^. |  |
| **Neurogenic Inflammation**  COVID-19 is associated with neurogenic inflammation^106^. | - **B1R** activation induces the release of **SubstanceP** and **CGRP**, leading to leukocyte recruitment^91, 92^. - **B1R** induces **TRPV1**^107^, a mediator of neurogenic inflammation^108^. - **ROS** activate **TRPV1** leading to overproduction of **SubstanceP** ^109^. | | - Morbidity and severity of COVID-19 are linked to the **TRPV1** expressing neuronal system in the lungs^110^. - Respiratory viral infections can upregulate **TRPV1** receptors^111^. - **CGRP** released from the **TRPV1** nerve endings downregulates innate immune responses^112^. - Neurogenic inflammation is involved in the molecular pathology of Migraine^113^. Migraine-like headache and light-sensitivity may occur in COVID-19^114^. |  |
| **Nociception** (Supplement Fig. 10)  (https://covid19.molecularhealth.com/t/submodels/47.html) | | | |  |
| **COVID-19 Phenotype** | **Molecular Pathology** | | **Relation to Model and Findings** |  |
| **Cough, Myalgia, Arthralgia and inflammatory pain**  Cough, myalgia and arthralgia are common symptoms of COVID-19^115^. | - **B1R** via activation of **TRPV1** is directly involved in acute inflammatory pain signaling, linking inflammation to nociception^116, 117^. - **TRPV1** activation elicits the cough reflex^118, 119^. - **B1R** activation mediates inflammatory muscle pain^120^. It triggers release of **NO**, **SubstanceP** and **CGRP**, key mediators of myalgia^121^ and diabetic hyperalgesia^122^. - **DABK** aggravates hyperalgesia, which is reversed by **B1R** antagonism^123^. **B1R** antagonism also reverses diabetic hyperalgesia by reducing **TRPV1** expression^107^. - **TRPV1** expression depends on **PKC** mediated phosphorylation^124^, consistent with the finding that in nociception **B1R** activates **PKC**^125^. - **TRPV1** mediates arthralgia^126^ and kinin-induced pain signaling in cutaneous nociceptors, cardiac afferents, jejunal afferents and tracheobronchial afferents^127-130^. - **TRPV4** (which is  sensitized by **B1R**^30^) mediates mechanical hyperalgesia via the **B1R**-**PKCε** axis^30^. | | - **B1R** activation is directly linked to nociception and inflammatory pain. It is co-expressed with **CGRP** and **TRPV1** on sensory C-^117, 131^ and A-fibers^132^. |  |
| **Dysgeusia**  Dysgeusia is a common COVID-19 symptom^133^. | - **B1R** is expressed in the sensory nervous system^134^  and peripheral sensory ganglia^135^. - **B1R** triggers release of **CGRP** and **SubstanceP** from trigeminal afferents, which terminate within taste buds, impacts taste-cell sensitivity and shapes taste signals^136^. - In peripheral terminals of the glossopharyngeal nerves **CGRP** release reduces taste signals before their transmission to the gustatory sensory fibers and the brain^137^. - **TRPV1** activation directly induces taste suppression^120^ and elicits the metallic aftertaste of artificial sweeteners^138^. - Metallic taste sensation was reported by COVID-19 patients^139^. | | - **B1R** activation by inducing **TRPV1** is directly linked to neurogenic signaling inducing dysgeusia. |  |
| **Ventilator induced acute lung injury** (VILI)  Reported mortality rates among COVID-19 patients requiring mechanical ventilation range from 50–97%^140-144^. VILI is one of the major risk factors^145^. | - VILI is triggered by mechano-sensation^146^. - The mechano-sensor **TRPV4**, shares 50% sequence homology with **TRPV1**. - **TRPV4** is involved in sour-taste sensing^147^, and plays a critical role in VILI^148^. - **TRPV4** is expressed in pulmonary bronchiolar and alveolar epithelia, alveolar macrophages and endothelium^149, 150^. - As **TRPV1**, **TRPV4** is sensitized via **PKC**^151^  and **B1R** agonism and **TRPV4** channels induce mechanical hyperalgesia in a **PKC**-dependent manner^30^. | | - **B1R** activation via mechanosensory **TRPV4** may induce VILI. - VILI is also associated with downregulation of TJ protein **Occludin. Occludin** expression is regulated by **miR200c** and **B1R** (see barrier permeability) | - In vitro screening identifies TRPV4 as target for endothelial barrier stabilization in COVID-19^152^ |
| **Coagulation**  (https://covid19.molecularhealth.com/t/submodels/50.html) | | | |  |
| **COVID-19 Phenotype** | **Molecular Pathology** | | **Relation to Model and Findings** |  |
| **Thrombo-embolic** **events** and **disseminated intravascular coagulopathy** (DIC)  Severe COVID-19 is often associated with thrombo-embolic events and DIC^153^. Abnormal coagulation is associated with poor prognosis and non‐survivors show higher levels of D‐dimer and fibrin degradation products^154^. | - **B1R** plays an important role in mediating organ damage and lethal thrombosis in septic shock^155^. - Increased expression of **B1R** predisposes platelets to thrombus formation^155^. - **B1R** activation results in sustained elevation of **intracellular Ca^2+^**^156^, which is essential for platelet activation in thrombosis^157^. - **B1R** inhibition like **Kininogen** deficiency protects from thrombosis^158^. Kininogen deficiency also protects from vascular injury^159^  and ischemic neurodegeneration by reducing thrombosis^158^. - **Fibrin degradation products** resulting from thrombotic events increase **DABK** generation^160^. - **ACE2** attenuates thrombus formation^161^  likely by eliminating **DAKs.** Low **ACE2/ACE** ratios observed in acute pulmonary embolism^162^. - SARS-CoV induced downregulation of **SERPING1** directly induces the coagulation cascade^163^ - SARS-CoV-2 infection stimulates formation of extracellular neutrophils traps (NETosis) which contributes to COVID-19 associted thrombosis^164^. - Non-lytic NETosis involves neutrophil degranulation and triggers a process in which MPO synergizes with neutrophil elastase in decondensing chromatin to form the neutrophil extracellular traps^165^. - NET bound histones mediate NET-dependent cytotoxicity^166^. - Histones and MMP9 contribute to NET-mediated barrier disruption^167, 168^,. - Inhibition of C1 is able to dose-dependently inhibit NET formation^169^. - Anti-histone properties of C1-Inhibitor SERPING1 protects against histone mediated lung injury^170^. - SARS-Cov2 induced downregulation od SERPING1 could contribute to COVID-19 associated NETosis. - B1R activation leads to degranulation of neutrophils and release of MMP9 and MPO^93^. - B1R activation may contribute to NETosis onset and sustain. | | - While downregulation of **SERPING1** directly activates the coagulation cascade, **ACE2** deficiency and **B1R** activation play a direct role in platelet activation. - Downregulation of SERPING1 and activation of B1R could directly contribute to NETosis formation and NET dependent cytotoxicity and thrombosis. |  |
| **Barrier Permeability** (Supplement Fig. 11)  (https://covid19.molecularhealth.com/t/submodels/56.html) | | | |  |
| **COVID-19 Phenotype** | **Molecular Pathology** | | **Relation to Model and Findings** |  |
| **Acute Respiratory Distress Syndrome** (ARDS)  One of the most common causes of hospital admission and death in patients with COVID-19 is ARDS characterized by acute lung inflammation and increased-permeability pulmonary edema^171^. Increased barrier permeability in alveolar edema, ARDS and acute lung injury (ALI) result from loss of tight junctions (TJ) permselectivity^172^, which coincides with upregulation of inflammatory cytokines. | - **B1R** stimulation results in the loss of **Occludin** TJs and an increase of vascular permeability^173^. In neuroinflammation **B1R** contributes to the loss of blood-brain-barrier integrity^174^. - Neutrophils engage the **KKS** to open the endothelial barrier in acute inflammation^175^. - Activation of **B1R** induces expression and secretion of **MMP9** and **MMP2**^176^. Active **MMP2** and **MMP9** degrade components of the alveolar basement membrane^177, 178^, non-matrix components such as integrins^179, 180^, and intercellular targets such as E-cadherin^181, 182^. - **B1R** blockade reduces edema formation in  ARDS^183^, acute ischemic stroke^184^, traumatic brain injury^174^  and multiple sclerosis^185^. - **ACE2** deficiency is associated with increased **MMP9** levels in myocardial infarction^186^. - miR200c represses Occludin^187^. Antago-miR200c inhibits MMP9 and increases Occludin^188^.      - **TMPRSS2** cleaves and activates **PAR2**^189^. In airways **PAR2** activation increases lung vascular and epithelial permeability and pulmonary edema^190^. - Activation of **SIRT1** by Resveratrol maintains the epithelial barrier by increasing the expression of TJ proteins **ZO1**, **Occludin** and **Claudin1**^191^. **SIRT1** negatively regulates **MMP9**, and reduction of **SIRT1** levels through oxidative stress confers an increase in **MMP9**^192^. - Activation of **FAS** increases barrier permeability and decreases the expression of **Occludin** and **ZO1** in the alveolar-capillary membrane and in alveolar epithelium^193^. - **FAS** is highly induced in response to **miR200c** overexpression^96^. At the same time **miR200c** represses **FAP1**, a negative regulator of **FAS**^194^. - **EZH2**-knockdown is accompanied by upregulation of **Occludin** and **Claudins**^195^. - **TRPV4** activation (which is  sensitized by **B1R**,^30^) leads to increased permeability of the alveolar septal barrier and pulmonary edema^149, 196^, while TRPV4 inhibition may reduce pulmonary edema in heart failure patients^197, 198^. **TRPV4** induces reversible epithelial cell permeability^199^, while **TRPV4** antagonism counteracts the diabetes-like effects on endothelial permeability^43^. In a model of cerebral artery occlusion **TRPV4** inhibition attenuated the loss of TJ proteins **Occludin** and **ZO-1^200^**. | | - **ACE2** downregulation, **B1R** activation and **miR200c** expression increase barrier permeability by impairing TJs and inducing enzymes degrading the extracellular matrix. - The effect **SIRT1** and **EZH2**, on barrier integrity are consistent with their regulatory roles in the ACE2-DAK-B1R axis. - Down-regulation of TJ proteins is observed in other **COVID-19** **associated pathologies** involving epithelial barrier defects such as in **kidney injury**^201^ or **Kawasaki disease,** where decreased ZO-1 levels are associated with intestinal barrier dysfunction^202^. - In **VILI** and inflammatory lung diseases predisposing patients to severe lung failures, **Occludin** and **ZO1** are reduced in alveolar epithelia cells^203^. Up-regulation of **Occludin** ameliorates **VILI**^68^  and **unfractionated heparin** attenuates ALI by upregulating **Claudin**, **ZO1** and **Occludin**^204^. - **COVID-19** patients with respiratory failure show increased **MMP9**^205^. **MMP9** degrades the base membrane. Levels of **MMP9** are elevated and predictive in ALI/ARDS^206, 207^. **MMP9** also impairs and actively degrades components of the BBB, leading to the development of **cerebral edema**^208, 209^. In **CKD**, **MMP9**^210^. - **Aging** exacerbates ALI-induced changes of the epithelial barrier, lung function, and inflammation. ALI in old mice is associated with high **MMP9** and significantly reduced **Occludin** levels^211^. This is consistent with the role of members of the ACE2-DAK-B1R signaling axis in ageing. - **TRPV4**, which is sensitized by **B1R** activity, may contribute to increased barrier permeability and edema. | - Exposure of human lung tissue to severe COVID-19 plasma caused loss of the junctional molecule occludin.^212^ |
| **Fibrogenic Signaling**  (https://covid19.molecularhealth.com/t/submodels/54.html) | | | |  |
| **COVID-19 Phenotype** | **Molecular Pathology** | | **Relation to Model and Findings** |  |
| **ARDS-associated fibrosis**  COVID-19 patients develop ARDS, that is characterized by rapid onset fibrosis^144^. | - **ACE2** is protective but downregulated in human lung **fibrosis**^213, 214^. - **ACE2** inhibits signaling pathways of tissue fibrosis in models of atherosclerosis, cerebral ischemia, obesity, chronic kidney disease, liver diseases and asthma^215^. **ACE2** deficiency increases cardiac mortality and adverse remodeling^216^. - Pharmacological activation of **ACE2** prevents lung fibrosis^217^, inhibits cardiac fibrosis and decreases cardiac collagen^218^. - **DAKD** engagement of **B1R** stimulates **Collagen** synthesis^219^ . Inhibition of **B1R** reduces renal fibrosis in obstructive nephropathy^220^ and ameliorates glomerulonephritis^221^ . Deletion of **B1R** reduces renal fibrosis^222^  and attenuates cardiac fibrosis^223^. - **Kallikrein** and **TMPRSS2**, via activation of **EGFR** contribute to fibrosis formation. **EGFR** signaling has been linked to increased fibrosis after **SARS-CoV** infection^224^ . In vascular smooth muscles, **Kallikrein** stimulates ADAM17 activity via a **PAR1/2** receptor-dependent mechanism, leading to the release of the endogenous ADAM17 substrates, amphiregulin and TNFα and MMP-dependent transactivation of **EGFR**^225^. **EGFR** contributes to the onset of pulmonary fibrosis^226^. Activation of **PAR2** by **Kallikrein** induces **EGFR** transactivation^225^  and in airways **PAR2** contributes to pulmonary fibrosis^227^. **PAR2** dependent transactivation of **EGFR** has been shown to contribute to renal fibrosis^228^. - **TMPRSS2** is an activator of **PAR2**^189^. - **miR200c** drives the development of fibrosis. It increases expression of **collagen** and **fibroblast growth factor**^229^. **miR200c** is aberrantly expressed in fibrosis of lung, liver, kidney, peritoneum and skin and serum levels are an early diagnostic biomarker of lung fibrosis^230^ . - Stressed cardiomyocytes release MVs highly enriched in **miR200c**, inducing the spread of fibrotic events^231, 232^. - In prostate cancer, **miR200c** induces expression of **EGR1**^96^. **SARS-CoV** induces **EGR1** dependent activation of **TGFβ** leading to a profibrotic response^233^. Targeting of **EGR1** ameliorates fibrosis in the lung^234^. - Overexpression of **miR200c** enhances **mortalin**, activity^235^. **Mortalin** deficiency suppresses fibrosis^236^ . - Hepatitis C Virus induced **miR200c** promotes fibrosis^229^. - **SIRT1** attenuates **TGFβ** mediated lung fibrosis^237^  and protects against pulmonary fibrosis^238^ **. TGFβ** downregulates **ACE2** expression in a **SIRT1**-dependent manner^239^. Pharmacological activation of **SIRT1** attenuates fibrosis^240^  and **Metformin**, an enhancer of **SIRT1** and **AMPK** expression^241^  reverses lung fibrosis. **AMPK** activity is reduced in fibrotic regions^242^ . - **TRPV4** is sensitized by **B1R^30^**. **TRPV4** plays a significant role in fibrosis of multiple organ systems including myocardial fibrosis, cystic fibrosis, pulmonary fibrosis, hepatic fibrosis and pancreatic fibrosis, reviewed in^87^. | | - **ACE2** downregulation, **B1R** activation and **miR200c** expression promote fibrosis. - The effects **Kallikrein**, **TMPRSS2**, **SIRT1 and** **AMPK**, on fibrogenic signaling are consistent with their respective roles in the context of the **ACE2**/**DAK**/**B1R** **axis**. - **TRPV4** which is sensitized by **B1R** activity, is mechanistically associated with fibrosis in multiple organs. |  |
| **Autoimmunity**  (https://covid19.molecularhealth.com/t/submodels/63.html) | | | |  |
| **COVID-19 Phenotype** | | **Molecular Pathology** | **Relation to Model and Findings** |  |
| **Autoimmune diseases (e.g., Kawasaki disease)**  COVID-19 precedes the appearance of various autoimmune diseases^243^. Autoimmune hemolytic anemia, immune thrombocytopenic purpura, Guillain-Barré syndrome have each been reported in patients with COVID-19^244^. Clinical manifestations resembling Kawasaki Disease n children is associated with a new phenotype of autoimmunity^245^. Antineutrophilic autoantibodies (ANCAs) have been identified in cases of COVID-19 associated vasculitis and glomerulonephritis^246^  and an increased risk of thrombotic events appears to be associated with the presence of antiphospholipid autoantibodies^247-250-292^. Disease severity and poor clinical outcomes are correlated with intense activation of extrafollicular B-cell responses^251^. The immunological landscape associated with this effector B cell mobilization in COVID-19 is similar to the one observed in patients with active autoimmune processes. Similar to systemic lupus erythematosus (SLE), antibodies generated through this process could be pathogenic, on the basis of ^252, 253^. | | - **B1R** is involved in the development of autoimmune diseases such as multiple sclerosis^254^, Crohn’s disease, ulcerative colitis, and inflammatory bowel disease^255, 256^. - **B1R** is upregulated on T-cells from peripheral blood of patients with multiple sclerosis^185, 257^. - Leukocyte **B1R** plays a critical role in the pathogenesis of **ANCA** glomerulonephritis^202^. - **B1R** blockade suppresses systemic autoimmunity in lupus nephritis^258^. - **B1R** is expressed on dendritic cells^259^. **B1R** blockade ameliorates systemic immunity by silencing dendritic cells and helper T cells. - Inhibition of **B1R** protects from autoimmune CNS disease^185^. - **miR200c** is elevated in primary immune thrombocytopenia^260^. - Autoimmunity is tightly connected to stem cell senescence and **immune-senescence**. As described above, the **ACE2**/**DAK**/**B1R** axis via dysregulation of **p16INK4a** might drive stem cell senescence. SLE patients show enhanced senescence of MSCs^261^, which is induced by upregulation of **p16INK4a**^262^. | - **B1R** activation and **miR200c** are associated with autoimmunity. - Immune-senescence induced via the **ACE2**/**DAK**/**B1R** axis might be involved in the autoimmunity phenotype. |  |

Page Break**Table 2:** Readouts of COVID-19 trials utilizing drugs that have the potential to increase ACE2 expression^263^.

| ***System/Drug group*** | ***Drug*** | ***Target*** | ***Indication (inclusion criteria)*** | ***Outcome*** | ***Reference*** *(PMID)* |
| --- | --- | --- | --- | --- | --- |
| **Kinin-Kallikrein** | Berinert  (C1 inhibitor) | C1R & C1S | Severe COVID-19 pneumonia:   SpO2 ≤ 94% in ambient air or Pa02/FiO2 ≤ 300 mmHg | - no change in "Time to clinical improvement" - no change to coagulation parameters - eosinophils increased | 3669276^264^ |
|  | Icatibant   (B2R   antagonist) | BDKRB2 | Severe COVID-19 pneumonia:   SpO2 ≤ 94% in ambient air or Pa02/FiO2 ≤ 300 mmHg | - no change in "Time to clinical improvement" - no change to coagulation parameters - eosinophils count increased | 3669276^264^ |
|  | Icatibant  (B2R  antagonist) | BDKRB2 | Severe COVID-19 pneumonia:  > 3 L/min supplemental oxygen,  a CT severity score of ≥7. | - 89% (8/9 patients) had reduction of >3 L/min oxygen supplementation 24 hours post treatment. - No severe adverse events - No clear association with D-dimer levels or fever | 32789513^265^ |
|  | Conestat Alfa   (C1 inhibitor) | C1R & C1S | Moderate or severe COVID-19 pneumonia by CT scan,   C-reactive protein level of at least 30 mg/L   oxygen saturation of <93% at rest in ambient air | - Immediate defervescence in 4 out of 5 patients - 2.5x reduction in intubation or death - No change in length of hospitalization | 32922409^266^ |
| **ACE Inhibitors & ARBs** | Various ACEi and ARBs | ACE & ATR1 | COVID-19 patients with ACEi or ARBs prescription before contracting COVID-19.  The study compared outcomes between patients randomly assigned to discontinue or continue ACEi or ARBs upon hospitalization | - no difference in outcomes between discontinued or continued use of ACEi or ARBs during COVID-19 hospitalization | 33422263^267^,  33464336^268^ |
| **TRP channels** | GSK2798745  (antagonist) | TRPV4 | Lung congestion in patients with heart failure:  Patients with Heart failure NYHA Class II/III | - improved lung diffusing capacity for carbon monoxide DLco (only trend not significant) - no serious adverse events | 32227554^269^,  30637626^270^ |
| **Vitamin D** | Calcifediol |  | Patients hospitalized with COVID-19 acute respiratory infection (Radiography confirmed) | - significant reduction of ICU admission (2% vs 50%) - limitation – comparison groups not fully matched - no information on BMI (and thus potential obesity) | 32871238^271^ |
| **Calcium release-activated Calcium (CRAC) channel** | Auxora (CM4620)  (inhibitor) | ORAI1 | Severe COVID-19 pneumonia (chest imaging) with respiratory compromise (e.g. ≥ 30 breaths/min, heart rate ≥ 125 bpm, SpO2 < 93% on room air or PaO2/FiO2 < 300) | - reduced median time to recovery (5 vs 12 days) - reduced risk of intubation (18% vs 50%) - combined death or intubation hazard ratio: 0.23 | 32795330^263^ |
| **Non-steroidal anti-inflammatory (NSAIDs)** | Acetylsalicylic acid |  | Patients hospitalized with COVID-19 pneumonia | - significantly reduced in-hospital death rate | 33476420^272^ |
|  | Acetylsalicylic acid + Anti-coagulation (tirofiban, clopidogrel, fondaparinux) |  | Patients with COIVD19 severe respiratory failure requiring Continuous positive airway pressure (CPAP) | - reduction in A-a O_2_ gradient - increased  PaO2/FiO2 ratio - earlier weaning from CPAP | 32450344^273^ |
| **Glucose lowering drugs** | Metformin | PRKAB1, GPD1, ETFDH | Study of susceptibility to contract COVID-19 in diabetic patients using Metformin | - no significant association of metformin and risk of COVID-19 | 33560344^274^ |
|  | Metformin | PRKAB1, GPD1, ETFDH | Hospitalized COVID-19 patients using Metformin prior to COVID-19 infection | - reduced mortality/risk of death - reduced risk of acute ischemic stroke - some studies point to higher positive effect in females - some studies show initially more severe course of disease in diabetic patients but overall lower mortality | 33745895^275^, 33662839^276^, 33580540^277^, 33519709^278^, 33309936^279^, 33232684^280^, 33471718^281^, Meta-analyses: 32844132^282^, 33395778^283^ |
|  | Gliptin  (DPP4 inhibitor) | DPP4 | Meta-analysis of 9 studies with COVID-19 patients using DPP-4 inhibitor | - reduced risk ration (RR) of death (0.76) | 33838614^284^ |

1. Hampshire, A. et al. Cognitive deficits in people who have recovered from COVID-19 relative to controls: An N=84,285 online study. *medRxiv*, 2020.2010.2020.20215863 (2020).

2. Baker, H.A., Safavynia, S.A. & Evered, L.A. The 'third wave': impending cognitive and functional decline in COVID-19 survivors. *Br J Anaesth* (2020).

3. Ramos-Miguel, A. et al. Frontotemporal dysregulation of the SNARE protein interactome is associated with faster cognitive decline in old age. *Neurobiol Dis* **114**, 31-44 (2018).

4. Honer, W.G. et al. Cognitive reserve, presynaptic proteins and dementia in the elderly. *Transl Psychiatry* **2**, e114 (2012).

5. Boyle, P.A. et al. Much of late life cognitive decline is not due to common neurodegenerative pathologies. *Ann Neurol* **74**, 478-489 (2013).

6. Menni, C. et al. Real-time tracking of self-reported symptoms to predict potential COVID-19. *Nat Med* **26**, 1037-1040 (2020).

7. Eliezer, M. et al. Sudden and Complete Olfactory Loss of Function as a Possible Symptom of COVID-19. *JAMA Otolaryngol Head Neck Surg* **146**, 674-675 (2020).

8. Gautier, J.F. & Ravussin, Y. A New Symptom of COVID-19: Loss of Taste and Smell. *Obesity (Silver Spring)* **28**, 848 (2020).

9. Pellegrino, R. et al. Corona Viruses and the Chemical Senses: Past, Present, and Future. *Chem Senses* (2020).

10. Marcucci, F., Zou, D.J. & Firestein, S. Sequential onset of presynaptic molecules during olfactory sensory neuron maturation. *J Comp Neurol* **516**, 187-198 (2009).

11. Yang, R., Ma, H., Thomas, S.M. & Kinnamon, J.C. Immunocytochemical analysis of syntaxin-1 in rat circumvallate taste buds. *J Comp Neurol* **502**, 883-893 (2007).

12. Xu, H. et al. High expression of ACE2 receptor of 2019-nCoV on the epithelial cells of oral mucosa. *Int J Oral Sci* **12**, 8 (2020).

13. Sungnak, W., Huang, N., Bécavin, C. & Berg, M. SARS-CoV-2 Entry Genes Are Most Highly Expressed in Nasal Goblet and Ciliated Cells within Human Airways. *ArXiv* (2020).

14. Shigemura, N. et al. Expression of Renin-Angiotensin System Components in the Taste Organ of Mice. *Nutrients* **11** (2019).

15. Wilkerson, R.G., Adler, J.D., Shah, N.G. & Brown, R. Silent hypoxia: A harbinger of clinical deterioration in patients with COVID-19. *Am J Emerg Med* **38**, 2243.e2245-2243.e2246 (2020).

16. López-Barneo, J., Ortega-Sáenz, P., Pardal, R., Pascual, A. & Piruat, J.I. Carotid body oxygen sensing. *Eur Respir J* **32**, 1386-1398 (2008).

17. Koerner, P., Hesslinger, C., Schaefermeyer, A., Prinz, C. & Gratzl, M. Evidence for histamine as a transmitter in rat carotid body sensor cells. *J Neurochem* **91**, 493-500 (2004).

18. Porzionato, A. et al. Case Report: The Carotid Body in COVID-19: Histopathological and Virological Analyses of an Autopsy Case Series. *Front Immunol* **12**, 736529 (2021).

19. Chee, Y.J., Ng, S.J.H. & Yeoh, E. Diabetic ketoacidosis precipitated by Covid-19 in a patient with newly diagnosed diabetes mellitus. *Diabetes Res Clin Pract* **164**, 108166 (2020).

20. Li, J. et al. COVID-19 infection may cause ketosis and ketoacidosis. *Diabetes Obes Metab* **22**, 1935-1941 (2020).

21. Ren, H. et al. Association of the insulin resistance marker TyG index with the severity and mortality of COVID-19. *Cardiovasc Diabetol* **19**, 58 (2020).

22. Zhang, Q. et al. Serum proteomics reveals systemic dysregulation of innate immunity in type 1 diabetes. *J Exp Med* **210**, 191-203 (2013).

23. Gaisano, H.Y. Deploying insulin granule-granule fusion to rescue deficient insulin secretion in diabetes. *Diabetologia* **55**, 877-880 (2012).

24. Südhof, T.C. & Rothman, J.E. Membrane fusion: grappling with SNARE and SM proteins. *Science (New York, N.Y.)* **323**, 474-477 (2009).

25. Niu, M.J., Yang, J.K., Lin, S.S., Ji, X.J. & Guo, L.M. Loss of angiotensin-converting enzyme 2 leads to impaired glucose homeostasis in mice. *Endocrine* **34**, 56-61 (2008).

26. El Akoum, S., Haddad, Y. & Couture, R. Impact of pioglitazone and bradykinin type 1 receptor antagonist on type 2 diabetes in high-fat diet-fed C57BL/6J mice. *Obes Sci Pract* **3**, 352-362 (2017).

27. Haddad, Y. & Couture, R. Kininase 1 As a Preclinical Therapeutic Target for Kinin B(1) Receptor in Insulin Resistance. *Frontiers in pharmacology* **8**, 509 (2017).

28. Catanzaro, O.L., Dziubecki, D., Obregon, P., Rodriguez, R.R. & Sirois, P. Antidiabetic efficacy of bradykinin antagonist R-954 on glucose tolerance test in diabetic type 1 mice. *Neuropeptides* **44**, 187-189 (2010).

29. Seguin, T. et al. Hemodynamic and renal involvement of B1 and B2 kinin receptors during the acute phase of endotoxin shock in mice. *Int Immunopharmacol* **8**, 217-221 (2008).

30. Costa, R. et al. Kinin Receptors Sensitize TRPV4 Channel and Induce Mechanical Hyperalgesia: Relevance to Paclitaxel-Induced Peripheral Neuropathy in Mice. *Mol Neurobiol* **55**, 2150-2161 (2018).

31. Vellani, V., Zachrisson, O. & McNaughton, P.A. Functional bradykinin B1 receptors are expressed in nociceptive neurones and are upregulated by the neurotrophin GDNF. *J Physiol* **560**, 391-401 (2004).

32. Gassaway, B.M. et al. PKCε contributes to lipid-induced insulin resistance through cross talk with p70S6K and through previously unknown regulators of insulin signaling. *Proc Natl Acad Sci U S A* **115**, E8996-e9005 (2018).

33. Sales, V.M. et al. Kinin B(1) Receptor Acts in Adipose Tissue to Control Fat Distribution in a Cell-Nonautonomous Manner. *Diabetes* **68**, 1614-1623 (2019).

34. Masotti, A. et al. Oral glucose tolerance test unravels circulating miRNAs associated with insulin resistance in obese preschoolers. *Pediatr Obes* **12**, 229-238 (2017).

35. Belgardt, B.F. et al. The microRNA-200 family regulates pancreatic beta cell survival in type 2 diabetes. *Nat Med* **21**, 619-627 (2015).

36. Zhang, H. et al. Inhibition of miR-200c Restores Endothelial Function in Diabetic Mice Through Suppression of COX-2. *Diabetes* **65**, 1196-1207 (2016).

37. Su, W. et al. MiR200c targets IRS1 and suppresses prostate cancer cell growth. *Prostate* **75**, 855-862 (2015).

38. Xi, G., Wai, C., White, M.F. & Clemmons, D.R. Down-regulation of Insulin Receptor Substrate 1 during Hyperglycemia Induces Vascular Smooth Muscle Cell Dedifferentiation. *J Biol Chem* **292**, 2009-2020 (2017).

39. Wu, M.J. et al. Epithelial-Mesenchymal Transition Directs Stem Cell Polarity via Regulation of Mitofusin. *Cell Metab* **29**, 993-1002.e1006 (2019).

40. Besse-Patin, A. et al. PGC1A regulates the IRS1:IRS2 ratio during fasting to influence hepatic metabolism downstream of insulin. *Proc Natl Acad Sci U S A* **116**, 4285-4290 (2019).

41. Ye, L. et al. TRPV4 is a regulator of adipose oxidative metabolism, inflammation, and energy homeostasis. *Cell* **151**, 96-110 (2012).

42. Hills, C.E., Bland, R. & Squires, P.E. Functional expression of TRPV4 channels in human collecting duct cells: implications for secondary hypertension in diabetic nephropathy. *Exp Diabetes Res* **2012**, 936518 (2012).

43. Arredondo Zamarripa, D. et al. Dual contribution of TRPV4 antagonism in the regulatory effect of vasoinhibins on blood-retinal barrier permeability: diabetic milieu makes a difference. *Sci Rep* **7**, 13094 (2017).

44. Dias, F.C. et al. The selective TRPV4 channel antagonist HC-067047 attenuates mechanical allodynia in diabetic mice. *Eur J Pharmacol* **856**, 172408 (2019).

45. Ofori, J.K. et al. Human Islet MicroRNA-200c Is Elevated in Type 2 Diabetes and Targets the Transcription Factor ETV5 to Reduce Insulin Secretion. *Diabetes* **71**, 275-284 (2022).

46. Pimenta, R. et al. MiR-200c-3p expression may be associated with worsening of the clinical course of patients with COVID-19. *Mol Biol Res Commun* **10**, 141-147 (2021).

47. Martínez-Alemán, S.R. et al. Understanding the Entanglement: Neutrophil Extracellular Traps (NETs) in Cystic Fibrosis. *Front Cell Infect Microbiol* **7**, 104 (2017).

48. Cutting, G.R. Cystic fibrosis genetics: from molecular understanding to clinical application. *Nat Rev Genet* **16**, 45-56 (2015).

49. Jena, B.P. Porosome in Cystic Fibrosis. *Discoveries (Craiova)* **2** (2014).

50. Jena, B.P. Porosome: the secretory portal in cells. *Biochemistry* **48**, 4009-4018 (2009).

51. Lee, S. et al. Virus-induced senescence is a driver and therapeutic target in COVID-19. *Nature* **599**, 283-289 (2021).

52. Tsuji, S. et al. SARS-CoV-2 infection triggers paracrine senescence and leads to a sustained senescence-associated inflammatory response. *Nature Aging* **2**, 115-124 (2022).

53. Baj, J. et al. COVID-19: Specific and Non-Specific Clinical Manifestations and Symptoms: The Current State of Knowledge. *J Clin Med* **9** (2020).

54. Hamming, I. et al. Tissue distribution of ACE2 protein, the functional receptor for SARS coronavirus. A first step in understanding SARS pathogenesis. *J Pathol* **203**, 631-637 (2004).

55. Takeshita, H. et al. Different effects of the deletion of angiotensin converting enzyme 2 and chronic activation of the renin-angiotensin system on muscle weakness in middle-aged mice. *Hypertens Res* **43**, 296-304 (2020).

56. Takeshita, H. et al. Angiotensin-converting enzyme 2 deficiency accelerates and angiotensin 1-7 restores age-related muscle weakness in mice. *J Cachexia Sarcopenia Muscle* **9**, 975-986 (2018).

57. Sousa-Victor, P. et al. Geriatric muscle stem cells switch reversible quiescence into senescence. *Nature* **506**, 316-321 (2014).

58. Zhu, P. et al. The transcription factor Slug represses p16(Ink4a) and regulates murine muscle stem cell aging. *Nat Commun* **10**, 2568 (2019).

59. Nozato, S. et al. Angiotensin 1-7 alleviates aging-associated muscle weakness and bone loss, but is not associated with accelerated aging in ACE2-knockout mice. *Clin Sci (Lond)* **133**, 2005-2018 (2019).

60. Liu, J.Y. et al. Cells exhibiting strong p16 (INK4a) promoter activation in vivo display features of senescence. *Proc Natl Acad Sci U S A* **116**, 2603-2611 (2019).

61. Jacobs, J.J., Kieboom, K., Marino, S., DePinho, R.A. & van Lohuizen, M. The oncogene and Polycomb-group gene bmi-1 regulates cell proliferation and senescence through the ink4a locus. *Nature* **397**, 164-168 (1999).

62. Cao, Q. et al. Coordinated regulation of polycomb group complexes through microRNAs in cancer. *Cancer Cell* **20**, 187-199 (2011).

63. Liu, L. et al. miR-200c inhibits invasion, migration and proliferation of bladder cancer cells through down-regulation of BMI-1 and E2F3. *J Transl Med* **12**, 305 (2014).

64. De Biasi, S. et al. Marked T cell activation, senescence, exhaustion and skewing towards TH17 in patients with COVID-19 pneumonia. *Nat Commun* **11**, 3434 (2020).

65. Iwama, A. et al. Enhanced self-renewal of hematopoietic stem cells mediated by the polycomb gene product Bmi-1. *Immunity* **21**, 843-851 (2004).

66. Rizo, A., Dontje, B., Vellenga, E., de Haan, G. & Schuringa, J.J. Long-term maintenance of human hematopoietic stem/progenitor cells by expression of BMI1. *Blood* **111**, 2621-2630 (2008).

67. Di Carlo, V., Mocavini, I. & Di Croce, L. Polycomb complexes in normal and malignant hematopoiesis. *The Journal of cell biology* **218**, 55-69 (2019).

68. Liu, M., Gu, C. & Wang, Y. Upregulation of the tight junction protein occludin: effects on ventilation-induced lung injury and mechanisms of action. *BMC Pulm Med* **14**, 94 (2014).

69. Nehme, J., Borghesan, M., Mackedenski, S., Bird, T.G. & Demaria, M. Cellular senescence as a potential mediator of COVID-19 severity in the elderly. *Aging Cell* **19**, e13237 (2020).

70. Samsonraj, R.M. et al. Concise Review: Multifaceted Characterization of Human Mesenchymal Stem Cells for Use in Regenerative Medicine. *Stem Cells Transl Med* **6**, 2173-2185 (2017).

71. Li, N. & Hua, J. Interactions between mesenchymal stem cells and the immune system. *Cell Mol Life Sci* **74**, 2345-2360 (2017).

72. Leng, Z. et al. Transplantation of ACE2(-) Mesenchymal Stem Cells Improves the Outcome of Patients with COVID-19 Pneumonia. *Aging Dis* **11**, 216-228 (2020).

73. Jung, Y. & Nolta, J.A. BMI1 Regulation of Self-Renewal and Multipotency in Human Mesenchymal Stem Cells. *Curr Stem Cell Res Ther* **11**, 131-140 (2016).

74. Lee, J.Y. et al. BMI1 inhibits senescence and enhances the immunomodulatory properties of human mesenchymal stem cells via the direct suppression of MKP-1/DUSP1. *Aging (Albany NY)* **8**, 1670-1689 (2016).

75. Dai, J., Zhu, X., Yoder, M.C., Wu, Y. & Colman, R.W. Cleaved high-molecular-weight kininogen accelerates the onset of endothelial progenitor cell senescence by induction of reactive oxygen species. *Arterioscler Thromb Vasc Biol* **31**, 883-889 (2011).

76. Liu, F., Gao, F., Li, Q. & Liu, Z. The functional study of human umbilical cord mesenchymal stem cells harbouring angiotensin-converting enzyme 2 in rat acute lung ischemia-reperfusion injury model. *Cell Biochem Funct* **32**, 580-589 (2014).

77. Chen, J. et al. Angiotensin-converting enzyme 2 priming enhances the function of endothelial progenitor cells and their therapeutic efficacy. *Hypertension* **61**, 681-689 (2013).

78. Qi, Y. et al. Diminazene aceturate enhances angiotensin-converting enzyme 2 activity and attenuates ischemia-induced cardiac pathophysiology. *Hypertension* **62**, 746-752 (2013).

79. Lambert, N.J.S.C. COVID-19 “Long Hauler” Symptoms Survey Report. *Indiana University School of*

*Medicine (Baltimore)* (2020).

80. Miyazato, Y. et al. Prolonged and Late-Onset Symptoms of Coronavirus Disease 2019. *Open Forum Infect Dis* **7**, ofaa507 (2020).

81. Driskell, R.R., Clavel, C., Rendl, M. & Watt, F.M. Hair follicle dermal papilla cells at a glance. *Journal of cell science* **124**, 1179-1182 (2011).

82. Bahta, A.W., Farjo, N., Farjo, B. & Philpott, M.P. Premature senescence of balding dermal papilla cells in vitro is associated with p16(INK4a) expression. *J Invest Dermatol* **128**, 1088-1094 (2008).

83. Mehta, P. et al. COVID-19: consider cytokine storm syndromes and immunosuppression. *Lancet* **395**, 1033-1034 (2020).

84. Tao, L. et al. Angiotensin-converting enzyme 2 activator diminazene aceturate prevents lipopolysaccharide-induced inflammation by inhibiting MAPK and NF-κB pathways in human retinal pigment epithelium. *J Neuroinflammation* **13**, 35 (2016).

85. Zhang, Q. et al. Novel pharmacological inhibition of EZH2 attenuates septic shock by altering innate inflammatory responses to sepsis. *Int Immunopharmacol* **76**, 105899 (2019).

86. Xue, T., Wei, N., Xin, Z. & Qingyu, X. Angiotensin-converting enzyme-2 overexpression attenuates inflammation in rat model of chronic obstructive pulmonary disease. *Inhal Toxicol* **26**, 14-22 (2014).

87. Zhang, J. et al. AMP-activated Protein Kinase Phosphorylation of Angiotensin-Converting Enzyme 2 in Endothelium Mitigates Pulmonary Hypertension. *Am J Respir Crit Care Med* **198**, 509-520 (2018).

88. Li, G. et al. Angiotensin-converting enzyme 2 activation protects against pulmonary arterial hypertension through improving early endothelial function and mediating cytokines levels. *Chin Med J (Engl)* **125**, 1381-1388 (2012).

89. Haga, S. et al. A novel ACE2 activator reduces monocrotaline-induced pulmonary hypertension by suppressing the JAK/STAT and TGF-β cascades with restored caveolin-1 expression. *Exp Lung Res* **41**, 21-31 (2015).

90. Sodhi, C.P. et al. Attenuation of pulmonary ACE2 activity impairs inactivation of des-Arg(9) bradykinin/BKB1R axis and facilitates LPS-induced neutrophil infiltration. *Am J Physiol Lung Cell Mol Physiol* **314**, L17-l31 (2018).

91. McLean, P.G., Perretti, M. & Ahluwalia, A. Kinin B(1) receptors and the cardiovascular system: regulation of expression and function. *Cardiovasc Res* **48**, 194-210 (2000).

92. Ahluwalia, A. & Perretti, M. Involvement of bradykinin B1 receptors in the polymorphonuclear leukocyte accumulation induced by IL-1 beta in vivo in the mouse. *J Immunol* **156**, 269-274 (1996).

93. Ehrenfeld, P. et al. Kinin B1 receptor activation turns on exocytosis of matrix metalloprotease-9 and myeloperoxidase in human neutrophils: involvement of mitogen-activated protein kinase family. *Journal of leukocyte biology* **86**, 1179-1189 (2009).

94. Kuhr, F., Lowry, J., Zhang, Y., Brovkovych, V. & Skidgel, R.A. Differential regulation of inducible and endothelial nitric oxide synthase by kinin B1 and B2 receptors. *Neuropeptides* **44**, 145-154 (2010).

95. Othman, R., Vaucher, E. & Couture, R. Bradykinin Type 1 Receptor - Inducible Nitric Oxide Synthase: A New Axis Implicated in Diabetic Retinopathy. *Frontiers in pharmacology* **10**, 300 (2019).

96. Tryndyak, V.P., Beland, F.A. & Pogribny, I.P. E-cadherin transcriptional down-regulation by epigenetic and microRNA-200 family alterations is related to mesenchymal and drug-resistant phenotypes in human breast cancer cells. *Int J Cancer* **126**, 2575-2583 (2010).

97. Tao, J. et al. miR-200c Modulates the Pathogenesis of Radiation-Induced Oral Mucositis. *Oxid Med Cell Longev* **2019**, 2352079 (2019).

98. Laforge, M. et al. Tissue damage from neutrophil-induced oxidative stress in COVID-19. *Nat Rev Immunol* **20**, 515-516 (2020).

99. Wu, Y.H. et al. A novel fine tuning scheme of miR-200c in modulating lung cell redox homeostasis. *Free Radic Res* **51**, 591-603 (2017).

100. Liu, Q. et al. miRNA-200c-3p is crucial in acute respiratory distress syndrome. *Cell Discovery* **3**, 17021 (2017).

101. Carlomosti, F. et al. Oxidative Stress-Induced miR-200c Disrupts the Regulatory Loop Among SIRT1, FOXO1, and eNOS. *Antioxid Redox Signal* **27**, 328-344 (2017).

102. Yan, M. et al. Regulation of iNOS-Derived ROS Generation by HSP90 and Cav-1 in Porcine Reproductive and Respiratory Syndrome Virus-Infected Swine Lung Injury. *Inflammation* **40**, 1236-1244 (2017).

103. Kellner, M. et al. ROS Signaling in the Pathogenesis of Acute Lung Injury (ALI) and Acute Respiratory Distress Syndrome (ARDS). *Adv Exp Med Biol* **967**, 105-137 (2017).

104. Janssen, W.J. & Nozik-Grayck, E. Power of Place: Intravascular Superoxide Dismutase for Prevention of Acute Respiratory Distress Syndrome. *Am J Respir Cell Mol Biol* **56**, 147-149 (2017).

105. Chen, X. et al. Heme Oxygenase-1 Reduces Sepsis-Induced Endoplasmic Reticulum Stress and Acute Lung Injury. *Mediators Inflamm* **2018**, 9413876 (2018).

106. De Virgiliis, F. & Di Giovanni, S. Lung innervation in the eye of a cytokine storm: neuroimmune interactions and COVID-19. *Nat Rev Neurol* **16**, 645-652 (2020).

107. Cernit, V., Sénécal, J., Othman, R. & Couture, R. Reciprocal Regulatory Interaction between TRPV1 and Kinin B1 Receptor in a Rat Neuropathic Pain Model. *International journal of molecular sciences* **21** (2020).

108. Xu, X. et al. Increases in transient receptor potential vanilloid-1 mRNA and protein in primary afferent neurons stimulated by protein kinase C and their possible role in neurogenic inflammation. *J Neurosci Res* **87**, 482-494 (2009).

109. Dib, M. et al. A paradoxical protective role for the proinflammatory peptide substance P receptor (NK1R) in acute hyperoxic lung injury. *Am J Physiol Lung Cell Mol Physiol* **297**, L687-697 (2009).

110. Nahama, A., Ramachandran, R., Cisternas, A.F. & Ji, H. The role of afferent pulmonary innervation in ARDS associated with COVID-19 and potential use of resiniferatoxin to improve prognosis: A review. *Med Drug Discov* **5**, 100033 (2020).

111. Omar, S. et al. Respiratory virus infection up-regulates TRPV1, TRPA1 and ASICS3 receptors on airway cells. *PLoS One* **12**, e0171681 (2017).

112. Baral, P. et al. Nociceptor sensory neurons suppress neutrophil and γδ T cell responses in bacterial lung infections and lethal pneumonia. *Nat Med* **24**, 417-426 (2018).

113. Edvinsson, L., Haanes, K.A. & Warfvinge, K. Does inflammation have a role in migraine? *Nat Rev Neurol* **15**, 483-490 (2019).

114. Toptan, T., Aktan, Ç., Başarı, A. & Bolay, H. Case Series of Headache Characteristics in COVID-19: Headache Can Be an Isolated Symptom. *Headache* **60**, 1788-1792 (2020).

115. Struyf, T. et al. Signs and symptoms to determine if a patient presenting in primary care or hospital outpatient settings has COVID-19 disease. *Cochrane Database Syst Rev* **7**, Cd013665 (2020).

116. Hamza, M. et al. Kinin B1 receptors contributes to acute pain following minor surgery in humans. *Mol Pain* **6**, 12 (2010).

117. Talbot, S., De Brito Gariépy, H., Saint-Denis, J. & Couture, R. Activation of kinin B1 receptor evokes hyperthermia through a vagal sensory mechanism in the rat. *J Neuroinflammation* **9**, 214 (2012).

118. Lee, L.Y., Ni, D., Hayes, D., Jr. & Lin, R.L. TRPV1 as a cough sensor and its temperature-sensitive properties. *Pulm Pharmacol Ther* **24**, 280-285 (2011).

119. Adcock, J.J. TRPV1 receptors in sensitisation of cough and pain reflexes. *Pulm Pharmacol Ther* **22**, 65-70 (2009).

120. Meotti, F.C. et al. Inflammatory muscle pain is dependent on the activation of kinin B₁ and B₂ receptors and intracellular kinase pathways. *Br J Pharmacol* **166**, 1127-1139 (2012).

121. Mense, S. Muscle pain: mechanisms and clinical significance. *Dtsch Arztebl Int* **105**, 214-219 (2008).

122. Gabra, B.H. & Sirois, P. Pathways for the bradykinin B1 receptor-mediated diabetic hyperalgesia in mice. *Inflamm Res* **53**, 653-657 (2004).

123. Porreca, F. et al. Antinociceptive pharmacology of N-[[4-(4,5-dihydro-1H-imidazol-2-yl)phenyl]methyl]-2-[2-[[(4-methoxy-2,6-dimethylphenyl) sulfonyl]methylamino]ethoxy]-N-methylacetamide, fumarate (LF22-0542), a novel nonpeptidic bradykinin B1 receptor antagonist. *J Pharmacol Exp Ther* **318**, 195-205 (2006).

124. Arminoff, M.J.J., S. A:; Aminoff's Neurology and General Medicine. (2014).

125. Ferreira, J. et al. The role of kinin B1 receptors in the nociception produced by peripheral protein kinase C activation in mice. *Neuropharmacology* **54**, 597-604 (2008).

126. Chakrabarti, S. et al. Acute inflammation sensitizes knee-innervating sensory neurons and decreases mouse digging behavior in a TRPV1-dependent manner. *Neuropharmacology* **143**, 49-62 (2018).

127. Lee, M.G., Macglashan, D.W., Jr. & Undem, B.J. Role of chloride channels in bradykinin-induced guinea pig airway vagal C-fibre activation. *J Physiol* **566**, 205-212 (2005).

128. Rong, W. et al. Jejunal afferent nerve sensitivity in wild-type and TRPV1 knockout mice. *J Physiol* **560**, 867-881 (2004).

129. Pan, H.L. & Chen, S.R. Sensing tissue ischemia: another new function for capsaicin receptors? *Circulation* **110**, 1826-1831 (2004).

130. Carr, M.J., Kollarik, M., Meeker, S.N. & Undem, B.J. A role for TRPV1 in bradykinin-induced excitation of vagal airway afferent nerve terminals. *J Pharmacol Exp Ther* **304**, 1275-1279 (2003).

131. Talbot, S., Chahmi, E., Dias, J.P. & Couture, R. Key role for spinal dorsal horn microglial kinin B1 receptor in early diabetic pain neuropathy. *J Neuroinflammation* **7**, 36 (2010).

132. Cheah, F.Y., Baltic, S., Temple, S.E., Bhoola, K. & Thompson, P.J. Novel kinin B₁ receptor splice variant and 5'UTR regulatory elements are responsible for cell specific B₁ receptor expression. *PLoS One* **9**, e87175 (2014).

133. Aziz, M. et al. Taste Changes (Dysgeusia) in COVID-19: A Systematic Review and Meta-analysis. *Gastroenterology* **159**, 1132-1133 (2020).

134. Wotherspoon, G. & Winter, J. Bradykinin B1 receptor is constitutively expressed in the rat sensory nervous system. *Neurosci Lett* **294**, 175-178 (2000).

135. Ma, Q.P., Hill, R. & Sirinathsinghji, D. Basal expression of bradykinin B1 receptor in peripheral sensory ganglia in the rat. *Neuroreport* **11**, 4003-4005 (2000).

136. Roper, S.D. & Chaudhari, N. Taste buds: cells, signals and synapses. *Nat Rev Neurosci* **18**, 485-497 (2017).

137. Huang, A.Y. & Wu, S.Y. Calcitonin Gene-Related Peptide Reduces Taste-Evoked ATP Secretion from Mouse Taste Buds. *J Neurosci* **35**, 12714-12724 (2015).

138. Riera, C.E., Vogel, H., Simon, S.A. & le Coutre, J. Artificial sweeteners and salts producing a metallic taste sensation activate TRPV1 receptors. *Am J Physiol Regul Integr Comp Physiol* **293**, R626-634 (2007).

139. Jang, Y. et al. Olfactory and taste disorder: The first and only sign in a patient with SARS-CoV-2 pneumonia. *Infect Control Hosp Epidemiol* **41**, 1103 (2020).

140. Bhatraju, P.K. et al. Covid-19 in Critically Ill Patients in the Seattle Region - Case Series. *N Engl J Med* **382**, 2012-2022 (2020).

141. Arentz, M. et al. Characteristics and Outcomes of 21 Critically Ill Patients With COVID-19 in Washington State. *Jama* **323**, 1612-1614 (2020).

142. Richardson, S. et al. Presenting Characteristics, Comorbidities, and Outcomes Among 5700 Patients Hospitalized With COVID-19 in the New York City Area. *Jama* **323**, 2052-2059 (2020).

143. Wu, C. et al. Risk Factors Associated With Acute Respiratory Distress Syndrome and Death in Patients With Coronavirus Disease 2019 Pneumonia in Wuhan, China. *JAMA Intern Med* **180**, 934-943 (2020).

144. Zhou, F. et al. Clinical course and risk factors for mortality of adult inpatients with COVID-19 in Wuhan, China: a retrospective cohort study. *Lancet* **395**, 1054-1062 (2020).

145. Fan, E. et al. COVID-19-associated acute respiratory distress syndrome: is a different approach to management warranted? *Lancet Respir Med* **8**, 816-821 (2020).

146. Han, B., Lodyga, M. & Liu, M. Ventilator-induced lung injury: role of protein-protein interaction in mechanosensation. *Proc Am Thorac Soc* **2**, 181-187 (2005).

147. Matsumoto, K. et al. Transient receptor potential vanilloid 4 mediates sour taste sensing via type III taste cell differentiation. *Sci Rep* **9**, 6686 (2019).

148. Nayak, P.S. et al. Mechanotransduction via TRPV4 regulates inflammation and differentiation in fetal mouse distal lung epithelial cells. *Respir Res* **16**, 60 (2015).

149. Alvarez, D.F. et al. Transient receptor potential vanilloid 4-mediated disruption of the alveolar septal barrier: a novel mechanism of acute lung injury. *Circ Res* **99**, 988-995 (2006).

150. Hamanaka, K. et al. TRPV4 channels augment macrophage activation and ventilator-induced lung injury. *Am J Physiol Lung Cell Mol Physiol* **299**, L353-362 (2010).

151. White, J.P. et al. TRPV4: Molecular Conductor of a Diverse Orchestra. *Physiol Rev* **96**, 911-973 (2016).

152. Michalick, L. et al. In vitro screening identifies TRPV4 as target for endothelial barrier stabilization in COVID-19. *The FASEB Journal* **35** (2021).

153. Connors, J.M. & Levy, J.H. COVID-19 and its implications for thrombosis and anticoagulation. *Blood* **135**, 2033-2040 (2020).

154. Tang, N., Li, D., Wang, X. & Sun, Z. Abnormal coagulation parameters are associated with poor prognosis in patients with novel coronavirus pneumonia. *J Thromb Haemost* **18**, 844-847 (2020).

155. Tidjane, N. et al. A primary role for kinin B1 receptor in inflammation, organ damage, and lethal thrombosis in a rat model of septic shock in diabetes. *Eur J Inflamm* **13**, 40-52 (2015).

156. Qadri, F. & Bader, M. Kinin B1 receptors as a therapeutic target for inflammation. *Expert Opin Ther Targets* **22**, 31-44 (2018).

157. Varga-Szabo, D., Braun, A. & Nieswandt, B. Calcium signaling in platelets. *J Thromb Haemost* **7**, 1057-1066 (2009).

158. Langhauser, F. et al. Kininogen deficiency protects from ischemic neurodegeneration in mice by reducing thrombosis, blood-brain barrier damage, and inflammation. *Blood* **120**, 4082-4092 (2012).

159. Merkulov, S. et al. Deletion of murine kininogen gene 1 (mKng1) causes loss of plasma kininogen and delays thrombosis. *Blood* **111**, 1274-1281 (2008).

160. Saldeen, T., Ryan, J.W. & Berryer, P. A peptide derived from fibrin(ogen) inhibits angiotensin converting enzyme and potentiates the effects of bradykinin. *Thromb Res* **23**, 465-470 (1981).

161. Fraga-Silva, R.A. et al. ACE2 activation promotes antithrombotic activity. *Mol Med* **16**, 210-215 (2010).

162. Xiao, H.L. et al. Association between ACE2/ACE balance and pneumocyte apoptosis in a porcine model of acute pulmonary thromboembolism with cardiac arrest. *Mol Med Rep* **17**, 4221-4228 (2018).

163. Aulak, K.S., Davis, A.E., 3rd, Donaldson, V.H. & Harrison, R.A. Chymotrypsin inhibitory activity of normal C1-inhibitor and a P1 Arg to His mutant: evidence for the presence of overlapping reactive centers. *Protein Sci* **2**, 727-732 (1993).

164. Arcanjo, A. et al. The emerging role of neutrophil extracellular traps in severe acute respiratory syndrome coronavirus 2 (COVID-19). *Sci Rep* **10**, 19630 (2020).

165. Papayannopoulos, V. Neutrophil extracellular traps in immunity and disease. *Nat Rev Immunol* **18**, 134-147 (2018).

166. Saffarzadeh, M. et al. Neutrophil extracellular traps directly induce epithelial and endothelial cell death: a predominant role of histones. *PLoS One* **7**, e32366 (2012).

167. Porto, B.N. & Stein, R.T. Neutrophil Extracellular Traps in Pulmonary Diseases: Too Much of a Good Thing? *Front Immunol* **7**, 311 (2016).

168. Narasaraju, T. et al. Excessive neutrophils and neutrophil extracellular traps contribute to acute lung injury of influenza pneumonitis. *Am J Pathol* **179**, 199-210 (2011).

169. Hair, P.S., Enos, A.I., Krishna, N.K. & Cunnion, K.M. Inhibition of Immune Complex Complement Activation and Neutrophil Extracellular Trap Formation by Peptide Inhibitor of Complement C1. *Front Immunol* **9**, 558 (2018).

170. Wygrecka, M. et al. Antihistone Properties of C1 Esterase Inhibitor Protect against Lung Injury. *Am J Respir Crit Care Med* **196**, 186-199 (2017).

171. Ware, L.B. Physiological and biological heterogeneity in COVID-19-associated acute respiratory distress syndrome. *Lancet Respir Med* **8**, 1163-1165 (2020).

172. Wittekindt, O.H. Tight junctions in pulmonary epithelia during lung inflammation. *Pflugers Arch* **469**, 135-147 (2017).

173. Mugisho, O.O., Robilliard, L.D., Nicholson, L.F.B., Graham, E.S. & O'Carroll, S.J. Bradykinin receptor-1 activation induces inflammation and increases the permeability of human brain microvascular endothelial cells. *Cell Biol Int* (2019).

174. Raslan, F. et al. Inhibition of bradykinin receptor B1 protects mice from focal brain injury by reducing blood-brain barrier leakage and inflammation. *J Cereb Blood Flow Metab* **30**, 1477-1486 (2010).

175. Kenne, E. et al. Neutrophils engage the kallikrein-kinin system to open up the endothelial barrier in acute inflammation. *FASEB journal : official publication of the Federation of American Societies for Experimental Biology* **33**, 2599-2609 (2019).

176. Matus, C.E. et al. Activation of the human keratinocyte B1 bradykinin receptor induces expression and secretion of metalloproteases 2 and 9 by transactivation of epidermal growth factor receptor. *Exp Dermatol* **25**, 694-700 (2016).

177. Dunsmore, S.E. & Rannels, D.E. Extracellular matrix biology in the lung. *Am J Physiol* **270**, L3-27 (1996).

178. Vu, T.H. Don't mess with the matrix. *Nat Genet* **28**, 202-203 (2001).

179. Greenlee, K.J., Werb, Z. & Kheradmand, F. Matrix metalloproteinases in lung: multiple, multifarious, and multifaceted. *Physiol Rev* **87**, 69-98 (2007).

180. Vaisar, T. et al. MMP-9 sheds the beta2 integrin subunit (CD18) from macrophages. *Molecular & cellular proteomics : MCP* **8**, 1044-1060 (2009).

181. Li, C. et al. Development of atopic dermatitis-like skin disease from the chronic loss of epidermal caspase-8. *Proc Natl Acad Sci U S A* **107**, 22249-22254 (2010).

182. Symowicz, J. et al. Engagement of collagen-binding integrins promotes matrix metalloproteinase-9-dependent E-cadherin ectodomain shedding in ovarian carcinoma cells. *Cancer Res* **67**, 2030-2039 (2007).

183. Wang, J.H. Blocking of Kinin B1 Receptor: A Promising Way for the Treatment of Acute Lung Injury. *Crit Care Med* **43**, 2520-2522 (2015).

184. Austinat, M. et al. Blockade of bradykinin receptor B1 but not bradykinin receptor B2 provides protection from cerebral infarction and brain edema. *Stroke* **40**, 285-293 (2009).

185. Göbel, K. et al. Blockade of the kinin receptor B1 protects from autoimmune CNS disease by reducing leukocyte trafficking. *J Autoimmun* **36**, 106-114 (2011).

186. Kassiri, Z. et al. Loss of angiotensin-converting enzyme 2 accelerates maladaptive left ventricular remodeling in response to myocardial infarction. *Circ Heart Fail* **2**, 446-455 (2009).

187. Elhelw, D.S. et al. Ectopic delivery of miR-200c diminishes hepatitis C virus infectivity through transcriptional and translational repression of Occludin. *Arch Virol* **162**, 3283-3291 (2017).

188. Rawat, M. et al. IL1B Increases Intestinal Tight Junction Permeability by Up-regulation of MIR200C-3p, Which Degrades Occludin mRNA. *Gastroenterology* **159**, 1375-1389 (2020).

189. Wilson, S. et al. The membrane-anchored serine protease, TMPRSS2, activates PAR-2 in prostate cancer cells. *Biochem J* **388**, 967-972 (2005).

190. Su, X., Camerer, E., Hamilton, J.R., Coughlin, S.R. & Matthay, M.A. Protease-activated receptor-2 activation induces acute lung inflammation by neuropeptide-dependent mechanisms. *J Immunol* **175**, 2598-2605 (2005).

191. Ma, Y. et al. [Role of SIRT1 in the protection of intestinal epithelial barrier under hypoxia and its mechanism]. *Zhonghua Wei Chang Wai Ke Za Zhi* **17**, 602-606 (2014).

192. Kowluru, R.A., Santos, J.M. & Zhong, Q. Sirt1, a negative regulator of matrix metalloproteinase-9 in diabetic retinopathy. *Invest Ophthalmol Vis Sci* **55**, 5653-5660 (2014).

193. Herrero, R. et al. Fas activation alters tight junction proteins in acute lung injury. *Thorax* **74**, 69-82 (2019).

194. Schickel, R., Park, S.M., Murmann, A.E. & Peter, M.E. miR-200c regulates induction of apoptosis through CD95 by targeting FAP-1. *Molecular cell* **38**, 908-915 (2010).

195. de Vries, N.A. et al. Prolonged Ezh2 Depletion in Glioblastoma Causes a Robust Switch in Cell Fate Resulting in Tumor Progression. *Cell Rep* **10**, 383-397 (2015).

196. Jian, M.Y., King, J.A., Al-Mehdi, A.B., Liedtke, W. & Townsley, M.I. High vascular pressure-induced lung injury requires P450 epoxygenase-dependent activation of TRPV4. *Am J Respir Cell Mol Biol* **38**, 386-392 (2008).

197. Willette, R.N. et al. Systemic activation of the transient receptor potential vanilloid subtype 4 channel causes endothelial failure and circulatory collapse: Part 2. *J Pharmacol Exp Ther* **326**, 443-452 (2008).

198. Thorneloe, K.S. et al. An orally active TRPV4 channel blocker prevents and resolves pulmonary edema induced by heart failure. *Sci Transl Med* **4**, 159ra148 (2012).

199. Mukaiyama, M., Yamasaki, Y., Usui, T. & Nagumo, Y. Transient receptor potential V4 channel stimulation induces reversible epithelial cell permeability in MDCK cell monolayers. *FEBS letters* **593**, 2250-2260 (2019).

200. Jie, P. et al. Blockage of transient receptor potential vanilloid 4 inhibits brain edema in middle cerebral artery occlusion mice. *Front Cell Neurosci* **9**, 141 (2015).

201. Vaziri, N.D., Yuan, J., Nazertehrani, S., Ni, Z. & Liu, S. Chronic kidney disease causes disruption of gastric and small intestinal epithelial tight junction. *Am J Nephrol* **38**, 99-103 (2013).

202. Lai, W.-T., Huang, Y.-H., Lo, M.-H. & Kuo, H.-C. Tight junction protein ZO-1 in Kawasaki disease. (2020).

203. Hong, J. Expression of Tight Junction Protein Occludin and ZO-1 in Lungs of COPD Rats. *Journal of Kunming Medical University* (2012).

204. Liu, Y. et al. Unfractionated Heparin Alleviates Sepsis-Induced Acute Lung Injury by Protecting Tight Junctions. *J Surg Res* **238**, 175-185 (2019).

205. Ueland, T. et al. Distinct and early increase in circulating MMP-9 in COVID-19 patients with respiratory failure. *J Infect* **81**, e41-e43 (2020).

206. Davey, A., McAuley, D.F. & O'Kane, C.M. Matrix metalloproteinases in acute lung injury: mediators of injury and drivers of repair. *Eur Respir J* **38**, 959-970 (2011).

207. Hsu, A.T. et al. Kinetics and Role of Plasma Matrix Metalloproteinase-9 Expression in Acute Lung Injury and the Acute Respiratory Distress Syndrome. *Shock* **44**, 128-136 (2015).

208. Turner, R.J. & Sharp, F.R. Implications of MMP9 for Blood Brain Barrier Disruption and Hemorrhagic Transformation Following Ischemic Stroke. *Front Cell Neurosci* **10**, 56 (2016).

209. Brilha, S. et al. Matrix metalloproteinase-9 activity and a downregulated Hedgehog pathway impair blood-brain barrier function in an in vitro model of CNS tuberculosis. *Sci Rep* **7**, 16031 (2017).

210. Pulido-Olmo, H. et al. Role of matrix metalloproteinase-9 in chronic kidney disease: a new biomarker of resistant albuminuria. *Clin Sci (Lond)* **130**, 525-538 (2016).

211. Kling, K.M., Lopez-Rodriguez, E., Pfarrer, C., Mühlfeld, C. & Brandenberger, C. Aging exacerbates acute lung injury-induced changes of the air-blood barrier, lung function, and inflammation in the mouse. *Am J Physiol Lung Cell Mol Physiol* **312**, L1-l12 (2017).

212. Michalick, L. et al. Plasma mediators in patients with severe COVID-19 cause lung endothelial barrier failure. *Eur Respir J* **57** (2021).

213. Li, X. et al. Angiotensin converting enzyme-2 is protective but downregulated in human and experimental lung fibrosis. *Am J Physiol Lung Cell Mol Physiol* **295**, L178-185 (2008).

214. Wang, L., Wang, Y., Yang, T., Guo, Y. & Sun, T. Angiotensin-Converting Enzyme 2 Attenuates Bleomycin-Induced Lung Fibrosis in Mice. *Cellular physiology and biochemistry : international journal of experimental cellular physiology, biochemistry, and pharmacology* **36**, 697-711 (2015).

215. Rodrigues Prestes, T.R., Rocha, N.P., Miranda, A.S., Teixeira, A.L. & Simoes, E.S.A.C. The Anti-Inflammatory Potential of ACE2/Angiotensin-(1-7)/Mas Receptor Axis: Evidence from Basic and Clinical Research. *Curr Drug Targets* **18**, 1301-1313 (2017).

216. Kazemi-Bajestani, S.M., Patel, V.B., Wang, W. & Oudit, G.Y. Targeting the ACE2 and Apelin Pathways Are Novel Therapies for Heart Failure: Opportunities and Challenges. *Cardiol Res Pract* **2012**, 823193 (2012).

217. Shenoy, V., Qi, Y., Gupta, D., Katovich, M. & Raizada, M. Small moleculae ACE2 activator, diminazene aceturate attenuates bleomycin-induced pulmonary fibrosis. *European Respiratory Journal* **40**, P1765 (2012).

218. Ferreira, A.J. et al. Angiotensin-converting enzyme 2 activation protects against hypertension-induced cardiac fibrosis involving extracellular signal-regulated kinases. *Exp Physiol* **96**, 287-294 (2011).

219. Ricupero, D.A., Romero, J.R., Rishikof, D.C. & Goldstein, R.H. Des-Arg(10)-kallidin engagement of the B1 receptor stimulates type I collagen synthesis via stabilization of connective tissue growth factor mRNA. *J Biol Chem* **275**, 12475-12480 (2000).

220. Klein, J. et al. Delayed blockade of the kinin B1 receptor reduces renal inflammation and fibrosis in obstructive nephropathy. *FASEB journal : official publication of the Federation of American Societies for Experimental Biology* **23**, 134-142 (2009).

221. Klein, J. et al. Blockade of the kinin B1 receptor ameloriates glomerulonephritis. *J Am Soc Nephrol* **21**, 1157-1164 (2010).

222. Wang, P.H. et al. Deletion of bradykinin B1 receptor reduces renal fibrosis. *Int Immunopharmacol* **9**, 653-657 (2009).

223. Westermann, D. et al. Gene deletion of the kinin receptor B1 attenuates cardiac inflammation and fibrosis during the development of experimental diabetic cardiomyopathy. *Diabetes* **58**, 1373-1381 (2009).

224. Venkataraman, T., Coleman, C.M. & Frieman, M.B. Overactive Epidermal Growth Factor Receptor Signaling Leads to Increased Fibrosis after Severe Acute Respiratory Syndrome Coronavirus Infection. *J Virol* **91** (2017).

225. Abdallah, R.T. et al. Plasma kallikrein promotes epidermal growth factor receptor transactivation and signaling in vascular smooth muscle through direct activation of protease-activated receptors. *J Biol Chem* **285**, 35206-35215 (2010).

226. Martinelli, M. et al. A role for epidermal growth factor receptor in idiopathic pulmonary fibrosis onset. *Mol Biol Rep* **38**, 4613-4617 (2011).

227. Wygrecka, M. et al. Role of protease-activated receptor-2 in idiopathic pulmonary fibrosis. *Am J Respir Crit Care Med* **183**, 1703-1714 (2011).

228. Chung, H., Ramachandran, R., Hollenberg, M.D. & Muruve, D.A. Proteinase-activated receptor-2 transactivation of epidermal growth factor receptor and transforming growth factor-β receptor signaling pathways contributes to renal fibrosis. *J Biol Chem* **288**, 37319-37331 (2013).

229. Ramachandran, S. et al. Hepatitis C virus induced miR200c down modulates FAP-1, a negative regulator of Src signaling and promotes hepatic fibrosis. *PLoS One* **8**, e70744 (2013).

230. Chen, J. et al. [Recent advances in miR-200c and fibrosis in organs]. *Zhong Nan Da Xue Xue Bao Yi Xue Ban* **42**, 226-232 (2017).

231. Ottaviani, L.M.J., R. P.; Sansonetti, M.; Sampaio-Pinto, V.; Halkein, J.; el Azzouzi, H.; Olieslagers, S., Nascimento, D. S.; de Windt, L. J.; da Costa Martins, P. A.; in Circulation Research, Vol. 125 (2019).

232. Ottaviani, L., Sansonetti, M. & da Costa Martins, P.A. Myocardial cell-to-cell communication via microRNAs. *Noncoding RNA Res* **3**, 144-153 (2018).

233. Li, S.W. et al. SARS coronavirus papain-like protease induces Egr-1-dependent up-regulation of TGF-β1 via ROS/p38 MAPK/STAT3 pathway. *Sci Rep* **6**, 25754 (2016).

234. Bhattacharyya, S., Fang, F., Tourtellotte, W. & Varga, J. Egr-1: new conductor for the tissue repair orchestra directs harmony (regeneration) or cacophony (fibrosis). *J Pathol* **229**, 286-297 (2013).

235. Hillman, Y., Mazkereth, N., Farberov, L., Shomron, N. & Fishelson, Z. Regulation of Complement-Dependent Cytotoxicity by MicroRNAs miR-200b, miR-200c, and miR-217. *J Immunol* **196**, 5156-5165 (2016).

236. Lee, W.J. et al. Mortalin deficiency suppresses fibrosis and induces apoptosis in keloid spheroids. *Sci Rep* **7**, 12957 (2017).

237. Zeng, Z. et al. Activation and overexpression of Sirt1 attenuates lung fibrosis via P300. *Biochem Biophys Res Commun* **486**, 1021-1026 (2017).

238. Chu, H. et al. Sirtuin1 Protects against Systemic Sclerosis-related Pulmonary Fibrosis by Decreasing Proinflammatory and Profibrotic Processes. *Am J Respir Cell Mol Biol* **58**, 28-39 (2018).

239. Shao, M. et al. Exogenous angiotensin (1-7) directly inhibits epithelial-mesenchymal transformation induced by transforming growth factor-β1 in alveolar epithelial cells. *Biomedicine & Pharmacotherapy* **117**, 109193 (2019).

240. Zhang, Y.Q. et al. Resveratrol ameliorates lipopolysaccharide-induced epithelial mesenchymal transition and pulmonary fibrosis through suppression of oxidative stress and transforming growth factor-β1 signaling. *Clin Nutr* **34**, 752-760 (2015).

241. Hung, C.H., Chan, S.H., Chu, P.M., Lin, H.C. & Tsai, K.L. Metformin regulates oxLDL-facilitated endothelial dysfunction by modulation of SIRT1 through repressing LOX-1-modulated oxidative signaling. *Oncotarget* **7**, 10773-10787 (2016).

242. Rangarajan, S. et al. Metformin reverses established lung fibrosis in a bleomycin model. *Nat Med* **24**, 1121-1127 (2018).

243. Galeotti, C. & Bayry, J. Autoimmune and inflammatory diseases following COVID-19. *Nat Rev Rheumatol* **16**, 413-414 (2020).

244. Rodríguez, Y. et al. Autoinflammatory and autoimmune conditions at the crossroad of COVID-19. *J Autoimmun* **114**, 102506 (2020).

245. Riphagen, S., Gomez, X., Gonzalez-Martinez, C., Wilkinson, N. & Theocharis, P. Hyperinflammatory shock in children during COVID-19 pandemic. *Lancet* **395**, 1607-1608 (2020).

246. Uppal, N.N. et al. De Novo ANCA-Associated Vasculitis With Glomerulonephritis in COVID-19. *Kidney Int Rep* **5**, 2079-2083 (2020).

247. Zhang, Y. et al. Coagulopathy and Antiphospholipid Antibodies in Patients with Covid-19. *N Engl J Med* **382**, e38 (2020).

248. Harzallah, I., Debliquis, A. & Drénou, B. Lupus anticoagulant is frequent in patients with Covid-19. *J Thromb Haemost* **18**, 2064-2065 (2020).

249. Bowles, L. et al. Lupus Anticoagulant and Abnormal Coagulation Tests in Patients with Covid-19. *N Engl J Med* **383**, 288-290 (2020).

250. Helms, J. et al. High risk of thrombosis in patients with severe SARS-CoV-2 infection: a multicenter prospective cohort study. *Intensive Care Med* **46**, 1089-1098 (2020).

251. Woodruff, M.C. et al. Extrafollicular B cell responses correlate with neutralizing antibodies and morbidity in COVID-19. *Nat Immunol* **21**, 1506-1516 (2020).

252. Tipton, C.M. et al. Diversity, cellular origin and autoreactivity of antibody-secreting cell population expansions in acute systemic lupus erythematosus. *Nat Immunol* **16**, 755-765 (2015).

253. William, J., Euler, C., Christensen, S. & Shlomchik, M.J. Evolution of autoantibody responses via somatic hypermutation outside of germinal centers. *Science (New York, N.Y.)* **297**, 2066-2070 (2002).

254. Schulze-Topphoff, U. et al. Activation of kinin receptor B1 limits encephalitogenic T lymphocyte recruitment to the central nervous system. *Nat Med* **15**, 788-793 (2009).

255. Hara, D.B. et al. The relevance of kinin B1 receptor upregulation in a mouse model of colitis. *Br J Pharmacol* **154**, 1276-1286 (2008).

256. Marceau, F. & Regoli, D. Therapeutic options in inflammatory bowel disease: experimental evidence of a beneficial effect of kinin B1 receptor blockade. *Br J Pharmacol* **154**, 1163-1165 (2008).

257. Prat, A. et al. Bradykinin B1 receptor expression and function on T lymphocytes in active multiple sclerosis. *Neurology* **53**, 2087-2092 (1999).

258. Qin, L. et al. Bradykinin 1 receptor blockade subdues systemic autoimmunity, renal inflammation, and blood pressure in murine lupus nephritis. *Arthritis Res Ther* **21**, 12 (2019).

259. Bertram, C.M. et al. Expression of kinin B1 and B2 receptors in immature, monocyte-derived dendritic cells and bradykinin-mediated increase in intracellular Ca2+ and cell migration. *Journal of leukocyte biology* **81**, 1445-1454 (2007).

260. Qian, C. et al. Differential Expression of MiR-106b-5p and MiR-200c-3p in Newly Diagnosed Versus Chronic Primary Immune Thrombocytopenia Patients Based on Systematic Analysis. *Cellular physiology and biochemistry : international journal of experimental cellular physiology, biochemistry, and pharmacology* **45**, 301-318 (2018).

261. Li, X. et al. Enhanced apoptosis and senescence of bone-marrow-derived mesenchymal stem cells in patients with systemic lupus erythematosus. *Stem Cells Dev* **21**, 2387-2394 (2012).

262. Gu, Z. et al. Upregulation of p16INK4A promotes cellular senescence of bone marrow-derived mesenchymal stem cells from systemic lupus erythematosus patients. *Cell Signal* **24**, 2307-2314 (2012).

263. Dambha-Miller, H. et al. Currently prescribed drugs in the UK that could upregulate or downregulate ACE2 in COVID-19 disease: a systematic review. *BMJ Open* **10**, e040644 (2020).

264. Yamakawa, K. et al. Study of immunological mechanism in dilated cardiomyopathy. *Jpn Circ J* **51**, 665-675 (1987).

265. van de Veerdonk, F.L. et al. Outcomes Associated With Use of a Kinin B2 Receptor Antagonist Among Patients With COVID-19. *JAMA Netw Open* **3**, e2017708 (2020).

266. Urwyler, P. et al. Treatment of COVID-19 With Conestat Alfa, a Regulator of the Complement, Contact Activation and Kallikrein-Kinin System. *Front Immunol* **11**, 2072 (2020).

267. Cohen, J.B. et al. Continuation versus discontinuation of renin-angiotensin system inhibitors in patients admitted to hospital with COVID-19: a prospective, randomised, open-label trial. *Lancet Respir Med* **9**, 275-284 (2021).

268. Lopes, R.D. et al. Effect of Discontinuing vs Continuing Angiotensin-Converting Enzyme Inhibitors and Angiotensin II Receptor Blockers on Days Alive and Out of the Hospital in Patients Admitted With COVID-19: A Randomized Clinical Trial. *Jama* **325**, 254-264 (2021).

269. Stewart, G.M. et al. Targeting pulmonary capillary permeability to reduce lung congestion in heart failure: a randomized, controlled pilot trial. *Eur J Heart Fail* **22**, 1641-1645 (2020).

270. Goyal, N. et al. Clinical Pharmacokinetics, Safety, and Tolerability of a Novel, First-in-Class TRPV4 Ion Channel Inhibitor, GSK2798745, in Healthy and Heart Failure Subjects. *Am J Cardiovasc Drugs* **19**, 335-342 (2019).

271. Entrenas Castillo, M. et al. "Effect of calcifediol treatment and best available therapy versus best available therapy on intensive care unit admission and mortality among patients hospitalized for COVID-19: A pilot randomized clinical study". *J Steroid Biochem Mol Biol* **203**, 105751 (2020).

272. Meizlish, M.L. et al. Intermediate-dose anticoagulation, aspirin, and in-hospital mortality in COVID-19: A propensity score-matched analysis. *Am J Hematol* **96**, 471-479 (2021).

273. Viecca, M., Radovanovic, D., Forleo, G.B. & Santus, P. Enhanced platelet inhibition treatment improves hypoxemia in patients with severe Covid-19 and hypercoagulability. A case control, proof of concept study. *Pharmacol Res* **158**, 104950 (2020).

274. Wang, J. et al. Association of Metformin with Susceptibility to COVID-19 in People with Type 2 Diabetes. *J Clin Endocrinol Metab* **106**, 1255-1268 (2021).

275. Cheng, X. et al. Effects of metformin, insulin on COVID-19 patients with pre-existed type 2 diabetes: A multicentral retrospective study. *Life Sci* **275**, 119371 (2021).

276. Ghany, R. et al. Metformin is associated with lower hospitalizations, mortality and severe coronavirus infection among elderly medicare minority patients in 8 states in USA. *Diabetes Metab Syndr* **15**, 513-518 (2021).

277. Bramante, C.T. et al. Outpatient metformin use is associated with reduced severity of COVID-19 disease in adults with overweight or obesity. *J Med Virol* **93**, 4273-4279 (2021).

278. Crouse, A.B. et al. Metformin Use Is Associated With Reduced Mortality in a Diverse Population With COVID-19 and Diabetes. *Frontiers in endocrinology* **11**, 600439 (2020).

279. Lalau, J.D. et al. Metformin use is associated with a reduced risk of mortality in patients with diabetes hospitalised for COVID-19. *Diabetes Metab* **47**, 101216 (2020).

280. Lally, M.A. et al. Metformin is Associated with Decreased 30-Day Mortality Among Nursing Home Residents Infected with SARS-CoV2. *J Am Med Dir Assoc* **22**, 193-198 (2021).

281. Li, J. et al. Metformin Use in Diabetes Prior to Hospitalization: Effects on Mortality in Covid-19. *Endocr Pract* **26**, 1166-1172 (2020).

282. Hariyanto, T.I. & Kurniawan, A. Metformin use is associated with reduced mortality rate from coronavirus disease 2019 (COVID-19) infection. *Obes Med* **19**, 100290 (2020).

283. Lukito, A.A. et al. The Effect of Metformin Consumption on Mortality in Hospitalized COVID-19 patients: a systematic review and meta-analysis. *Diabetes Metab Syndr* **14**, 2177-2183 (2020).

284. Rakhmat, II et al. Dipeptidyl peptidase-4 (DPP-4) inhibitor and mortality in coronavirus disease 2019 (COVID-19) - A systematic review, meta-analysis, and meta-regression. *Diabetes Metab Syndr* **15**, 777-782 (2021).
